# Gene Ontology Groups and Signaling Pathways Regulating the Process of Avian Satellite Cell Differentiation

**DOI:** 10.3390/genes13020242

**Published:** 2022-01-27

**Authors:** Afsaneh Golkar-Narenji, Paweł Antosik, Shelly Nolin, Marcin Rucinski, Karol Jopek, Agnieszka Zok, Jarosław Sobolewski, Maurycy Jankowski, Maciej Zdun, Dorota Bukowska, Katarzyna Stefańska, Jędrzej M. Jaśkowski, Hanna Piotrowska-Kempisty, Paul Mozdziak, Bartosz Kempisty

**Affiliations:** 1 Prestage Department of Poultry Sciences, North Carolina State University, Raleigh, NC 27695, USA; agmozdzi@ncsu.edu (A.G.-N.); snjones@ncsu.edu (S.N.); pemozdzi@ncsu.edu (P.M.); 2 Department of Veterinary Surgery, Institute of Veterinary Medicine, Nicolaus Copernicus University in Torun, 87-100 Torun, Poland; pantosik@umk.pl; 3 Department of Histology and Embryology, Poznan University of Medical Sciences, 60-701 Poznan, Poland; marcinruc@ump.edu.pl (M.R.); karoljopek@ump.edu.pl (K.J.); k.stefanska94@o2.pl (K.S.); 4 Division of Philosophy of Medicine and Bioethics, Poznan University of Medical Sciences, 60-701 Poznan, Poland; agzok@ump.edu.pl; 5 Department of Social Sciences and Humanities, Poznan University of Medical Sciences, 60-701 Poznan, Poland; 6 Department of Public Health Protection and Animal Welfare, Institute of Veterinary Medicine, Nicolaus Copernicus University in Torun, 87-100 Torun, Poland; jsobolewski@umk.pl; 7 Department of Anatomy, Poznan University of Medical Sciences, 60-701 Poznan, Poland; mjankowski@ump.edu.pl; 8 Department of Basic and Preclinical Sciences, Institute of Veterinary Medicine, Nicolaus Copernicus University in Torun, 87-100 Torun, Poland; maciejzdun@umk.pl (M.Z.); hpiotrow@ump.edu.pl (H.P.-K.); 9 Department of Diagnostics and Clinical Sciences, Institute of Veterinary Medicine, Nicolaus Copernicus University in Torun, 87-100 Torun, Poland; dbukowska@umk.pl (D.B.); jmjaskowski@umk.pl (J.M.J.); 10 Department of Toxicology, Poznan University of Medical Sciences, 60-701 Poznan, Poland

**Keywords:** satellite cell, differentiation, gene markers, signalling pathways

## Abstract

Modern science is becoming increasingly committed to environmentally friendly solutions, mitigating the impact of the developing human civilisation on the environment. One of the leading fields aimed at sustainable agriculture is in vitro meat production. Cellular agriculture aims to provide a source of animal-free meat products, which would decrease worldwide nutritional dependency on animal husbandry, thereby reducing the significant impact of this industry on Earth’s climate. However, while some studies successfully produced lab-based meat on a small scale, scalability of this approach requires significant optimisation of the methodology in order to ensure its viability on an industrial scale. One of the methodological promises of in vitro meat production is the application of cell suspension-based bioreactors. Hence, this study focused on a complex transcriptomic comparison of adherent undifferentiated, differentiated and suspension-cultured myosatellite cells, aiming to determine the effects of different culture methods on their transcriptome. Modern next-generation sequencing (RNAseq) was used to determine the levels of transcripts in the cultures’ cell samples. Then, differential expression and pathway analyses were performed using bionformatical methods. The significantly regulated pathways included: EIF2, mTOR, GP6, integrin and HIFα signalling. Differential regulation of gene expression, as well as significant enrichment and modulation of pathway activity, suggest that suspension culture potentially promotes the ex vivo-associated loss of physiological characteristics and gain of plasticity. Therefore, it seems that suspension cultures, often considered the desired method for in vitro meat production, require further investigation to fully elucidate their effect on myosatellite cells and, therefore, possibly enable their easier scalability to ensure suitability for industrial application.

## 1. Introduction

The livestock sector plays a significant role in deforestation, biodiversity loss and climate change. Livestock farming is a significant contributor to the water footprint, water pollution and water scarcity. Mekonnen and Hoekstra [1] show that the water footprint (WF) of each animal product is larger than the WF of alternative crops with equivalent nutritional value. For example, the average WF per calorie for beef (10 L kcal^−1^) is 20 times greater than for cereals and starchy roots (0.5 L kcal^−1^ ). The WF per gram of protein for milk, eggs and chicken meat (approximately 30 L g^−1^ protein) is 1.5 times greater than pulses (20 L g^−1^ protein). It is estimated that livestock is responsible for 18% of anthropogenic greenhouse gas emissions (in CO_2_ equivalents) [2].

In the modern day, environmentally friendly technologies, aiming to reduce the burden of human civilization on the planet, are attracting the attention of the scientific community. Biomanufacturing of meat-based products has attracted significant attention due to the noteworthy impact of the livestock industry on the environment, as well as the resulting reduction in the ethical burden of meat consumption [3]. Meat-based diets are common worldwide, a complete transition of the population to plant-based alternatives would require immense global effort and a significant amount of time [4]. Hence, lab grown meat is proposed as a substitute for animal products to eliminate the environmental and ethical burden of meat consumption without the need to force people to transition directly to plant-based alternatives that may not present a similar nutritional value and/or taste [5].

While the concept of lab-grown meat is certainly appealing, there are several obstacles that must be overcome before it can be applied on the scale necessary to provide nutrition to the growing human population. The currently experimental “animal-free” meat products are often associated with significant cost, large energy consumption and low efficiency of production. These facts would likely contradict the purpose of the development of such technology, as the production of “animal-free” meat products areis still associated with significant energy expenditure [6]. Furthermore, the current methods of lab-grown meat production are still relatively simple and do not try to mimic the consistency or exact taste of “real” meat (as the lab-grown meat contains a range of other tissues, such as adipose and connective). Nonetheless, there are large variety of meat-based products currently available on the market [7].

Therefore, a large amount of studies is needed to ensure the viability of in vitro meat production for achieving the purpose of development of such technology. There are several challenges associated with this approach, spanning topics from achieving industrial viability to lowering the energy cost of production to decreasing the price for the end-point customer [8]. Furthermore, the generation of organized artificial tissue is an immensely complex task, requiring methods such as edible scaffold generation, development of bioprinting techniques or successful administration of different population specific media to highly heterogenous cell cultures (or design of a one-fits-all nutrient mix) [9]. Additionally, it also needs to be mentioned that current culture nutrition supplements, such as FBS, BSA or HS, remain sourced from livestock. Hence, full independence from animal products would also require the introduction of novel media supplements, appropriately scalable to facilitate adequate in vitro meat production [10].

Nevertheless, while the topic of lab-grown meat is not exactly new, the current research is still in relatively early stages and mostly focuses on finding the perfect source of stem cells for the growth of lab-produced meat, as well as determining the best technology for its propagation [6]. There are several approaches to muscle cell culture, including adherent as well as a range of suspension-based cultures and bioreactors [7]. However, knowledge of the molecular differences between the cells cultured using different methods is still lacking, with studies providing insight into the mechanisms governing satellite stem cell (the source of muscle cells in vivo) growth and differentiation in vitro, which potentially holds the key to scalability of lab-grown meat production [3].

The aim of this study was to evaluate, using next generation sequencing, the molecular effect of different types of long-term culture on turkey satellite cells. A thorough analysis of the cellular response to suspension cultures should provide further information about their molecular effects on myosatellites, thereby shedding additional light on their suitability for industrial applications. A comparison of gene expression was performed between undifferentiated satellite cells, differentiated satellite cells and suspension-cultured cells.

## 2. Material and Methods

### 2.1. Animals

All animal experiments were approved by the North Carolina State University Institutional Animal Care and Use Committee. Newborn turkeys (one day old) were killed by cervical dislocation following approved IACUC regulations.

### 2.2. The Study Design

#### 2.2.1. Isolation, Culture and Differentiation of Satellite Cells

Satellite cells were isolated from breast muscle (pectroralis thoracicus) of new born turkeys and cultured for two weeks as undifferentiated. A complex characterisation of the isolated cell population has already been described in previous works of the co-authors [11,12]. At the end of two weeks, the cells were divided into three different groups: undifferentiated, differentiated (identified through a downregulation of self-renewal genes and no telomerase activity, as described in a previous article from our group [11]) and suspension cultured. This means that some cells were kept to grow as undifferentiated (undifferentiated group) and cultured as adherent, some cells were fused to form myotubes (differentiated group) as the adherent culture and some cells were cultured as suspension (suspended group). In short, satellite cells were isolated from 1-day old turkeys using sterile forceps and scissors, mechanically disassociated with sterile forceps and incubated for 30 min in warm (37 °C) 0.17% Trypsin+0.085% collagenase solution in Hank’s balanced salt for satellite cell liberation. Subsequently, the tissue was washed twice with turkey plating medium (TPM), which consisted of 89% Dulbecco’s Modified Eagle’s Medium (DMEM), 10% horse serum (Gibco, Grand Island, NY, USA) and 1% penicillin streptomycin fungizone (Gibco). The tissue was resuspended in TPM, triturated through a Pasteur pipette followed by an 18-gauge needle; the cell concentration was estimated using a hemocytometer, and the cells for the adherent cultures were plated on 0.1% gelatin-coated 100-mm cell culture dishes at a concentration of cells per dish. After a 24-h attachment period, the TPM was replaced with turkey growth medium (TGRM), which consisted of 84% McCoy’s 5A, 15% chicken serum (Gibco) and 1% penicillin streptomycin fungizone. The satellite cells were cultured for two weeks before the growth media were replaced with differentiating media DMEM-4% horse serum. Moreover, additional cells were washed with PBS, detached through incubation in EDTA at 37 °C for 5 min and directly inoculated to an Erlenmeyer flask containing Mcoy’s 5A and 15% chicken serum, in order to culture the growing cells as a suspension under rotation at 110 rpm. All of the groups were cultured for several passages until they were viable and growing continuously. The suspended cells were cultured for three months, after which RNA isolation was performed. The experiment was repeated three times, and the isolated total RNA was pooled together for each group. Then, the pooled RNA for each of the experimental groups was subjected to RNA sequencing with bioinformatic evaluations to compare the viability and self-renewal ability of different groups. The adherent undifferentiated group was compared to the adherent differentiated group as well as to the cells cultured in suspension.

#### 2.2.2. RNA Isolation

Cells from one confluent T75 flask for each sample were digested in 500 µL of trizol and incubated at room temperature for 5 min. In the next step, 100 µL of chloroform were added, and the sample was shaken by vortex for 1 min and incubated for 5 min. The resulting mix was centrifuged at 12,000 rcf for 20 min. The upper aqueous phase was transferred to a new vial on ice and cooled isopropanol (isopropyl alcohol) in the equal amount was added, vortexed and incubated on ice for 30 min. The vial was centrifuged in a refrigerated centrifuge for 20 min. When the RNA pellet was visible, supernatants were removed, and, in order to remove isopropanol, it was washed twice with 75% ethanol. The ethanol was removed, and the pellet was dried at room temperature for 30 min. Afterwards, the dried pellet was dissolved in 25 µL DEPC water.

#### 2.2.3. RNAseq

The amount of isolated RNA was determined by spectrophotometric measurement of absorbance at 260 nm. The purity of the extracted RNA was calculated using an absorbance ratio of 260/280 nm (NanoDrop spectrophotometer, Thermo Scientific, Waltham, MA, USA). The integrity and quality of the isolated RNA were examined with the Agilent 2100 Bioanalyzer (Agilent Technologies, Inc., Santa Clara, CA, USA). The resultant RNA integrity numbers (RINs) varied from 8.5 to 10, with an average of 9.2. RNA.

Five hundred nanograms of total RNA from each sample were submitted to the North Carolina State University Genomics Research Laboratory for library preparation and sequencing on the Illumina NovoSeq 6000 (Illumina, Inc., San Diego, CA, USA). Each sample generated 35–40 million sequence reads. All of the raw data were uploaded to the GEO database (GSE193361).

#### 2.2.4. Bioinformatics Analysis

The demultiplexing of the sequencing reads was carried out using Illumina bcl2fastq (v.2.20) to obtain FASTQ files. The sequencing quality was controlled using the FastQC tool [13]. Then, adapters were trimmed with Skewer (version 0.2.2) [14]. Trimmed raw reads were aligned to the turkey reference genome (Meleagris_gallopavio. Turkey 2.01) with the relevant GTF file downloaded from the Ensembl database. Alignment was carried out using STAR (version 2.5.2b) software [15]. Overall summarization results, including the number of successfully assigned reads with unnormalized counts, were performed using featureCounts [16]. In the next step, NOISeq R-package was used for quality control and quantitative analysis of the count data [17], where count values were normalized to RPKM (reads per kilobase million) and applied to the calculation of the essential parameters for the differences in expression between the compared groups. Based on the algorithm implemented in NOISeq, the most relevant parameters for calculating differential expression are the “M” value corresponding to the log2 ratio between two compared conditions (log2 (RPKM[first group]/RPKM[second group])), “D” value—the difference between conditions (RPKM [group with higher RPKM]—RPKM [group with lower RPKM]) and “*p*-value”. To avoid a type I error, we apply the Benjamini–Hochberg procedure for multiple testing correction. We assumed that the genes with a *p*-value below 0.05 were to be considered differentially expressed (DEGs). The overall transcriptomic profile between the compared groups was presented as a scatter plot, showing the total number of up- and downregulated genes. The names and expression change values f these genes are also included in the form of Table S1. The top ten up- and downregulated genes are presented in relevant tables (Table 1). The ENTREZ IDs for the turkey genes were mapped to the corresponding ENTREZ IDs for the human genes. For this purpose, we used the “biomaRt” BioConductor library [18].

#### 2.2.5. Overall Enrichment Analysis

Lists of DEGS ENTREZ IDs from each comparison were functionally analysed using the Metascape tool. Metascape combines functional enrichment, interactome analysis, gene annotation and membership search to leverage over 40 independent knowledgebases [19]. The overlapping between genes from different input gene lists was visualized using a circos plot. Terms with a cumulative hypergeometric *p* < 0.01, a minimum count of 3 and an enrichment factor > 1.5 were collected and grouped into clusters based on their membership similarities. The 20 top enriched terms from all statistically enriched ontology terms (determined on: canonical pathways, gene ontology KEGG) were presented as a heatmap with cumulative hypergeometric *p*-values. The significantly enriched terms were hierarchically clustered based on the statistical Kappa similarity between their gene memberships. The term with the lowest *p*-value per cluster was considered a generalized cluster name (cluster ID). The obtained clusters were shown as a network of enriched terms, where the main nodes are superimposed by colour-coded pie charts based on the identities from the gene lists from individual comparisons.

The enrichment analysis of the gene ontology terms was confirmed using the “clusterProfiler” library [20]. The analysis was performed separately for each comparison applied hypergeometric statistical test. Reference GO annotation data were obtained directly from the human annotation library “org.Hs.eg.db”. Enriched GO BP terms were visualized using an enrichment map—a network where edges connect overlapping gene sets, leading to the identification of common functional GO clusters.

#### 2.2.6. Protein–Protein Interaction (PPI) Networks

Protein–protein interactions (PPIs) among all input gene lists were constructed from the PPI database by Metascape to form PPI networks. PPI enrichment analysis was carried out according to interaction data from the following databases: STRING [21], BioGrid [22] and OmniPath [23]. In a situation where the PPI network contains more than three nodes, the Molecular Complex Detection (MCODE) algorithm was calculated to identify key clusters of genes within PPI [24]. For each obtained network, a list of the top three MCODE terms with the lowest *p*-values was generated and assigned a unique colour.

#### 2.2.7. Assignment of Differentially Expressed Genes

The mapped ENTREZ id and log2 ratio (“M”) from differentially expressed genes (DEGs) were subjected to functional annotation using the Database for Annotation, Visualization and Integrated Discovery (DAVID) bioinformatics tool [25]. The required data for the DEGs were uploaded to DAVID via the “RDAVIDWebService” BioConductor library [26], where each DEG was assigned to relevant GO terms with subsequent selection of significantly enriched GO terms from the GO BP Direct database. The *p*-values of the selected GO terms were corrected using the Benjamini–Hochberg correction described as adjusted *p*-values. Relevant GO ontological groups with adjusted *p*-values below 0.05 and N per group > 5 were visualized using a bubble plot. A detailed analysis of genes belonging to the significantly enriched ontology groups referring to muscle was presented as a circos plot using the “GOplot” BioConductor library [27].

#### 2.2.8. Signalling Pathways Analysis

The “rWikiPathways” BioConductor library was used to check enrichment of DEGs, in particular, the signalling pathways [28]. The log2 ratio values of significantly changed genes were mapped by colours, where blue represents upregulated genes, and red represents downregulated genes. Genes below a predetermined cut-off value were uncoloured. Data for the signalling pathways were exported from R to Cytoscape (v. 3.7.2) using the “RCy3” library [29]. The box in the pathway that corresponds to a given gene was divided into three parts, coloured separately according to the gene expression comparisons.

#### 2.2.9. Ingenuity Pathway Analysis

Lists of DEG with *p* < 0.05 were used for further functional comparisons between adherent vs. suspension, adherent vs. differentiating and differentiating vs. suspension groups using Ingenuity Pathway Analysis (IPA, Qiagen, Hilden, Germany) to create figures of the enriched canonical pathways for each comparison. When possible, IPA’s database utilizes published data on molecular interactions in combination with the user expression data to generate enhanced pathway figures, which illustrate predictions about the states of pathway molecules that are not in the user dataset.

## 3. Results

**Figure 1:**
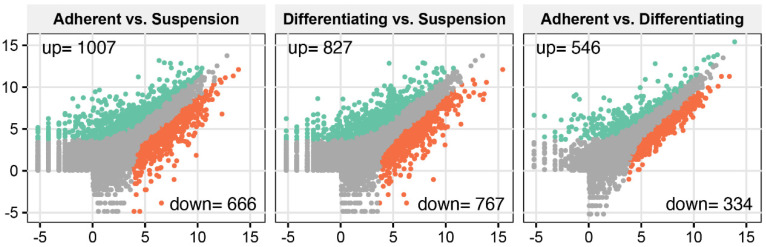
Total gene expression profiles in the following comparisons: adherent undifferentiated vs. suspension-cultured, adherent differentiated vs. suspension, adherent undifferentiated vs. differentiated. Each dot represents log2(RPKM) value from a single gene. Grey dots represent the genes below cut-off limit (*p* > 0.05). Green and red dots represent differentially expressed genes where green colour marked the stimulated genes, while red ones were inhibited. Stimulation or inhibition was determined in relation to the second group from each compared pair (occurring after vs. in the graph description).

To analyse transcriptome profile changes during differentiation of cultivated cells, we applied the Illumina-based RNAseq method. Sequencing and bioinformatic preprocessing resulted in the obtainment of data for 15,002 turkey genes. To determine differentially expressed genes, we applied NOISeg R-package where the log2 ratio between compared conditions (“M”), the difference between conditions (“D”) and *p*-values were calculated. The overall transcriptome profiles were shown as scatter plots (Figure 1). According to the accepted cut-off criteria (*p* < 0.05), in adherent undifferentiated cells, 1007 genes were upregulated, and 666 genes were downregulated in relation to suspension cells. In the comparison of adherent differentiated cells and suspension cells, it was shown that 827 genes were upregulated, whilst 767 were downregulated. In the third comparison between adherent undifferentiated and differentiated cells, we revealed that 546 genes were upregulated and 334 downregulated.

**Figure 2:**
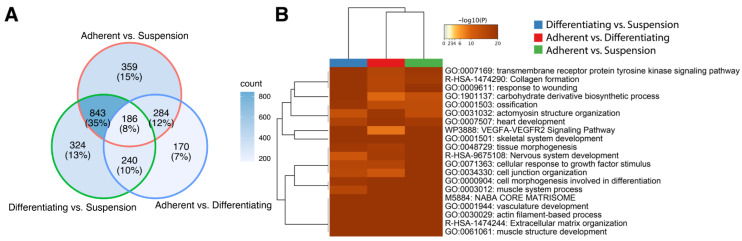
Metascape functional analysis of transcriptome profiles between: Adherent differentiated vs. Suspension, Adherent undifferentiated vs. Differentiated, Adherent undifferentiated vs. Suspension. (**A**) The Venn diagram shows overlapping of DEGs from different input gene lists. (**B**) Heatmap of the top 20 enriched terms across differentially expressed gene lists.

The ten genes of the highest and lowest log2 ratio values were presented in Table 1, which displays the gene symbol, gene name, PRKMs, log2 ratio between compared conditions (“M”), the difference between conditions (“D”) and *p*-value. The adherent vs. suspension comparison presented in the table of the top 20 genes includes: fibrinogen-like 2 (FGL2, log2(ratio) = −10.52), C1q and TNF related 2 (*C1QTNF2*, log2(ratio) = −10.45), calcium voltage-gated channel auxiliary subunit γ 3 (*CACNG3*, log2(ratio) = 10.40) and Wnt family member 10A (*WNT10A*, log2(ratio) = 9.34). In the differentiating vs. suspension comparison, we observed the strongest effect on the expression of the following genes: calcium voltage-gated channel auxiliary subunit γ 3 (*CACNG3*, log2(ratio) = 10.12), ADAM metallopeptidase domain 8 (*ADAM8*, log2(ratio) = 9.87), fibrinogen-like 2 (FGL2, log2(ratio) = −10.81) and proteolipid protein 1 (*PLP1*, log2(ratio) = −11.41). In the last groups compared, i.e., adherent vs. differentiating, we showed the strongest changes in expression of following genes: scavenger receptor class A member 3 (*SCARA3*, log2(ratio) = −11.87), aggrecan (*ACAN*, log2(ratio) = −10.74), opioid-binding protein/cell adhesion molecule-like (*OPCML*, log2(ratio) = 4.82), FAT atypical cadherin 3 (*FAT3*, log2(ratio) = 4.65). For overall enrichment analysis of all the differentially expressed genes, we applied Metacape—a powerful software that combines functional enrichment, interactome analysis, gene annotation and membership search to leverage over 40 independent knowledgebases [19]. We used three independent lists (adherent undifferentiated vs. suspension, adherent differentiated vs. suspension, adherent undifferentiated vs. differentiated) containing ENTREZ IDs for the differentially expressed genes. As can be observed in the Venn diagram (Figure 2A), many genes overlap between the compared experimental conditions. However, it is worth noting that the analysis did not consider the direction of expression changes, i.e., the stimulation or inhibition of gene expression. Then, we performed clusterization of the 20 top significantly enriched terms based on their cumulative hypergeometric *p*-values. The results of this analysis were shown as a heatmap. In the analysis performed, the most enriched terms refer to muscle structure: GO:0061061 muscle structure development (−log10(p)[Adherent undifferentiated vs. Suspension] = −23, −log10(p)[Adherent differentiated vs. Suspension] = −29, −log10(p)[Adherent undifferentiated vs. Differentiated] = −41), GO:0003012 muscle system process (-log10(p)[Adherent undifferentiated vs. Suspension] = −20, −log10(p)[Adherent differentiated vs. Suspension] = −15, −log10(p)[Adherent undifferentiated vs. Differentiated] = −30).

**Figure 3:**
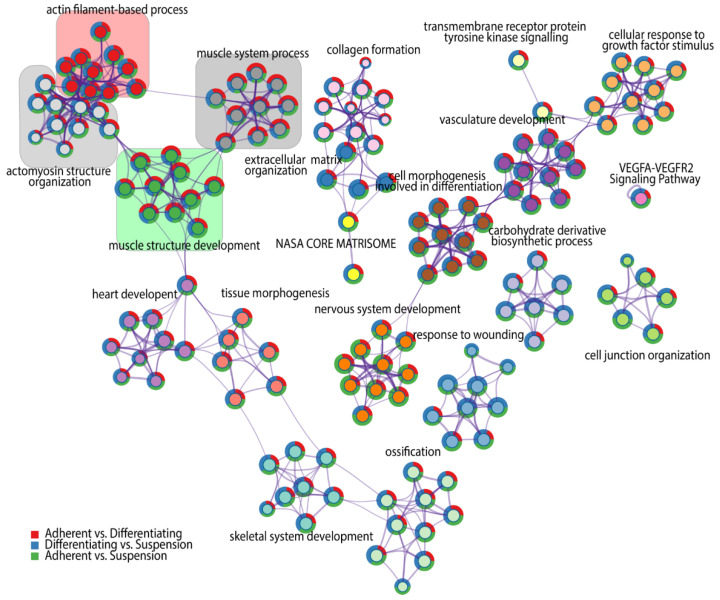
Network of significantly enriched terms obtained by Metascape. Nodes of the network are composed of two circular components. The internal one represents a cluster identified by a generalized name (cluster-ID), coloured by cluster-ID, where nodes sharing the same cluster ID are closer to each other and are marked by this same colour. External components of nodes are colour-coded, corresponding to the DEGs numbers from each comparison.

For each given gene list, a pathway and process enrichment analysis has been carried out, where terms with a *p*-value < 0.01, a minimum count of three and an enrichment factor > 1.5 (the ratio between the observed counts and the counts expected by chance) were grouped into clusters based on their membership similarities. Kappa scores were used as the similarity metric for hierarchical clustering of the enriched terms [30], and subtrees with a similarity of >0.3 are considered a cluster. The analysis showed the 20 independent clusters of different ontology terms, indicated by the respective colours in Figure 3. The superimposed pie chart shows the contribution of genes from each comparison to the formulation of ontology groups belonging to the clusters. We found four clusters that refer to muscle-related ontological terms, marked by coloured squares on the graph. Each of the skeletal muscle-related clusters consists of ten specific ontological terms. The first cluster with a cluster-ID described as “Actin filament-based process” marked by red colour, consists of the following ontological groups: 1—regulation of anatomical structure size, 2—regulation of cytoskeleton organization, 3—supramolecular fiber organization, 4—regulation of actin cytoskeleton organization, 5—actin filament organization, 6—actin cytoskeleton organization, 7—regulation of actin filament organization, 8—regulation of supramolecular fiber organization, 9—regulation of actin filament-based process, 10—actin filament-based process. The second cluster forms a collection of ontological terms that are more specific to the muscle cell cytoskeleton. This cluster is marked by light grey colour and refers to the actomyosin structure organization cluster, including: 1—contractile actin filament bundle assembly, 2—regulation of actomyosin structure organization, 3—regulation of stress fiber assembly, 4— actin filament bundle assembly, 5—actin filament bundle organization, 6—stress fiber assembly, 7—regulation of actin filament bundle assembly, 8—actomyosin structure organization, 9—positive regulation of stress fiber assembly, 10—positive regulation of actin filament bundle assembly. The third cluster, highlighted in green colour, comprises ontological terms related to muscle morphogenesis and includes: 1—myofibril assembly, 2—striated muscle cell development, 3-cellular component assembly involved in morphogenesis, 4—striated muscle cell differentiation, 5—muscle cell development, 6—muscle cell differentiation, 7—muscle tissue development, 8—striated muscle tissue development, 9—muscle organ development, 10-muscle structure development. The fourth cluster, which is marked in dark grey contains ontological terms, including: 1—actin filament-based movement, 2—Muscle contraction, 3—striated muscle contraction, 4—heart process, 5—regulation of heart contraction, 6—muscle contraction, 7—heart contraction, 8—muscle system process, 9—regulation of blood circulation, 10—regulation of system process.

**Figure 4:**
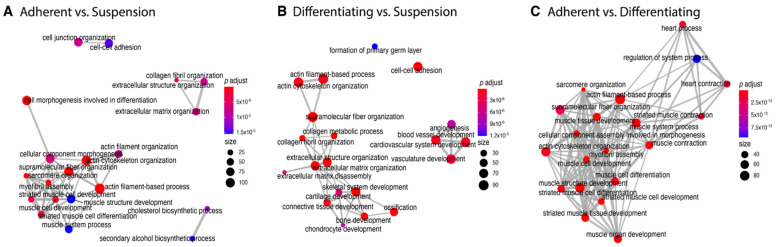
The 20 most statistically significantly enriched ontological terms in the analysed comparisons (**A**–**C**). The colour of nodes corresponds to the *p*-value, and the size of nodes represents the number of genes forming a given ontological group. Graphs were produced using clusterProfiler package.

Alternatively, an enrichment analysis of the gene ontology terms was also carried out using clusterProfiler. The analysis was performed separately for each comparison. Enriched GO BP terms were visualized using an enrichment map—a network where edges connect overlapping gene sets, leading to the identification of common functional ontological clusters (Figure 4). It is worth pointing out that the obtained enrichment of the ontological group corresponds to the previously conducted analysis performed by Metascape.

**Figure 5:**
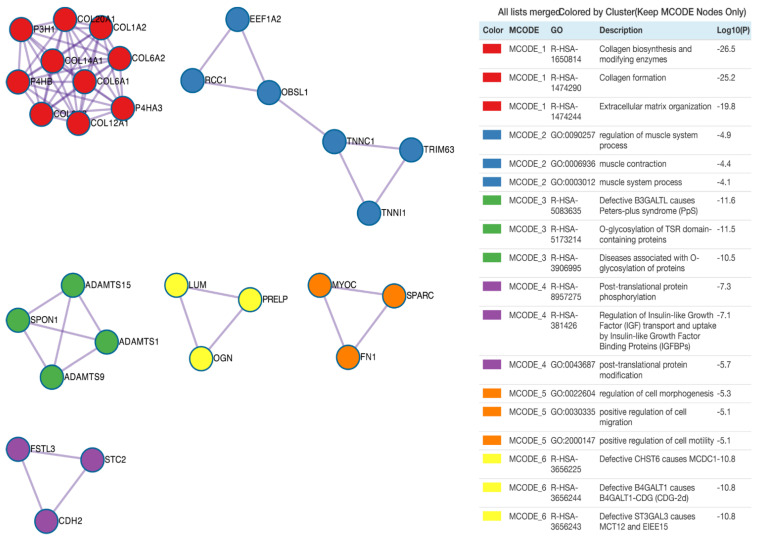
Protein–protein interaction enrichment analysis. Network contains the subset of proteins that form physical interaction with at least one other member from the input list. The Molecular Complex Detection (MCODE) algorithm was applied to identify the density of connected nodules with relevant statistics analysis presented as log10(*p*) value. Each MCODE network is assigned a unique colour.

Subsequently, protein products of differentially expressed genes were evaluated by mutual interactions in the regulation of specific ontological processes. For this reason, Metascape was employed to carry out protein–protein interaction enrichment analysis. Application of the Molecular Complex Detection (MCODE) algorithm allows us to identify seven independent clusters shown in Figure 5, where the three best-scoring terms by *p*-value are presented in the tabular format. The MCODE2 network (coloured blue) refers to the ontological groups related to muscles, including: regulation of muscle system process, muscle contraction and muscle system process. The protein–protein interaction network for the MCODE2 network consists of the following proteins/genes: Eukaryotic Translation Elongation Factor 1 α 2 (*EEF1A2*, RPKM(suspension) = 1528, RPKM(adherent) = 437, RPKM(differentiating) = 178), regulator of chromosome condensation 1 (*RCC1*, RPKM(suspension) = 300, RPKM(adherent) = 123, RPKM(differentiating) = 57), obscurin-like cytoskeletal adaptor 1 (*OBSL1*, RPKM(suspension) = 2, RPKM(adherent) = 220, RPKM(differentiating) = 39), troponin C1, slow skeletal and cardiac type (*TNNC1*, RPKM(suspension) = 23, RPKM(adherent) = 858, RPKM(differentiating) = 197), tripartite motif containing 63 (*TRIM63*, RPKM(suspension) = 578, RPKM(adherent) = 114, RPKM(differentiating) = 34) and troponin I1, slow skeletal type (*TNNI1*, RPKM(suspension) = 72, RPKM(adherent) = 1720, RPKM(differentiating) = 514).

**Figure 6:**
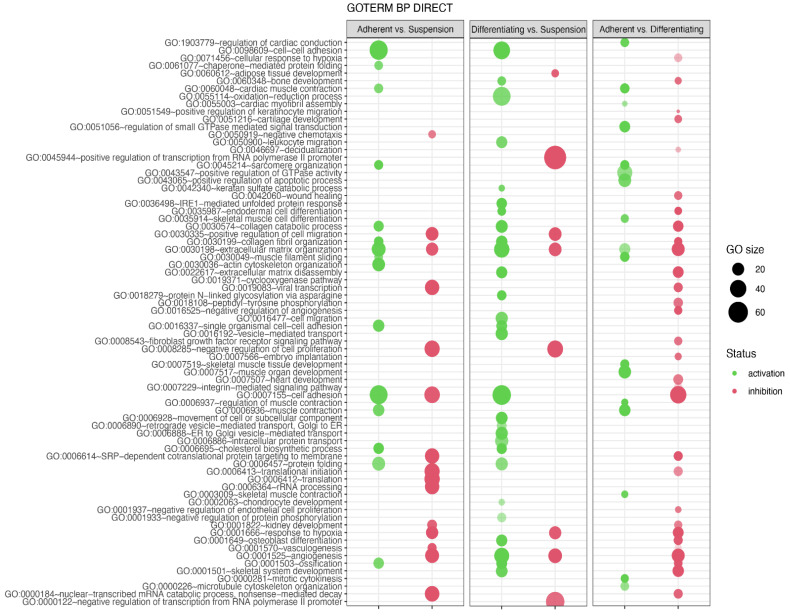
Bubble plot of differentially expressed gene sets overrepresented in the DAVID GO BP DIRECT gene ontology (GO) database. The analysis was performed separately for each experimental group (Adherent vs. Suspension, Differentiating vs. Suspension, Adherent vs. Differentiating). The graph shows only the GO terms above the established cut-off criteria (*p* with correction <0.05 and >5 genes per group). Each bubble’s size reflects the number of differentially expressed genes, assigned to the GO BP terms. The bubbles’ transparency displays *p*-values (more transparent—closer to the *p* = 0.05 cut-off). The red colour indicates downregulated expression of the genes comprising the relevant GO terms; green colour indicates upregulation.

In the next analysis, functional annotation of differentially expressed genes was evaluated using the Database for Annotation, Visualization and Integrated Discovery (DAVID) bioinformatics tool with GO BP Direct database. The relevant GO ontological groups with adjusted *p*-values below 0.05 and N per group > 5 were presented on Figure 6, where, in the comparison Adherent vs. Suspension, we showed that 15 GO BP terms are activated, and 15 terms are inhibited. In these experimental groups, DEGs from the “GO:0006936~muscle contraction” (*N* = 18, *p* = 9.52 × 10^−5^) showed stimulation of expression in adherent cells in relation to suspension cells. Also, genes related to muscle GO terms related to muscle are upregulated in adherent cells compared to suspension cells (“GO:0045214~sarcomere organization”, *N* = 11, *p* = 2.28 × 10^−6^, “GO:0030036~actin cytoskeleton organization”, *N* = 23, *p* = 3.29 × 10^−6^, “GO:0060048~cardiac muscle contraction”, *N* = 11, *p* = 1.60 × 10^−4^, “GO:0030049~muscle filament sliding”, *N* = 10, *p* = 2.02 × 10^−4^). A total of 28 upregulated and 8 downregulated GO BP terms in a comparison of differentiated vs. suspension cells were revealed by the analysis. With regard to muscle-related GO BP terms, it was shown that, in the group of differentiated cells, the genes from “GO:0001501~skeletal system development” term (*N* = 19, *p* = 5.30 × 10^−5^) are stimulated. In the last comparison, i.e., adherent cells vs. differentiated cells, significant inhibition of genes belonging to 29 ontological terms and activation in18 GO PB terms were observed. The expression of genes forming muscle-dependent ontological terms is activated in adherent vs. differentiated cells (“GO:0007517~muscle organ development”, *N* = 22, *p* = 1.48 × 10^−13^, “GO:0045214~sarcomere organization”, *N* = 11, *p* = 6.76 × 10^−9^, “GO:0030049~muscle filament sliding”, *N* = 12, *p* = 9.61 × 10^−9^, “GO:0006936~muscle contraction’, *N* = 18, *p* = 2.02 × 10^−8^, “GO:0060048~cardiac muscle contraction”, *N* = 12, *p* = 6.7 × 10^−08^, “GO:0006937~regulation of muscle contraction”, *N* = 7, *p* = 1.77 × 10^−06^, “GO:0007519~skeletal muscle tissue development”, *N* = 11, *p* = 2.38 × 10^−6^, “GO:1903779~regulation of cardiac conduction”, *N* = 10, *p* = 4.1 × 10^−5^, “GO:0003009~skeletal muscle contraction”, *N* = 7, *p* = 6.16 x10^−5^, “GO:0035914~skeletal muscle cell differentiation”, *N* = 9, *p* = 9.77 × 10^−5^, “GO:0055003~cardiac myofibril assembly”, *N* = 5, *p* = 3.33 × 10^−4^).

**Figure 7:**
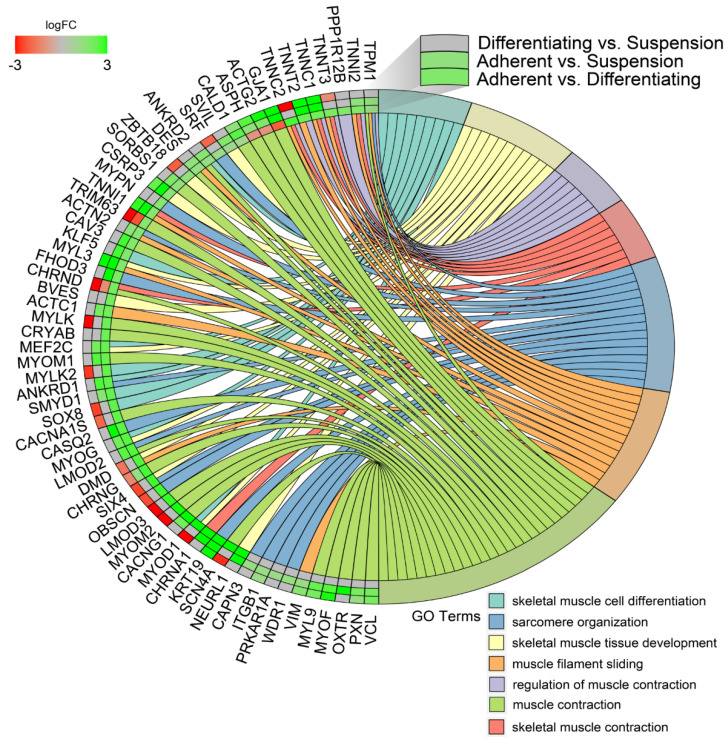
Detailed analysis of seven enriched gene ontology groups selected from the DAVID GO BP DIRECT GO database presented using circos plots. Symbols of DEG from each of the analysed comparisons are displayed on the left side of the graph with their logFC values, mapped by colour scale (green = higher expression; red = lower expression). The grey colour corresponds to expression levels below the cut-off value for the given comparisons. Coloured connecting lines determine gene involvement in the GO terms.

In the next study, changes in the expression profile of specific genes forming previously enriched ontology groups from the DAVID analysis were examined. Assuming that if, in any comparison, a set of genes forming a given ontology group was significantly stimulated or inhibited, then the gene expression profile for that ontology group was presented for all analysed comparisons. The overlaps between the seven muscle-related ontology groups containing genes that were differentially expressed in all of the comparisons were presented using a circos plot (Figure 7). This analysis provided several specific gene expression profiles that changed under different culture conditions. The genes from the analysed groups with the “red, red, green”, colour profile in the circos plots (from the comparisons of Differentiating vs. Suspension, Adherent vs. Suspension, Adherent vs. Differentiating, respectively) concerned only two genes: tripartite motif containing 63 (*TRIM63*) and cholinergic receptor nicotinic delta subunit (*CHRND*), which undergo the highest expression in suspension cells. The next profile, colour-coded in “green, green, green”, concerns genes whose expression is highest in adherent cells, lower in differentiated cells and lowest in suspension cells. The genes mentioned above include: troponin T2 (*TNNT2*), troponin C1 (*TNNC1*), keratin type I cytoskeletal 19-like (*KRT19*), troponin I1 (*TNNI1*) and cysteine and glycine rich protein 3 (*CSRP3*).

Another profile of genes marked in “green, green, red”, referred to genes whose expression is highest in differentiated cells, lower in adherent cells and lowest in suspension cells. This group consists of: aspartate β-hydroxylase (*ASPH*), actin, γ-enteric smooth muscle (*ACTG2*) and gap junction protein α 1 (*GJA1*).

**Figure 8:**
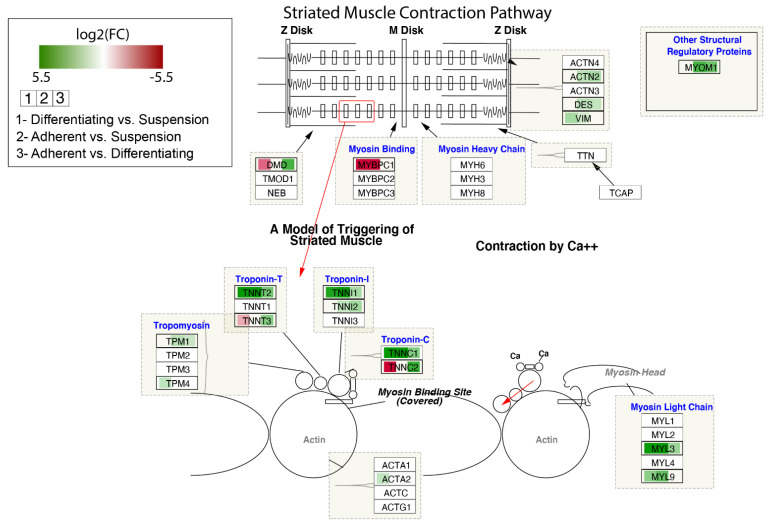
Striated Muscle Contraction Pathway. Colours map expression changes of genes; green colour—statistically significant increase in expression, red colour—statistically significant decrease in expression, white colour—statistically insignificant. The box in the pathway that corresponds to a given gene was divided into three parts, coloured separately according to the gene expression comparisons. Graph was produced using Cytoscape (v. 3.7.2) with RCy3 library.

The contribution of DEGs in the modulation of the Striated Muscle Contraction Pathway is shown in Figure 8. The log2 fold-change values of DEGs were mapped with appropriate colours. Agreen colour indicates statistically significant upregulated genes, and a red colour refers to downregulated genes. A white colour marks genes whose expression was not significantly changed. As we noticed previously for some genes forming troponin-T, troponin-I and troponin-C, the highest expression was observed in adherent cells, lower in differentiated cells and the lowest in suspend cells (*TNNT2*, *TNNI1*, *TNNC1*).

The differential expression analysis yielded lists of 1673 DEGs between Adherent vs. Suspension, 876 DEGs between Adherent and Differentiating and 1594 DEGs between Differentiating and Suspension, which were used for Ingenuity Pathway Analysis. Core analysis was performed and figures for the top three directionally enriched canonical pathways for each comparison were generated. The top enriched pathways between Adherent vs. Suspension were EIF2 signalling and mTOR signaling, which were inhibited, and leukocyte extravasation signaling, which was activated (Figures S1–S3).

For Adherent vs. Differentiating, calcium signalling, GP6 signalling and inhibition of matrix metalloproteases were the top three enriched pathways and were all activated (Figures S4–S6).

Integrin signalling, actin cytoskeletal signalling and Hifα signalling were the top enriched pathways in Differentiating vs. Suspension and were all activated (Figures S7–S9).

## 4. Discussion

Modern molecular analysis techniques allow researchers to conduct complete transcriptomic analysis, tracking the changes in expression of genes over the durtaion of long-term in vitro culture and differentiation [31]. In this study, we have investigated turkey muscle satellite cells, extracted from the *Pectoralis thoracicus* muscle of a 1-day old specimen, to study the molecular influence of an Adherent vs. Suspension environment on their long-term culture. Furthermore, myotube differentiation of the adherent cells was performed to provide a further point of control reference for the analysis of suspension-cultured cells. This approach should provide information on whether suspension cultures, indicated as one of the best approaches for potential large-scale in vitro meat production, significantly influence the levels of gene expression in myosatellites [3]. In turn, the advanced bioinformatical analysis of the differentially expressed genes provides a molecular insight into the processes occurring during the long term culture of muscle satellite cells, potentially elucidating the influence of the different ex vivo conditions on their functioning and potential differentiation [12].

It is well known that culture conditions can influence gene expression of the cultured cells, with the initial results of the study confirming these notions [32]. Interestingly, gene ontology analysis indicated that the genes belonging to muscle-associated terms were generally upregulated in adherent vs. suspension cultures. Similar dependencies were observed in differentiated vs. suspension, as well as adherent vs. differentiated comparisons. These changes suggest that the suspension-cultured cells are characterised by a significantly larger deviation from the muscle phenotype. However, as the suspension-cultured cells were also characterised by a lower expression of muscle-specific genes, the results of the study confirm the previous reports that ex vivo conditions promote culture stem cells’ plasticity, with the long-term in vitro culture predisposing them to a phenotype different than physiological [33].

Moreover, the downstream analysis of the results focused on signalling pathways that were differentially regulated among the analysed sample groups. Firstly, in the adherent vs. suspension analysis, the top enriched pathways included EIF2 and mTor signalling (inhibited) and leukocyte extravation signalling (activated). EIF2 signalling is a pathway governing the initiation of translation, with its action essential for the proper course of this process [34]. In the context of stem cells, Jeske et al. connected the downregulation of EIF2 signalling to the change in immune phenotype in in vitro cultured mesenchymal stem cells (MSCs) [35]. Furthermore, Bijonowski et al. connected the activity of the EIF2 pathway to MSC stemness, with its inhibition significantly impairing stem cell plasticity [36]. In turn, this pathway was also implicated in cancer, due to the exceptional translational activity of the tumours [37]. The mTOR pathway normally acts as an intermediate, integrating upstream and downstream pathways, and was previously associated with the build-up of muscle mass [38]. Bodine et al., in an in vivo study, connected mTOR activity to increased muscle mass in the analysed subjects, as well as adaptation to resistance training [39]. Moreover, mTOR signalling was implicated in the maintenance of stem cell function during aging, as well as mediating their differentiation [40]. Additionally, mTOR signalling was also associated with cancer, by way of increased pathway activity characteristic for malignant tumours [37]. In this comparison, the results suggest that adherent cells exhibit significantly less plasticity than those cultured in suspension, as the activity of both EIF2 and mTOR pathways were connected to increased proliferation and differentiation of MSCs. The role of the leukocyte extravasation pathway activation is not clear, but it is possible that it occurs due to the major participation of both of the aforementioned pathways in its mechanisms. However, it is also worth noting that all of these pathways were implicated in regulation of the innate inflammatory response [41,42]. Hence, it cannot be ruled out that their regulation could be associated with the environmental stress to which the analysed cells were subjected in the suspended culture conditions. Nonetheless, further studies would be needed to fully elucidate the mechanism and effect of their potential participation in such response.

In the adherent vs. differentiating myosatellites comparison, all three of the most enriched pathways, calcium signalling, GP6 signalling and inhibition of matrix metalloproteinases, were activated. Calcium signalling is well known to participate in the processes associated with muscle function and development [43,44,45]. It also plays a role in the activation of satellite cells for proliferation and differentiation [46]. Furthermore, it has been implicated in a range of processes associated with MSC function and progression towards different lineages [47]. Most probably, the GP6 pathway, which has not been previously associated with any muscle, or stem cell-associated processes, has been significantly enriched due to the large overlap with the aforementioned calcium signalling pathway. In turn, activation of the matrix metalloproteinase inhibition pathway suggests significantly higher inhibition of these proteins in adherent vs. differentiating cells. Matrix metalloproteinases (MMPs) have been implicated in a range of muscle cell-associated process both in vivo and in vitro, including their proliferation, migration and differentiation [48]. Furthermore, MMPs were reported to be involved in the activation of satellite cells induced by stretching [49], while their inhibition was shown to supress muscle cell migration [50]. In the context of stem cells in general, MMPs were reported to mediate their proliferation and plasticity, both in physiology and cancer [51,52,53]. In summary, the activation of calcium signalling and GP6 pathways in adherent vs. differentiating cells points, unsurprisingly, is associated with the larger plasticity and potential of the undifferentiated long-term in vitro-cultured cells. Moreover, the activation of pathways associated with MMP inhibition suggests lower migration abilities of the undifferentiated cells. However, the results are somehow contrary, therefore yielding this part of the analysis somewhat inconclusive.

In the final comparison of differentiating vs. suspension-cultured cells, the three most enriched pathways (integrin signalling, actin cytoskeletal signalling and HIFα signalling) were all activated. Integrin signalling has been associated with the process of muscle function, with its inhibition in satellite cells resulting in impaired muscle regeneration [54]. Furthermore, it is also connected to the topic of stem cells, with integrins indicated as major players in the extracellular matrix-associated stem cells’ processes [55]. Signalling pathways involving integrins have also been described as crucial for the formation of the stem cell niche [56]. When it comes to the signalling associated with actin cytoskeleton, this structure mostly plays a role in smooth muscle cell development [57]. Nevertheless, diverse roles of actin cytoskeleton in striated muscles have also been indicated [58]. Furthermore, changes and regulation in this structure have been proven to play a major role in smooth muscle cell migration [59]. Furthermore, HIFα was found to play a role in muscle metabolic changes induced by hypoxia [60]. Modulation of HIF was described as regulating skeletal myogenesis in vivo through the modulation of Wnt signalling [61]. Moreover, HIF-hypoxia signalling was implicated in the physiological functioning of the striated muscle, especially in its regeneration and homeostasis, as well as in the process of muscle fibrosis [62,63]. In summary, the most differentially regulated pathways between differentiating and suspension-cultured cells further suggest a loss of muscle cell characteristics associated with suspension cultures, which is made especially apparent due to the comparison with differentiating myoblasts, resembling those observed in in vivo experiments.

## 5. Conclusions

Overall, the results of the study provide a molecular insight into the effects of suspension culture on myosatellite cells. The differential regulation of gene expression, as well as significant enrichment and modulation of pathway activity, suggest that suspension culture potentially promotes the ex vivo-associated loss of physiological characteristics and gain of plasticity. These differences are especially pronounced in comparison with differentiating myoblasts. Therefore, it seems that suspension cultures, often considered the desired method for in vitro meat production, require further investigation to fully elucidate their effect on myosatellites and possibly enable their easier scalability to ensure suitability for industrial application. However, it needs to be noted that the obtained results come from a purely transcriptomic analysis. Hence, while the obtained information about the intrinsic molecular mechanisms regarding myosatellite cells in suspension culture is a valuable point of reference for future studies, it needs to be further validated on a proteomic level.

## Figures and Tables

**Table 1:** Detailed list of the top 20 most regulated genes (ten up and ten down) from the following comparisons: adherent vs. suspension, adherent differentiated vs. suspension, adherent undifferentiated vs. differentiating. Relevant gene symbols’ gene names, RPKM values of two compared groups, “M” value corresponding to the log2 ratio between two compared conditions (log2(RPKM [first group]/RPKM [second group])), “D” the value difference between conditions (RPKM [group with higher RPKM]—RPKM[group with lower RPKM]) and *p*-values are shown.

Adherent vs. Suspension						
Symbol	Description	RPKM (Adherent)	RPKM (Suspension)	M	D	*p*-Value
*CACNG3*	calcium voltage-gated channel auxiliary subunit γ 3	93.74	0.07	10.40	93.67	0.0004
*WNT10A*	Wnt family member 10A	22.51	0.03	9.34	22.48	0.01
*ADAM8*	ADAM metallopeptidase domain 8	215.70	0.49	8.79	215.21	0.00003
*CHST1*	carbohydrate sulfotransferase 1	15.41	0.03	8.79	15.37	0.03
*MYL3*	myosin light chain 3	932.39	3.61	8.01	928.79	0.0000001
*GJD4*	gap junction protein delta 4	34.45	0.21	7.37	34.24	0.005
*NMU*	neuromedin U	32.12	0.21	7.27	31.91	0.006
*OBSL1*	obscurin like cytoskeletal adaptor 1	219.66	1.53	7.17	218.13	0.00003
*WNT7A*	Wnt family member 7A	17.79	0.14	7.00	17.66	0.02
*MMP27*	matrix metallopeptidase 27	79.04	0.62	6.98	78.42	0.0007
*IFITM5*	interferon induced transmembrane protein 5	0.16	117.07	−9.49	116.90	0.0002
*SPON2*	spondin 2	0.54	426.83	−9.62	426.29	0.0000001
*CDH19*	cadherin 19	0.03	24.56	−9.82	24.54	0.01
*DPT*	dermatopontin	0.05	51.28	−9.88	51.23	0.002
*SNCA*	synuclein α	0.03	29.08	−10.07	29.05	0.008
*CHODL*	chondrolectin	0.03	31.02	−10.16	30.99	0.007
*SCARA3*	scavenger receptor class A member 3	0.03	36.43	−10.39	36.40	0.004
*PLP1*	proteolipid protein 1	0.05	75.57	−10.44	75.51	0.0007
*C1QTNF2*	C1q and TNF related 2	0.05	75.98	−10.45	75.93	0.0007
*FGL2*	fibrinogen like 2	0.27	397.62	−10.52	397.35	0.0000001
Differentiating vs. Suspension						
*CACNG3*	calcium voltage-gated channel auxiliary subunit γ 3	77.22	0.07	10.12	77.15	0.0008
*ADAM8*	ADAM metallopeptidase domain 8	455.09	0.49	9.87	454.61	0.0000001
*MMP13*	matrix metallopeptidase 13	64.49	0.14	8.86	64.35	0.001
*EPHX4*	epoxide hydrolase 4	13.12	0.07	7.56	13.05	0.04
*RSPO4*	R-spondin 4	23.03	0.14	7.37	22.89	0.01
*MMP27*	matrix metallopeptidase 27	90.79	0.62	7.18	90.16	0.0005
*ACAN*	agg recan	92.72	0.69	7.06	92.03	0.0005
*PHGDH*	phosphoglycerate dehydrogenase	980.38	8.54	6.84	971.84	0.0000001
*PRRX2*	paired related homeobox 2	101.80	1.18	6.43	100.62	0.0004
*PGF*	placental growth factor	227.74	2.91	6.29	224.83	0.00003
*MMRN2*	multimerin 2	0.06	28.66	−9.02	28.60	0.008
*SNCA*	synuclein α	0.06	29.08	−9.04	29.02	0.008
*MATN1*	matrilin 1	0.03	17.21	−9.28	17.18	0.02
*RGS5*	regulator of G-protein signaling 5	0.11	68.98	−9.28	68.87	0.001
*BCAS1*	breast carcinoma amplified sequence 1	0.17	104.16	−9.29	103.99	0.0004
*GJA4*	gap junction protein α 4	0.03	17.63	−9.31	17.60	0.02
*CNTN1*	contactin 1	0.06	38.58	−9.44	38.53	0.004
*FOXJ1*	forkhead box J1	0.03	27.48	−9.96	27.45	0.009
*FGL2*	fibrinogen like 2	0.22	397.63	−10.81	397.41	0.0000001
*PLP1*	proteolipid protein 1	0.03	75.57	−11.41	75.54	0.0009
Adherent vs. Differentiating						
*OPCML*	opioid binding protein/cell adhesion molecule like	14.11	0.50	4.82	13.61	0.04
*FAT3*	FAT atypical cadherin 3	27.89	1.11	4.65	26.78	0.008
*PDGFB*	platelet derived growth factor subunit B	26.21	1.11	4.56	25.10	0.01
*OPTC*	opticin	34.67	1.66	4.38	33.01	0.005
*SLIT1*	slit guidance ligand 1	504.98	28.24	4.16	476.74	0.00006
*PLXNA2*	plexin A2	15.03	0.89	4.08	14.15	0.03
*PAX7*	paired box 7	84.54	5.26	4.01	79.28	0.0007
*PTPRQ*	protein tyrosine phosphatase receptor type Q	80.53	5.04	4.00	75.49	0.0008
*NHSL2*	NHS like 2	53.83	3.88	3.80	49.95	0.002
*AIF1L*	allograft inflammatory factor 1 like	84.71	6.59	3.68	78.12	0.0008
*SCIN*	scinderin	0.11	24.97	−7.85	24.86	0.01
*GFPT2*	glutamine-fructose-6-phosphate transaminase 2	0.38	105.42	−8.12	105.04	0.0002
*SLC1A6*	solute carrier family 1 member 6	0.05	16.67	−8.26	16.61	0.02
*TTC29*	tetratricopeptide repeat domain 29	0.16	67.94	−8.71	67.77	0.0009
*SPON2*	spondin 2	0.54	377.28	−9.44	376.74	0.0000001
*OGN*	osteoglycin	0.43	546.16	−10.30	545.72	0.0000001
*AKR1D1*	aldo-keto reductase family 1 member D1	0.38	480.65	−10.31	480.27	0.0000001
*DPT*	dermatopontin	0.05	90.36	−10.70	90.31	0.0003
*ACAN*	aggrecan	0.05	92.74	−10.74	92.69	0.0003
*SCARA3*	scavenger receptor class A member 3	0.03	101.21	−11.87	101.19	0.0002

## Data Availability

All data described in this manuscript, if not already a part of its contents and/or Supplementary Materials, is available from the corresponding authors upon reasonable request.

## Supplementary Materials

The following are available online at https://www.mdpi.com/article/10.3390/genes13020242/s1, Figure S1: Adherent vs. suspension EIF2 signaling pathway. Graph was produced using Ingenuity Pathway Analysis (IPA, Qiagen, Hilden Germany), Figure S2: Adherent vs. suspension mTor signaling pathway. Graph was produced using Ingenuity Pathway Analysis (IPA, Qiagen, Hilden Germany), Figure S3: Adherent vs. suspension leukocyte extravation signaling pathway. Graph was produced using Ingenuity Pathway Analysis (IPA, Qiagen, Hilden Germany), Figure S4: Adherent vs. differentiating calcium signaling pathway. Graph was produced using Ingenuity Pathway Analysis (IPA, Qiagen, Hilden Germany), Figure S5: Adherent vs. differentiating GP6 signaling pathway. Graph was produced using Ingenuity Pathway Analysis (IPA, Qiagen, Hilden Germany), Figure S6: Adherent vs. differentiating Inhibition of matrix metalloproteases pathway. Graph was produced using Ingenuity Pathway Analysis (IPA, Qiagen, Hilden Germany), Figure S7: Differentiating vs. suspension integrin signaling pathway. Graph was produced using Ingenuity Pathway Analysis (IPA, Qiagen, Hilden Germany), Figure S8: Differentiating vs. suspension actin cytoskeletal signaling pathway. Graph was produced using Ingenuity Pathway Analysis (IPA, Qiagen, Hilden Germany), Figure S9: Differentiating vs. suspension Hifα signalling pathway. Graph was produced using Ingenuity Pathway Analysis (IPA, Qiagen, Hilden Germany), Table S1: The list of all differentially expressed genes detected in this study.
