# Supplementary material for: Gene Ontology Groups and Signaling Pathways Regulating the Process of Avian Satellite Cell Differentiation
Source: Genes (Basel). 2022 Jan 27;13(2):242. doi: 10.3390/genes13020242 (PMC8871586; doi:10.3390/genes13020242)
Supplement: Supplementary file 1 [file genes-13-00242-s001.zip › genes-1446616-supplementary.pdf]

genes

MDPI

Supplementary Materials

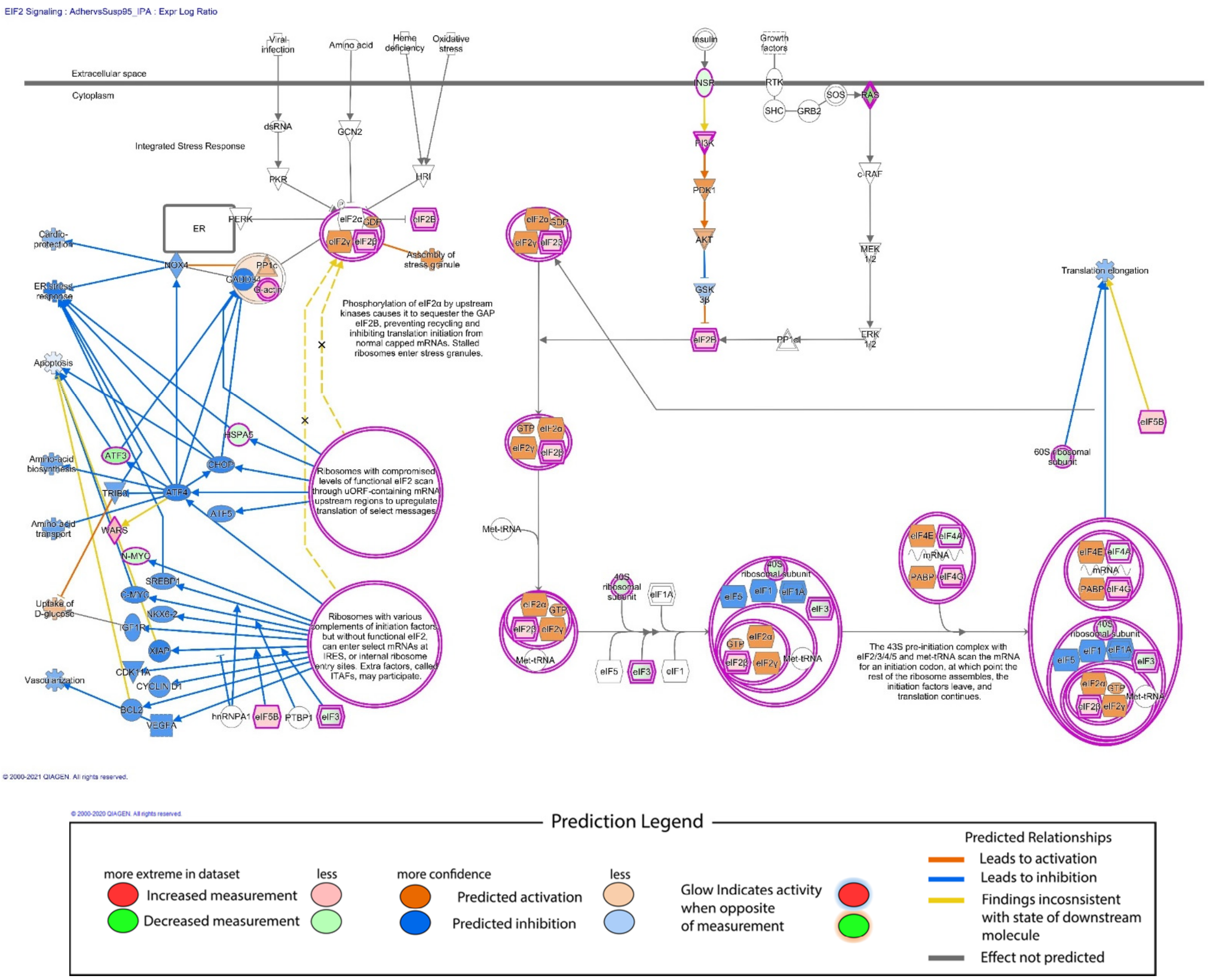

Figure S1. Adherent vs. suspension EIF2 signaling pathway. Graph was produced using Ingenuity Pathway Analysis (IPA, Qiagen, Hilden Germany).

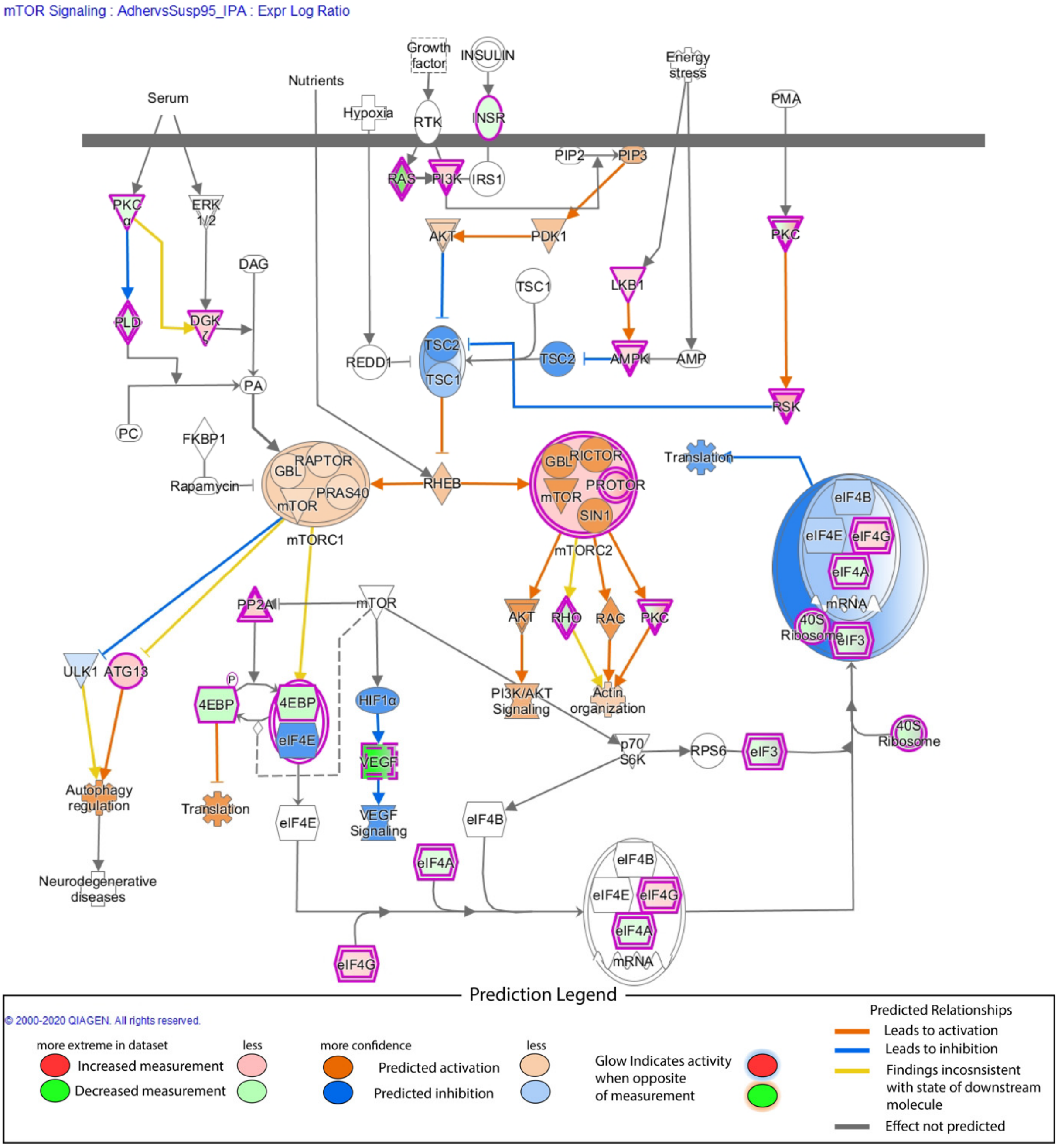

Figure S2. Adherent vs. suspension mTor signaling pathway. Graph was produced using Ingenuity Pathway Analysis (IPA, Qiagen, Hilden Germany).

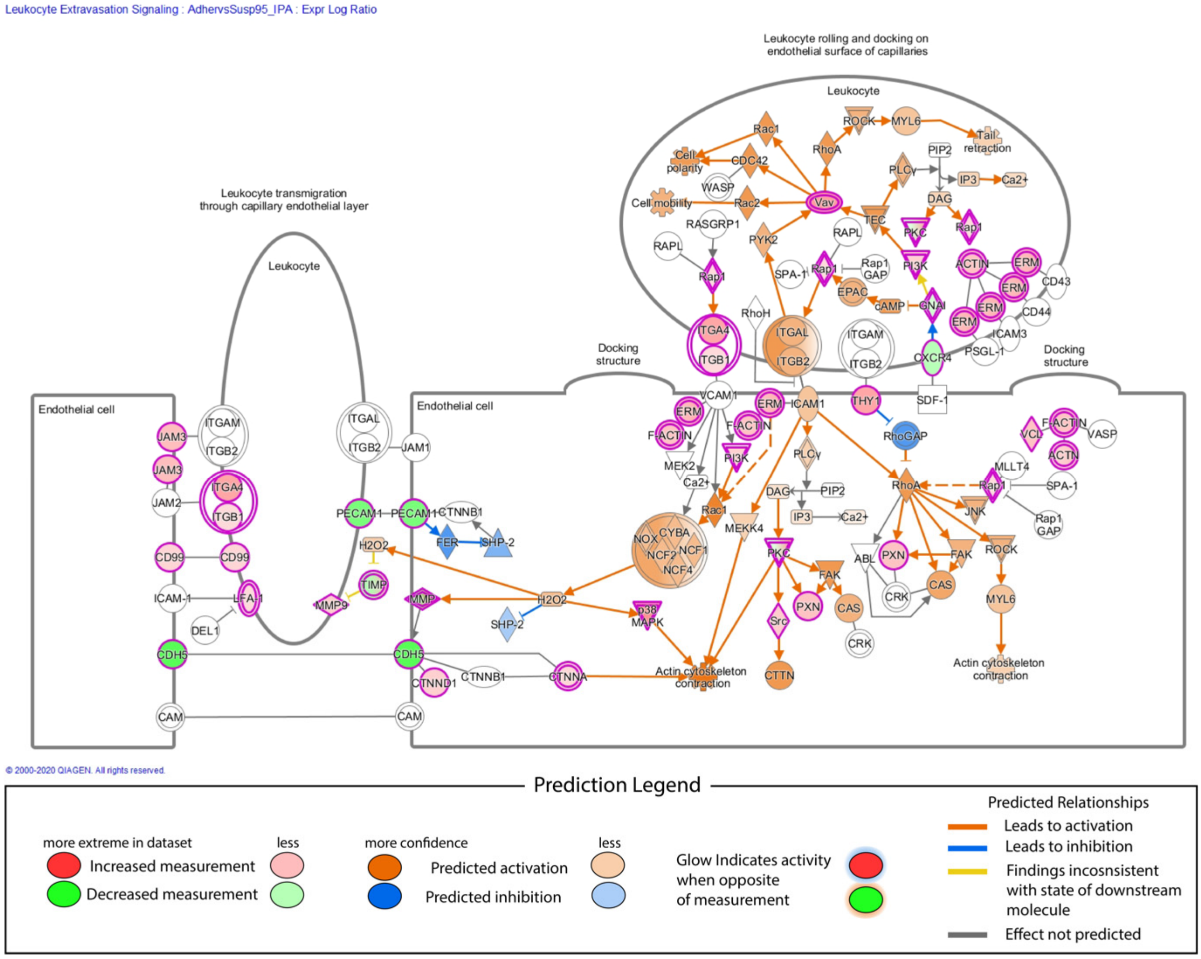

Figure S3. Adherent vs. suspension leukocyte extravation signaling pathway. Graph was produced using Ingenuity Pathway Analysis (IPA, Qiagen, Hilden Germany).

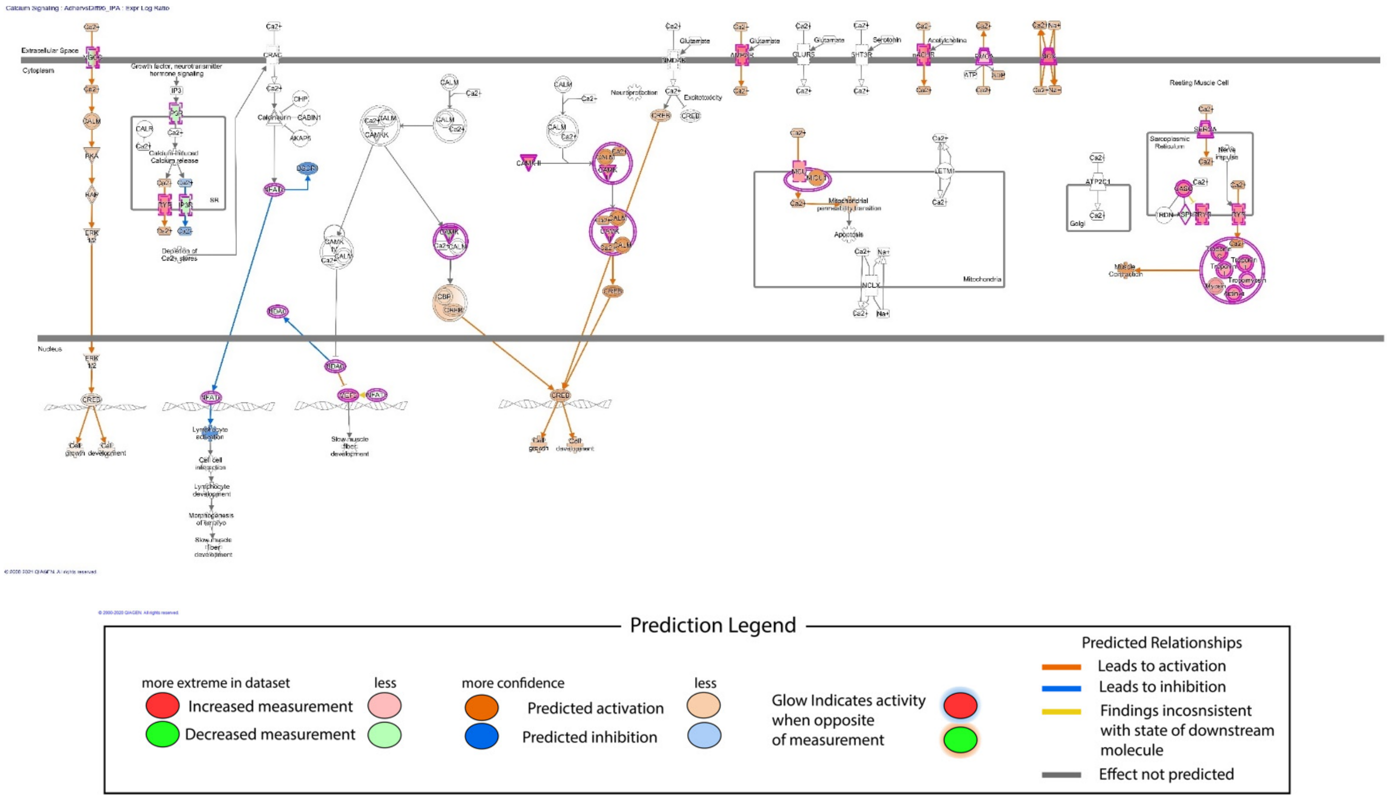

Figure S4. Adherent vs. differentiating calcium signaling pathway. Graph was produced using Ingenuity Pathway Analysis (IPA, Qiagen, Hilden Germany).

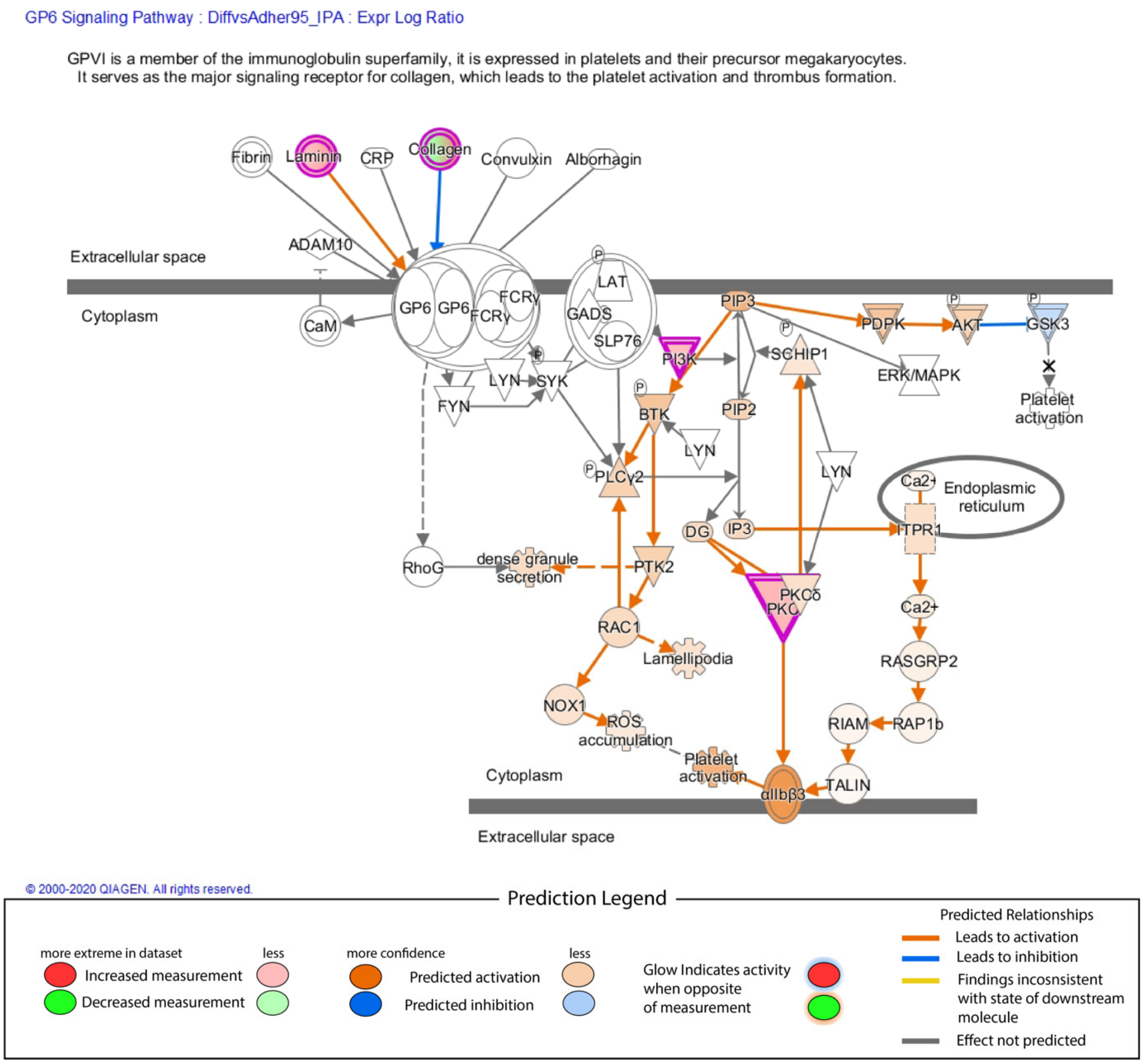

Figure S5. Adherent vs. differentiating GP6 signaling pathway. Graph was produced using Ingenuity Pathway Analysis (IPA, Qiagen, Hilden Germany).

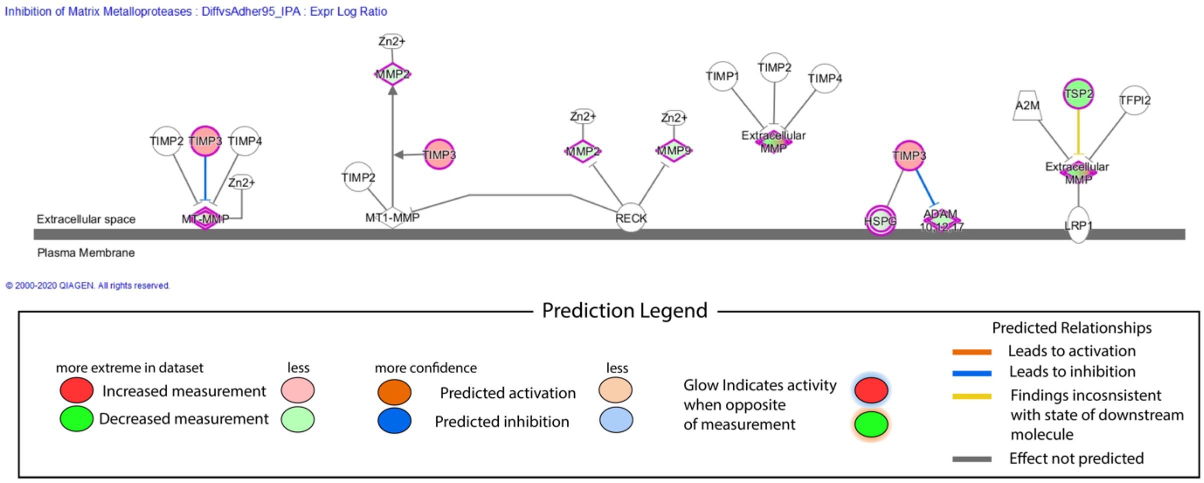

Figure S6. Adherent vs. differentiating Inhibition of matrix metalloproteases pathway. Graph was produced using Ingenuity Pathway Analysis (IPA, Qiagen, Hilden Germany).

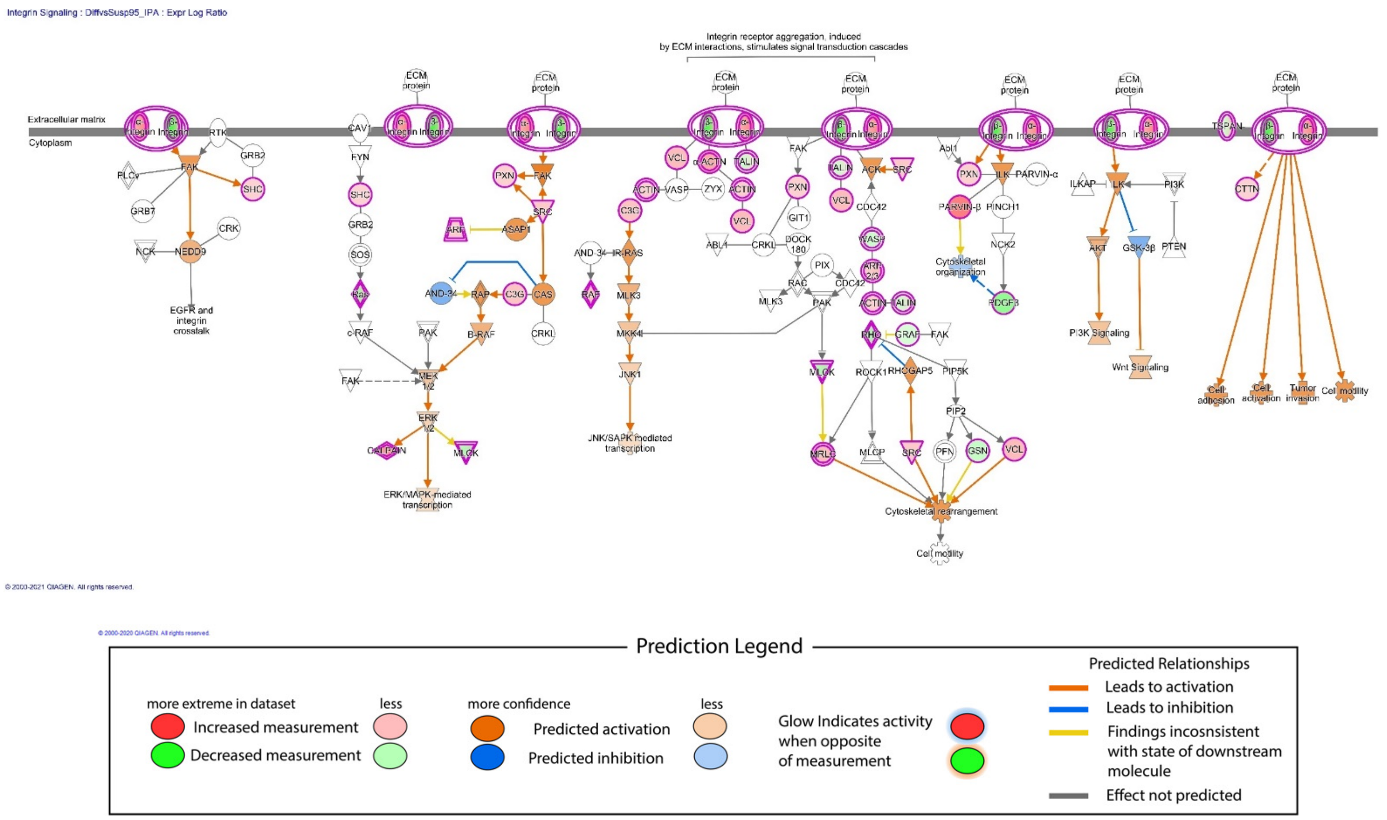

Figure S7. Differentiating vs. suspension integrin signaling pathway. Graph was produced using Ingenuity Pathway Analysis (IPA, Qiagen, Hilden Germany).

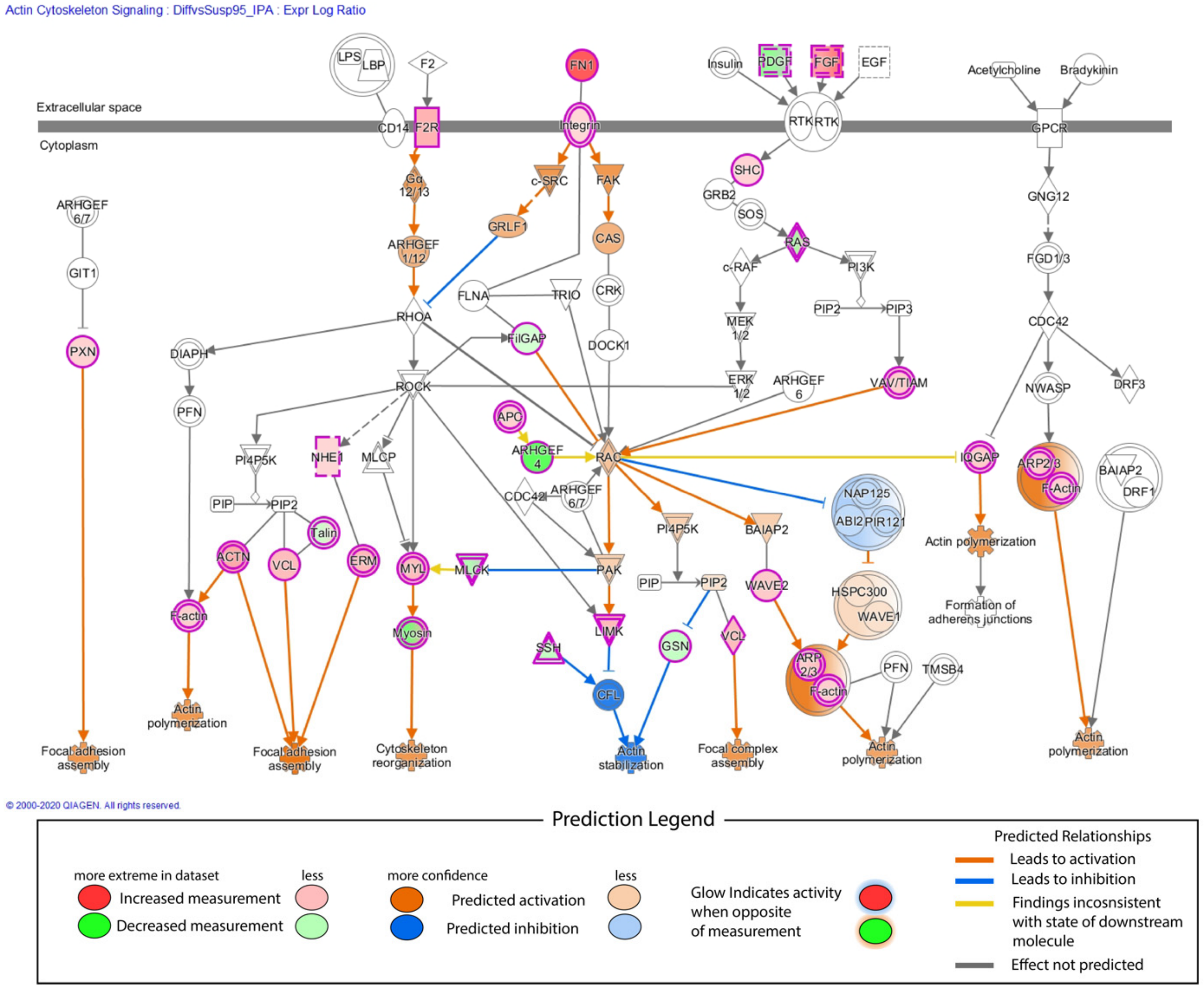

Figure S8. Differentiating vs. suspension actin cytoskeletal signaling pathway. Graph was produced using Ingenuity Pathway Analysis (IPA, Qiagen, Hilden Germany).

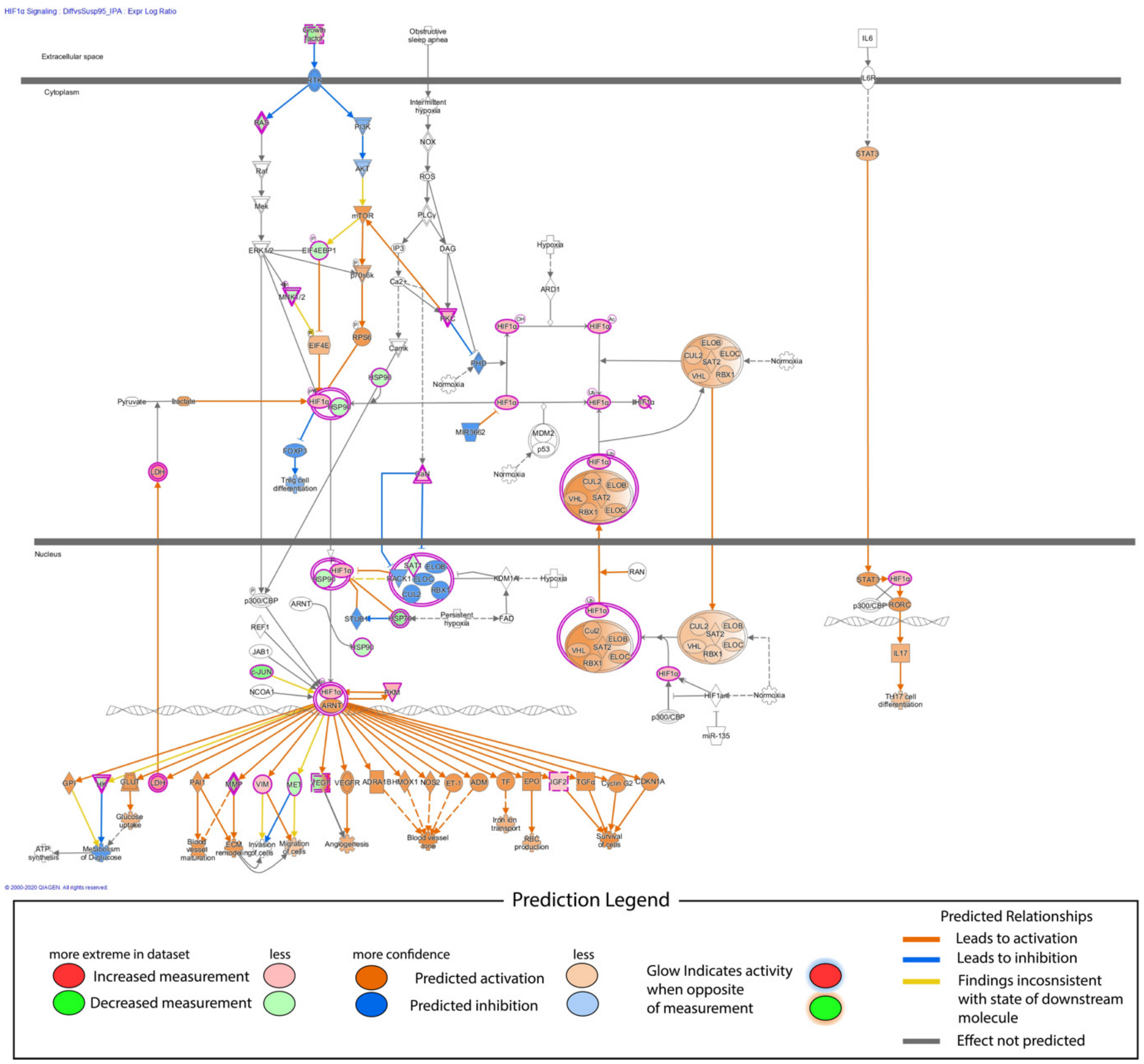

Figure S9. Differentiating vs. suspension Hifα signalling pathway. Graph was produced using Ingenuity Pathway Analysis (IPA, Qiagen, Hilden Germany).

Table S1. The list of all differentially expressed genes detected in this study.

Symbol	Description	RPKM (Adherent)	RPKM (Differentiating)	M	D	p-value
ABCB6	ATP binding cassette subfamily B member 6 (Langereis blood group) [Source:HGNC Symbol;Acc:HGNC:47]	48,51	22,65	1,10	25,87	0,05
ABLIM3	actin binding LIM protein family member 3 [Source:HGNC Symbol;Acc:HGNC:29132]	319,29	72,87	2,13	246,42	0,01
ABRA	actin binding Rho activating protein [Source:HGNC Symbol;Acc:HGNC:30655]	24,85	4,71	2,40	20,15	0,02
ABRACL	ABRA C-terminal like [Source:NCBI gene;Acc:100549256]	12,53	32,67	-1,38	20,13	0,04
ACAA2	acetyl-CoA acyltransferase 2 [Source:HGNC Symbol;Acc:HGNC:83]	279,95	87,76	1,67	192,19	0,02
ACAN	aggrecan [Source:HGNC Symbol;Acc:HGNC:319]	0,05	92,74	-10,74	92,69	0,00
ACAP3	ArfGAP with coiled-coil, ankyrin repeat and PH domains 3 [Source:HGNC Symbol;Acc:HGNC:16754]	5,48	20,32	-1,89	14,84	0,05
ACKR3	atypical chemokine receptor 3 [Source:NCBI gene;Acc:100546352]	2,22	162,56	-6,19	160,34	0,00
ACSL1	acyl-CoA synthetase long chain family member 1 [Source:NCBI gene;Acc:100546186]	58,44	138,53	-1,25	80,09	0,03
ACTC1	actin, alpha cardiac muscle 1 [Source:NCBI gene;Acc:100303693]	10717,90	2498,12	2,10	8219,78	0,01
ACTG2	actin, gamma-enteric smooth muscle [Source:NCBI gene;Acc:100551088]	833,76	1724,45	-1,05	890,70	0,04
ACTN2	actinin alpha 2 [Source:HGNC Symbol;Acc:HGNC:164]	1443,73	428,39	1,75	1015,35	0,02

ACVRL1	activin A receptor like type 1 [Source:HGNC Symbol;Acc:HGNC:175]	1,36	33,33	-4,62	31,98	0,01
ADAM12	ADAM metallopeptidase domain 12 [Source:HGNC Symbol;Acc:HGNC:190]	14,81	128,90	-3,12	114,08	0,00
ADAM33	ADAM metallopeptidase domain 33 [Source:HGNC Symbol;Acc:HGNC:15478]	165,23	68,10	1,28	97,13	0,03
ADAM8	ADAM metallopeptidase domain 8 [Source:HGNC Symbol;Acc:HGNC:215]	215,75	455,19	-1,08	239,43	0,04
ADAMTS1	ADAM metallopeptidase with thrombospondin type 1 motif 1 [Source:HGNC Symbol;Acc:HGNC:217]	20,40	68,82	-1,75	48,42	0,02
ADAMTS15	ADAM metallopeptidase with thrombospondin type 1 motif 15 [Source:HGNC Symbol;Acc:HGNC:16305]	0,60	96,73	-7,34	96,13	0,00
ADAMTS2	ADAM metallopeptidase with thrombospondin type 1 motif 2 [Source:HGNC Symbol;Acc:HGNC:218]	14,11	39,59	-1,49	25,48	0,03
ADAMTS9	ADAM metallopeptidase with thrombospondin type 1 motif 9 [Source:HGNC Symbol;Acc:HGNC:13202]	2,39	22,76	-3,25	20,37	0,02
ADCY2	adenylate cyclase 2 [Source:HGNC Symbol;Acc:HGNC:233]	49,65	18,55	1,42	31,10	0,03
ADCY9	adenylate cyclase 9 [Source:HGNC Symbol;Acc:HGNC:240]	619,09	200,27	1,63	418,82	0,02
ADD3	adducin 3 [Source:NCBI gene;Acc:100547330]	23,93	63,51	-1,41	39,58	0,03
ADGRD2	adhesion G protein-coupled receptor D2 [Source:HGNC Symbol;Acc:HGNC:18651]	85,09	7,92	3,43	77,17	0,00
ADGRG1	adhesion G protein-coupled receptor G1 [Source:HGNC Symbol;Acc:HGNC:4512]	200,18	32,17	2,64	168,01	0,00
ADK	adenosine kinase [Source:HGNC Symbol;Acc:HGNC:257]	136,26	26,69	2,35	109,57	0,01
ADORA1	adenosine A1 receptor [Source:HGNC Symbol;Acc:HGNC:262]	131,21	23,25	2,50	107,95	0,01
ADSS2	adenylosuccinate synthase 2 [Source:HGNC Symbol;Acc:HGNC:292]	32,56	81,34	-1,32	48,78	0,03
AFAP1L1	actin filament associated protein 1 like 1 [Source:HGNC Symbol;Acc:HGNC:26714]	370,67	66,66	2,48	304,01	0,01
AFDN	afadin, adherens junction formation factor [Source:HGNC Symbol;Acc:HGNC:7137]	169,41	50,66	1,74	118,75	0,02
AGL	amylo-alpha-1, 6-glucosidase, 4-alpha-glucanotransferase [Source:HGNC Symbol;Acc:HGNC:321]	101,64	32,45	1,65	69,19	0,02
AGRN	agrin [Source:HGNC Symbol;Acc:HGNC:329]	16,55	1,44	3,52	15,11	0,03
AHCY	adenosylhomocysteinase [Source:HGNC Symbol;Acc:HGNC:343]	976,09	466,76	1,06	509,33	0,04
AIF1L	allograft inflammatory factor 1 like [Source:HGNC Symbol;Acc:HGNC:28904]	84,71	6,59	3,68	78,12	0,00
AK4	adenylate kinase 4 [Source:NCBI gene;Acc:100538743]	35,92	88,15	-1,30	52,22	0,03
AKAP12	A-kinase anchoring protein 12 [Source:HGNC Symbol;Acc:HGNC:370]	146,29	25,30	2,53	120,99	0,00
AKAP13	A-kinase anchoring protein 13 [Source:HGNC Symbol;Acc:HGNC:371]	57,30	25,08	1,19	32,22	0,04
AKR1D1	aldo-keto reductase family 1 member D1 [Source:NCBI gene;Acc:100547967]	0,38	480,65	-10,31	480,27	0,00
ALAS1	5'-aminolevulinate synthase 1 [Source:NCBI gene;Acc:100547950]	1264,34	453,91	1,48	810,43	0,02
ALDH1A3	aldehyde dehydrogenase 1 family member A3 [Source:HGNC Symbol;Acc:HGNC:409]	5,86	47,28	-3,01	41,42	0,00
ALDH4A1	aldehyde dehydrogenase 4 family member A1 [Source:HGNC Symbol;Acc:HGNC:406]	84,92	27,63	1,62	57,29	0,02
ALDH7A1	aldehyde dehydrogenase 7 family member A1 [Source:HGNC Symbol;Acc:HGNC:877]	78,57	35,77	1,14	42,81	0,04
ALPK3	alpha kinase 3 [Source:HGNC Symbol;Acc:HGNC:17574]	148,41	25,30	2,55	123,11	0,00
ALPL	alkaline phosphatase, biomineralization associated [Source:HGNC Symbol;Acc:HGNC:438]	7,76	120,65	-3,96	112,89	0,00
AMOTL2	angiomotin like 2 [Source:HGNC Symbol;Acc:HGNC:17812]	349,78	156,36	1,16	193,42	0,04
AMPD3	adenosine monophosphate deaminase 3 [Source:HGNC Symbol;Acc:HGNC:470]	74,18	21,37	1,80	52,81	0,02
ANGPT1	angiopoietin 1 [Source:HGNC Symbol;Acc:HGNC:484]	2,71	18,77	-2,79	16,06	0,03
ANGPTL4	angiopoietin like 4 [Source:HGNC Symbol;Acc:HGNC:16039]	49,16	100,77	-1,04	51,61	0,05
ANKRD1	ankyrin repeat domain 1 [Source:NCBI gene;Acc:100538633]	2134,62	420,19	2,34	1714,42	0,01
ANKRD10	ankyrin repeat domain 10 [Source:HGNC Symbol;Acc:HGNC:20265]	218,57	103,48	1,08	115,09	0,04
ANKRD2	ankyrin repeat domain 2 [Source:HGNC Symbol;Acc:HGNC:495]	178,47	68,44	1,38	110,04	0,03
ANLN	anillin actin binding protein [Source:HGNC Symbol;Acc:HGNC:14082]	87,74	24,81	1,82	62,94	0,02
ANO1	anoctamin 1 [Source:HGNC Symbol;Acc:HGNC:21625]	42,71	11,63	1,88	31,08	0,02
ANO2	anoctamin 2 [Source:HGNC Symbol;Acc:HGNC:1183]	33,37	7,36	2,18	26,01	0,02
ANOS1	anosmin 1 [Source:HGNC Symbol;Acc:HGNC:6211]	8,68	30,78	-1,83	22,10	0,03

ANXA7	annexin A7 [Source:HGNC Symbol;Acc:HGNC:545]	233,06	109,35	1,09	123,71	0,04
AP3M1	adaptor related protein complex 3 subunit mu 1 [Source:HGNC Symbol;Acc:HGNC:569]	250,81	110,57	1,18	140,23	0,03
APOA1	apolipoprotein A1 [Source:NCBI gene;Acc:100542499]	80,20	7198,14	-6,49	7117,94	0,00
APOBEC2	apolipoprotein B mRNA editing enzyme catalytic subunit 2 [Source:HGNC Symbol;Acc:HGNC:605]	129,64	54,98	1,24	74,65	0,03
AQP3	aquaporin 3 (Gill blood group) [Source:HGNC Symbol;Acc:HGNC:636]	1,36	82,50	-5,93	81,14	0,00
ARFGAP3	ADP ribosylation factor GTPase activating protein 3 [Source:NCBI gene;Acc:100550221]	86,44	200,05	-1,21	113,61	0,03
ARHGAP24	Rho GTPase activating protein 24 [Source:HGNC Symbol;Acc:HGNC:25361]	73,47	33,39	1,14	40,09	0,04
ARHGAP40	Rho GTPase activating protein 40 [Source:HGNC Symbol;Acc:HGNC:16226]	70,43	17,39	2,02	53,05	0,01
ARHGEF17	Rho guanine nucleotide exchange factor 17 [Source:HGNC Symbol;Acc:HGNC:21726]	185,04	87,76	1,08	97,28	0,04
ARHGEF3	Rho guanine nucleotide exchange factor 3 [Source:NCBI gene;Acc:100546667]	37,98	8,58	2,15	29,40	0,01
ARL6IP5	ADP ribosylation factor like GTPase 6 interacting protein 5 [Source:HGNC Symbol;Acc:HGNC:16937]	844,45	412,66	1,03	431,79	0,05
ARL8A	ADP ribosylation factor like GTPase 8A [Source:HGNC Symbol;Acc:HGNC:25192]	96,05	43,24	1,15	52,80	0,04
ARPC1B	actin related protein 2/3 complex subunit 1B [Source:NCBI gene;Acc:100539832]	138,10	316,43	-1,20	178,33	0,03
ARPP21	cAMP regulated phosphoprotein 21 [Source:HGNC Symbol;Acc:HGNC:16968]	144,07	19,88	2,86	124,19	0,00
ARRDC1	arrestin domain containing 1 [Source:HGNC Symbol;Acc:HGNC:28633]	47,43	16,78	1,50	30,65	0,03
ARVCF	ARVCF delta catenin family member [Source:NCBI gene;Acc:100547080]	60,23	21,04	1,52	39,19	0,03
ASAP3	ArfGAP with SH3 domain, ankyrin repeat and PH domain 3 [Source:HGNC Symbol;Acc:HGNC:14987]	9,17	36,88	-2,01	27,70	0,02
ASB5	ankyrin repeat and SOCS box containing 5 [Source:HGNC Symbol;Acc:HGNC:17180]	553,60	116,61	2,25	436,99	0,01
ASPH	aspartate beta-hydroxylase [Source:HGNC Symbol;Acc:HGNC:757]	98,98	202,26	-1,03	103,28	0,05
ASPM	abnormal spindle microtubule assembly [Source:HGNC Symbol;Acc:HGNC:19048]	42,05	10,52	2,00	31,53	0,02
ATOH8	atonal bHLH transcription factor 8 [Source:HGNC Symbol;Acc:HGNC:24126]	73,31	14,23	2,37	59,08	0,01
ATP1A1	ATPase Na+/K+ transporting subunit alpha 1 [Source:HGNC Symbol;Acc:HGNC:799]	993,40	419,42	1,24	573,98	0,03
ATP2A2	ATPase sarcoplasmic/endoplasmic reticulum Ca2+ transporting 2 [Source:HGNC Symbol;Acc:HGNC:812]	664,02	224,41	1,57	439,61	0,02
ATP2A3	ATPase sarcoplasmic/endoplasmic reticulum Ca2+ transporting 3 [Source:HGNC Symbol;Acc:HGNC:813]	62,78	16,33	1,94	46,45	0,02
ATP2B1	ATPase plasma membrane Ca2+ transporting 1 [Source:HGNC Symbol;Acc:HGNC:814]	98,33	47,95	1,04	50,38	0,05
BAG2	BCL2 associated athanogene 2 [Source:HGNC Symbol;Acc:HGNC:938]	178,96	51,22	1,80	127,74	0,02
BAG3	BCL2 associated athanogene 3 [Source:HGNC Symbol;Acc:HGNC:939]	325,91	152,71	1,09	173,20	0,04
BDH1	3-hydroxybutyrate dehydrogenase 1 [Source:NCBI gene;Acc:100544505]	208,37	54,93	1,92	153,45	0,01
BIN1	bridging integrator 1 [Source:HGNC Symbol;Acc:HGNC:1052]	81,45	30,18	1,43	51,27	0,03
BMP2K	BMP2 inducible kinase [Source:HGNC Symbol;Acc:HGNC:18041]	64,25	26,47	1,28	37,78	0,03
BMPER	BMP binding endothelial regulator [Source:HGNC Symbol;Acc:HGNC:24154]	10,58	62,68	-2,57	52,10	0,01
BNIP3L	BCL2 interacting protein 3 like [Source:HGNC Symbol;Acc:HGNC:1085]	206,80	441,12	-1,09	234,32	0,04
BOC	BOC cell adhesion associated, oncogene regulated [Source:HGNC Symbol;Acc:HGNC:17173]	58,06	122,75	-1,08	64,69	0,04
BPHL	biphenyl hydrolase like [Source:HGNC Symbol;Acc:HGNC:1094]	95,29	33,50	1,51	61,79	0,02
BSG	basigin (Ok blood group) [Source:NCBI gene;Acc:100538809]	448,38	1441,41	-1,68	993,03	0,02
BST1	bone marrow stromal cell antigen 1 [Source:HGNC Symbol;Acc:HGNC:1118]	15,41	40,59	-1,40	25,17	0,04
BTBD11	BTB domain containing 11 [Source:HGNC Symbol;Acc:HGNC:23844]	59,53	24,36	1,29	35,16	0,03
BTBD6	BTB domain containing 6 [Source:HGNC Symbol;Acc:HGNC:19897]	28,87	93,46	-1,69	64,59	0,02
BVES	blood vessel epicardial substance [Source:NCBI gene;Acc:100548587]	168,11	39,59	2,09	128,52	0,01
C12orf75	chromosome 12 open reading frame 75 [Source:HGNC Symbol;Acc:HGNC:35164]	32,88	97,61	-1,57	64,73	0,02
C1R	complement C1r [Source:NCBI gene;Acc:100549554]	2,71	22,92	-3,08	20,21	0,02
C1S	complement C1s [Source:NCBI gene;Acc:100549400]	5,43	79,18	-3,87	73,75	0,00
CA12	carbonic anhydrase 12 [Source:HGNC Symbol;Acc:HGNC:1371]	8,09	52,16	-2,69	44,07	0,01

CA3	carbonic anhydrase 3-like [Source:NCBI gene;Acc:100538829]	6,29	22,81	-1,86	16,52	0,04
CA8	carbonic anhydrase 8 [Source:HGNC Symbol;Acc:HGNC:1382]	43,52	6,15	2,82	37,37	0,01
CACNA1S	calcium voltage-gated channel subunit alpha1 S [Source:HGNC Symbol;Acc:HGNC:1397]	237,51	45,90	2,37	191,61	0,01
CACNG1	calcium voltage-gated channel auxiliary subunit gamma 1 [Source:NCBI gene;Acc:100544356]	192,31	23,75	3,02	168,56	0,00
CACNG4	calcium voltage-gated channel auxiliary subunit gamma 4 [Source:HGNC Symbol;Acc:HGNC:1408]	25,40	7,03	1,85	18,36	0,04
CADM1	cell adhesion molecule 1 [Source:HGNC Symbol;Acc:HGNC:5951]	86,39	23,42	1,88	62,97	0,01
CALD1	caldesmon 1 [Source:HGNC Symbol;Acc:HGNC:1441]	981,14	476,72	1,04	504,41	0,05
CAMK1D	calcium/calmodulin dependent protein kinase ID [Source:HGNC Symbol;Acc:HGNC:19341]	0,76	19,71	-4,70	18,95	0,02
CAMK2A	calcium/calmodulin dependent protein kinase II alpha [Source:HGNC Symbol;Acc:HGNC:1460]	286,67	53,71	2,42	232,97	0,01
CAP2	cyclase associated actin cytoskeleton regulatory protein 2 [Source:NCBI gene;Acc:100548386]	214,29	53,98	1,99	160,30	0,01
CAPG	capping actin protein, gelsolin like [Source:HGNC Symbol;Acc:HGNC:1474]	124,48	252,48	-1,02	128,00	0,05
CAPN11	calpain 11 [Source:HGNC Symbol;Acc:HGNC:1478]	1021,46	483,42	1,08	538,03	0,04
CAPN15	calpain 15 [Source:HGNC Symbol;Acc:HGNC:11182]	115,53	40,59	1,51	74,94	0,02
CAPN6	calpain-5 [Source:NCBI gene;Acc:100540236]	1,47	65,78	-5,49	64,31	0,00
CASKIN2	CASK interacting protein 2 [Source:HGNC Symbol;Acc:HGNC:18200]	60,23	11,57	2,38	48,66	0,01
CASQ2	calsequestrin 2 [Source:NCBI gene;Acc:100547249]	619,69	116,05	2,42	503,64	0,01
CASS4	Cas scaffold protein family member 4 [Source:HGNC Symbol;Acc:HGNC:15878]	233,22	51,33	2,18	181,90	0,01
CASTOR2	cytosolic arginine sensor for mTORC1 subunit 2 [Source:HGNC Symbol;Acc:HGNC:37073]	70,43	25,30	1,48	45,13	0,03
CAT	catalase [Source:HGNC Symbol;Acc:HGNC:1516]	18,78	43,74	-1,22	24,97	0,04
CAV3	caveolin 3 [Source:HGNC Symbol;Acc:HGNC:1529]	382,56	108,58	1,82	273,98	0,01
CAVIN4	caveolae associated protein 4 [Source:NCBI gene;Acc:100549564]	226,82	69,04	1,72	157,78	0,02
CBR1	carbonyl reductase 1 [Source:HGNC Symbol;Acc:HGNC:1548]	29,30	64,84	-1,15	35,53	0,04
CCDC88C	coiled-coil domain containing 88C [Source:HGNC Symbol;Acc:HGNC:19967]	381,91	66,00	2,53	315,91	0,00
CCN3	cellular communication network factor 3 [Source:NCBI gene;Acc:100542737]	0,87	19,21	-4,47	18,34	0,02
CCN4	cellular communication network factor 4 [Source:HGNC Symbol;Acc:HGNC:12769]	2,60	75,41	-4,86	72,81	0,00
CCNH	cyclin H [Source:HGNC Symbol;Acc:HGNC:1594]	41,73	18,60	1,17	23,12	0,05
CD151	CD151 molecule (Raph blood group) [Source:NCBI gene;Acc:100544864]	288,68	127,51	1,18	161,17	0,03
CD36	CD36 molecule [Source:HGNC Symbol;Acc:HGNC:1663]	1,19	33,89	-4,83	32,69	0,01
CDH11	cadherin 11 [Source:HGNC Symbol;Acc:HGNC:1750]	3,96	340,41	-6,43	336,44	0,00
CDH13	cadherin 13 [Source:HGNC Symbol;Acc:HGNC:1753]	169,52	74,86	1,18	94,66	0,04
CDH2	cadherin 2 [Source:NCBI gene;Acc:100542837]	777,11	174,24	2,16	602,86	0,01
CDH23	cadherin related 23 [Source:HGNC Symbol;Acc:HGNC:13733]	41,73	9,36	2,16	32,37	0,01
CDK1	cyclin dependent kinase 1 [Source:NCBI gene;Acc:100549676]	65,44	28,18	1,22	37,26	0,04
CDKN1B	cyclin dependent kinase inhibitor 1B [Source:HGNC Symbol;Acc:HGNC:1785]	303,33	140,19	1,11	163,14	0,04
CDR2	cerebellar degeneration related protein 2 [Source:HGNC Symbol;Acc:HGNC:1799]	28,22	74,03	-1,39	45,81	0,03
CELF2	CUGBP Elav-like family member 2 [Source:NCBI gene;Acc:100538923]	21,16	4,43	2,26	16,73	0,03
CELSR1	cadherin EGF LAG seven-pass G-type receptor 1 [Source:HGNC Symbol;Acc:HGNC:1850]	68,53	20,98	1,71	47,55	0,02
CENPF	centromere protein F [Source:NCBI gene;Acc:100551197]	33,81	6,53	2,37	27,27	0,01
CEP350	centrosomal protein 350 [Source:HGNC Symbol;Acc:HGNC:24238]	25,34	8,03	1,66	17,31	0,04
CEP43	FGFR1 oncogene partner [Source:HGNC Symbol;Acc:HGNC:17012]	28,71	9,69	1,57	19,02	0,04
CERCAM	cerebral endothelial cell adhesion molecule [Source:HGNC Symbol;Acc:HGNC:23723]	148,84	442,06	-1,57	293,22	0,02
CHL1	cell adhesion molecule L1 like [Source:HGNC Symbol;Acc:HGNC:1939]	11,12	186,37	-4,07	175,25	0,00
CHN1	chimerin 1 [Source:HGNC Symbol;Acc:HGNC:1943]	79,88	32,22	1,31	47,65	0,03

CHRDL2	chordin like 2 [Source:NCBI gene;Acc:100551248]	2,28	21,70	-3,25	19,43	0,02
CHRNA1	cholinergic receptor nicotinic alpha 1 subunit [Source:HGNC Symbol;Acc:HGNC:1955]	1851,20	212,28	3,12	1638,91	0,00
CHRND	cholinergic receptor nicotinic delta subunit [Source:HGNC Symbol;Acc:HGNC:1965]	132,57	32,00	2,05	100,56	0,01
CHRNG	cholinergic receptor nicotinic gamma subunit [Source:HGNC Symbol;Acc:HGNC:1967]	264,70	41,36	2,68	223,34	0,00
CHST15	carbohydrate sulfotransferase 15 [Source:HGNC Symbol;Acc:HGNC:18137]	7,16	57,53	-3,01	50,37	0,00
CIP2A	cell proliferation regulating inhibitor of protein phosphatase 2A [Source:HGNC Symbol;Acc:HGNC:29302]	46,34	18,44	1,33	27,90	0,04
CKAP2	cytoskeleton associated protein 2 [Source:HGNC Symbol;Acc:HGNC:1990]	95,18	31,56	1,59	63,62	0,02
CKB	creatine kinase B [Source:NCBI gene;Acc:100550185]	1702,57	560,27	1,60	1142,30	0,02
CLEC3B	C-type lectin domain family 3 member B [Source:NCBI gene;Acc:100540795]	13,89	149,44	-3,43	135,55	0,00
CLMP	CXADR like membrane protein [Source:HGNC Symbol;Acc:HGNC:24039]	18,99	95,07	-2,32	76,08	0,01
CMTM3	CKLF like MARVEL transmembrane domain containing 3 [Source:HGNC Symbol;Acc:HGNC:19174]	24,80	78,79	-1,67	53,99	0,02
CMTM7	CKLF like MARVEL transmembrane domain containing 7 [Source:HGNC Symbol;Acc:HGNC:19178]	30,33	4,98	2,61	25,35	0,01
CNDP2	carnosine dipeptidase 2 [Source:HGNC Symbol;Acc:HGNC:24437]	36,30	136,26	-1,91	99,96	0,01
CNP	2',3'-cyclic nucleotide 3' phosphodiesterase [Source:HGNC Symbol;Acc:HGNC:2158]	255,80	79,79	1,68	176,01	0,02
CNR1	cannabinoid receptor 1 [Source:HGNC Symbol;Acc:HGNC:2159]	161,43	37,71	2,10	123,73	0,01
COBL	cordon-bleu WH2 repeat protein [Source:HGNC Symbol;Acc:HGNC:22199]	132,08	36,99	1,84	95,09	0,01
COL12A1	collagen type XII alpha 1 chain [Source:HGNC Symbol;Acc:HGNC:2188]	1186,96	2791,24	-1,23	1604,28	0,03
COL13A1	collagen type XIII alpha 1 chain [Source:HGNC Symbol;Acc:HGNC:2190]	51,71	12,79	2,02	38,92	0,01
COL14A1	collagen type XIV alpha 1 chain [Source:NCBI gene;Acc:100543201]	0,49	73,25	-7,23	72,76	0,00
COL16A1	collagen type XVI alpha 1 chain [Source:HGNC Symbol;Acc:HGNC:2193]	96,70	1685,53	-4,12	1588,83	0,00
COL18A1	collagen type XVIII alpha 1 chain [Source:HGNC Symbol;Acc:HGNC:2195]	667,06	329,22	1,02	337,84	0,05
COL1A2	collagen type I alpha 2 chain [Source:HGNC Symbol;Acc:HGNC:2198]	15156,00	43852,37	-1,53	28696,37	0,02
COL20A1	collagen type XX alpha 1 chain [Source:HGNC Symbol;Acc:HGNC:14670]	29,84	4,15	2,85	25,69	0,01
COL4A1	collagen type IV alpha 1 chain [Source:NCBI gene;Acc:100550138]	2778,02	931,96	1,58	1846,05	0,02
COL5A1	collagen type V alpha 1 chain [Source:HGNC Symbol;Acc:HGNC:2209]	587,13	2722,70	-2,21	2135,56	0,01
COL6A1	collagen type VI alpha 1 chain [Source:HGNC Symbol;Acc:HGNC:2211]	3825,19	12939,09	-1,76	9113,89	0,02
COL6A2	collagen type VI alpha 2 chain [Source:NCBI gene;Acc:100546041]	4850,56	15002,23	-1,63	10151,67	0,02
COL6A3	collagen type VI alpha 3 chain [Source:NCBI gene;Acc:100549061]	2006,88	9851,79	-2,30	7844,91	0,01
COL8A1	collagen type VIII alpha 1 chain [Source:HGNC Symbol;Acc:HGNC:2215]	12,53	54,48	-2,12	41,95	0,01
COMT	catechol-O-methyltransferase [Source:NCBI gene;Acc:100545849]	115,42	29,84	1,95	85,57	0,01
CORO6	coronin 6 [Source:HGNC Symbol;Acc:HGNC:21356]	63,76	10,63	2,58	53,13	0,01
CPAMD8	C3 and PZP like alpha-2-macroglobulin domain containing 8 [Source:HGNC Symbol;Acc:HGNC:23228]	6,29	41,47	-2,72	35,18	0,01
CPEB4	cytoplasmic polyadenylation element binding protein 4 [Source:HGNC Symbol;Acc:HGNC:21747]	72,71	23,25	1,64	49,46	0,02
CREB3L1	cAMP responsive element binding protein 3 like 1 [Source:HGNC Symbol;Acc:HGNC:18856]	96,43	239,80	-1,31	143,38	0,03
CREM	cAMP responsive element modulator [Source:HGNC Symbol;Acc:HGNC:2352]	11,40	36,43	-1,68	25,04	0,03
CRLF1	cytokine receptor like factor 1 [Source:HGNC Symbol;Acc:HGNC:2364]	132,95	54,54	1,29	78,41	0,03
CRTAC1	cartilage acidic protein 1 [Source:HGNC Symbol;Acc:HGNC:14882]	72,55	12,90	2,49	59,65	0,01
CRYAB	crystallin alpha B [Source:NCBI gene;Acc:100548878]	206,42	43,96	2,23	162,46	0,01
CSGALNACT1	chondroitin sulfate N-acetylgalactosaminyltransferase 1 [Source:HGNC Symbol;Acc:HGNC:24290]	1,63	18,55	-3,51	16,92	0,03
CSRP3	cysteine and glycine rich protein 3 [Source:HGNC Symbol;Acc:HGNC:2472]	172,77	57,42	1,59	115,36	0,02
CSTA	cystatin A [Source:HGNC Symbol;Acc:HGNC:2481]	36,09	14,29	1,34	21,80	0,04
CTBS	chitobiase [Source:HGNC Symbol;Acc:HGNC:2496]	50,84	107,75	-1,08	56,90	0,04
CTHRC1	collagen triple helix repeat containing 1 [Source:HGNC Symbol;Acc:HGNC:18831]	96,10	313,05	-1,70	216,95	0,02

CTNND1	catenin delta 1 [Source:HGNC Symbol;Acc:HGNC:2515]	363,62	170,98	1,09	192,64	0,04
CTSB	cathepsin B [Source:NCBI gene;Acc:100544902]	352,60	715,36	-1,02	362,76	0,05
CTSK	cathepsin K [Source:HGNC Symbol;Acc:HGNC:2536]	107,39	297,77	-1,47	190,38	0,02
CUEDC2	CUE domain containing 2 [Source:NCBI gene;Acc:100550651]	98,27	43,80	1,17	54,47	0,04
CXCL14	C-X-C motif chemokine ligand 14 [Source:HGNC Symbol;Acc:HGNC:10640]	1,36	113,62	-6,39	112,26	0,00
CXXC5	CXXC finger protein 5 [Source:HGNC Symbol;Acc:HGNC:26943]	142,17	68,21	1,06	73,96	0,04
CYBRD1	cytochrome b reductase 1 [Source:HGNC Symbol;Acc:HGNC:20797]	5,53	20,98	-1,92	15,45	0,04
CYGB	cytoglobin [Source:NCBI gene;Acc:100549436]	21,81	2,88	2,92	18,93	0,02
CYP17A1	cytochrome P450 family 17 subfamily A member 1 [Source:HGNC Symbol;Acc:HGNC:2593]	40,70	6,53	2,64	34,16	0,01
CYP21A2	cytochrome P450 family 21 subfamily A member 2 [Source:HGNC Symbol;Acc:HGNC:2600]	32,40	11,35	1,51	21,04	0,04
CYRIA	protein FAM49A [Source:NCBI gene;Acc:100540217]	479,58	104,09	2,20	375,49	0,01
DAB2	DAB adaptor protein 2 [Source:HGNC Symbol;Acc:HGNC:2662]	2,60	22,04	-3,08	19,43	0,02
DACT1	dishevelled binding antagonist of beta catenin 1 [Source:HGNC Symbol;Acc:HGNC:17748]	294,22	55,81	2,40	238,40	0,01
DAG1	dystroglycan 1 [Source:NCBI gene;Acc:100547027]	1039,74	357,52	1,54	682,23	0,02
DAGLB	diacylglycerol lipase beta [Source:NCBI gene;Acc:100548002]	66,09	25,75	1,36	40,35	0,03
DAPK2	death associated protein kinase 2 [Source:HGNC Symbol;Acc:HGNC:2675]	180,91	31,62	2,52	149,30	0,00
DBN1	drebrin 1 [Source:HGNC Symbol;Acc:HGNC:2695]	1387,57	313,05	2,15	1074,52	0,01
DBX1	developing brain homeobox 1 [Source:HGNC Symbol;Acc:HGNC:33185]	24,80	3,71	2,74	21,09	0,02
DCAF6	DDB1 and CUL4 associated factor 6 [Source:HGNC Symbol;Acc:HGNC:30002]	286,24	138,81	1,04	147,43	0,05
DCK	deoxycytidine kinase [Source:HGNC Symbol;Acc:HGNC:2704]	62,29	28,63	1,12	33,67	0,04
DCLK1	doublecortin like kinase 1 [Source:HGNC Symbol;Acc:HGNC:2700]	91,54	25,19	1,86	66,35	0,02
DCLK2	doublecortin like kinase 2 [Source:HGNC Symbol;Acc:HGNC:19002]	29,63	9,91	1,58	19,72	0,04
DCN	decorin [Source:NCBI gene;Acc:100543191]	220,53	2743,24	-3,64	2522,71	0,00
DDHD2	DDHD domain containing 2 [Source:HGNC Symbol;Acc:HGNC:29106]	31,96	11,63	1,46	20,33	0,04
DEPTOR	DEP domain containing MTOR interacting protein [Source:HGNC Symbol;Acc:HGNC:22953]	30,77	8,86	1,80	21,91	0,03
DES	desmin [Source:HGNC Symbol;Acc:HGNC:2770]	2631,94	982,46	1,42	1649,48	0,03
DIP2C	disco interacting protein 2 homolog C [Source:HGNC Symbol;Acc:HGNC:29150]	39,94	16,00	1,32	23,94	0,04
DIPK1C	divergent protein kinase domain 1C [Source:HGNC Symbol;Acc:HGNC:31729]	0,33	69,43	-7,74	69,11	0,00
DISP1	dispatched RND transporter family member 1 [Source:NCBI gene;Acc:100548328]	101,53	27,35	1,89	74,17	0,01
DMD	dystrophin [Source:HGNC Symbol;Acc:HGNC:2928]	122,47	20,76	2,56	101,71	0,00
DMGDH	dimethylglycine dehydrogenase [Source:HGNC Symbol;Acc:HGNC:24475]	109,23	8,80	3,63	100,43	0,00
DMRT2	doublesex and mab-3 related transcription factor 2 [Source:HGNC Symbol;Acc:HGNC:2935]	32,94	3,21	3,36	29,73	0,01
DNAJB5	DnaJ heat shock protein family (Hsp40) member B5 [Source:HGNC Symbol;Acc:HGNC:14887]	187,26	33,77	2,47	153,49	0,01
DNAJB6	DnaJ heat shock protein family (Hsp40) member B6 [Source:NCBI gene;Acc:100546860]	373,33	177,12	1,08	196,21	0,04
DNAJC7	DnaJ heat shock protein family (Hsp40) member C7 [Source:NCBI gene;Acc:100544525]	378,65	162,17	1,22	216,48	0,03
DNER	delta/notch like EGF repeat containing [Source:HGNC Symbol;Acc:HGNC:24456]	35,60	12,90	1,46	22,70	0,04
DNM1	dynamin 1 [Source:HGNC Symbol;Acc:HGNC:2972]	11,83	58,80	-2,31	46,97	0,01
DNMBP	dynamin binding protein [Source:HGNC Symbol;Acc:HGNC:30373]	25,34	57,75	-1,19	32,41	0,04
DOK7	docking protein 7 [Source:HGNC Symbol;Acc:HGNC:26594]	25,78	5,15	2,32	20,63	0,02
DPT	dermatopontin [Source:NCBI gene;Acc:100540516]	0,05	90,36	-10,70	90,31	0,00
DPYSL5	dihydropyrimidinase like 5 [Source:HGNC Symbol;Acc:HGNC:20637]	84,38	10,91	2,95	73,47	0,00
DTNA	dystrobrevin alpha [Source:HGNC Symbol;Acc:HGNC:3057]	19,48	4,43	2,14	15,05	0,04
DTX2	deltex E3 ubiquitin ligase 2 [Source:HGNC Symbol;Acc:HGNC:15973]	201,91	78,01	1,37	123,90	0,03

DUSP1	dual specificity phosphatase 1 [Source:HGNC Symbol;Acc:HGNC:3064]	50,84	17,99	1,50	32,85	0,03
DUSP6	dual specificity phosphatase 6 [Source:HGNC Symbol;Acc:HGNC:3072]	41,02	13,68	1,58	27,35	0,03
DYNLT3	dynein light chain Tctex-type 3 [Source:HGNC Symbol;Acc:HGNC:11694]	7,00	26,19	-1,90	19,19	0,03
EAF2	ELL associated factor 2 [Source:HGNC Symbol;Acc:HGNC:23115]	13,78	49,78	-1,85	35,99	0,02
EBF2	EBF transcription factor 2 [Source:HGNC Symbol;Acc:HGNC:19090]	0,22	13,62	-5,97	13,40	0,04
ECE1	endothelin converting enzyme 1 [Source:HGNC Symbol;Acc:HGNC:3146]	29,63	104,15	-1,81	74,52	0,02
ECHDC2	enoyl-CoA hydratase domain containing 2 [Source:HGNC Symbol;Acc:HGNC:23408]	40,59	106,03	-1,39	65,44	0,03
ECPAS	Ecm29 proteasome adaptor and scaffold [Source:HGNC Symbol;Acc:HGNC:29020]	110,70	50,00	1,15	60,70	0,04
ECRG4	ECRG4 augurin precursor [Source:NCBI gene;Acc:100544482]	6,51	66,94	-3,36	60,43	0,00
EDNRA	endothelin receptor type A [Source:NCBI gene;Acc:100545831]	8,90	123,75	-3,80	114,85	0,00
EEF1A2	eukaryotic translation elongation factor 1 alpha 2 [Source:HGNC Symbol;Acc:HGNC:3192]	437,53	178,45	1,29	259,07	0,03
EEF2K	eukaryotic elongation factor 2 kinase [Source:HGNC Symbol;Acc:HGNC:24615]	12,16	40,75	-1,75	28,60	0,02
EFEMP1	EGF containing fibulin extracellular matrix protein 1 [Source:HGNC Symbol;Acc:HGNC:3218]	1,03	78,35	-6,25	77,32	0,00
EGFL7	EGF like domain multiple 7 [Source:NCBI gene;Acc:100546005]	0,81	26,41	-5,02	25,60	0,01
ELMO1	engulfment and cell motility 1 [Source:NCBI gene;Acc:100549625]	10,36	52,43	-2,34	42,07	0,01
EMILIN2	elastin microfibril interfacer 2 [Source:HGNC Symbol;Acc:HGNC:19881]	102,18	367,54	-1,85	265,36	0,01
ENG	endoglin [Source:HGNC Symbol;Acc:HGNC:3349]	7,81	32,06	-2,04	24,24	0,02
ENO2	enolase 2 [Source:NCBI gene;Acc:100540825]	42,81	130,84	-1,61	88,02	0,02
ENPP2	ectonucleotide pyrophosphatase/phosphodiesterase 2 [Source:HGNC Symbol;Acc:HGNC:3357]	1,14	74,19	-6,02	73,05	0,00
EPHA3	EPH receptor A3 [Source:HGNC Symbol;Acc:HGNC:3387]	2,39	17,50	-2,87	15,11	0,04
EPHA5	EPH receptor A5 [Source:NCBI gene;Acc:100547106]	22,74	2,55	3,16	20,19	0,02
EPHB1	EPH receptor B1 [Source:HGNC Symbol;Acc:HGNC:3392]	171,58	1117,62	-2,70	946,03	0,00
EPN3	epsin 3 [Source:HGNC Symbol;Acc:HGNC:18235]	126,92	33,89	1,91	93,04	0,01
ERAP1	endoplasmic reticulum aminopeptidase 1 [Source:HGNC Symbol;Acc:HGNC:18173]	56,22	10,35	2,44	45,86	0,01
ESPL1	extra spindle pole bodies like 1, separase [Source:HGNC Symbol;Acc:HGNC:16856]	27,35	7,70	1,83	19,65	0,03
ETF1	eukaryotic translation termination factor 1 [Source:HGNC Symbol;Acc:HGNC:3477]	219,82	100,27	1,13	119,55	0,04
ETV4	ETS variant transcription factor 4 [Source:HGNC Symbol;Acc:HGNC:3493]	66,20	29,35	1,17	36,86	0,04
EVA1B	eva-1 homolog B [Source:HGNC Symbol;Acc:HGNC:25558]	5,48	31,67	-2,53	26,19	0,01
EVL	Enah/Vasp-like [Source:HGNC Symbol;Acc:HGNC:20234]	60,45	138,59	-1,20	78,14	0,03
EXOC6	exocyst complex component 6 [Source:HGNC Symbol;Acc:HGNC:23196]	59,80	18,71	1,68	41,08	0,02
EYA1	EYA transcriptional coactivator and phosphatase 1 [Source:HGNC Symbol;Acc:HGNC:3519]	177,66	29,40	2,60	148,26	0,00
EYA2	EYA transcriptional coactivator and phosphatase 2 [Source:HGNC Symbol;Acc:HGNC:3520]	365,95	144,57	1,34	221,39	0,03
EZR	ezrin [Source:HGNC Symbol;Acc:HGNC:12691]	272,13	121,31	1,17	150,82	0,04
F2RL1	F2R like trypsin receptor 1 [Source:HGNC Symbol;Acc:HGNC:3538]	69,08	18,16	1,93	50,92	0,01
FABP4	fatty acid binding protein 4 [Source:HGNC Symbol;Acc:HGNC:3559]	2,71	423,57	-7,29	420,86	0,00
FABP5	fatty acid binding protein 5 [Source:HGNC Symbol;Acc:HGNC:3560]	1,84	77,02	-5,38	75,17	0,00
FAM13B	family with sequence similarity 13 member B [Source:HGNC Symbol;Acc:HGNC:1335]	44,66	14,84	1,59	29,82	0,03
FAM163B	family with sequence similarity 163 member B [Source:NCBI gene;Acc:100541070]	18,18	3,49	2,38	14,69	0,04
FAM20A	FAM20A golgi associated secretory pathway pseudokinase [Source:HGNC Symbol;Acc:HGNC:23015]	11,50	37,26	-1,70	25,76	0,03
FAM234A	family with sequence similarity 234 member A [Source:HGNC Symbol;Acc:HGNC:14163]	117,43	48,06	1,29	69,37	0,03
FAT3	FAT atypical cadherin 3 [Source:HGNC Symbol;Acc:HGNC:23112]	27,89	1,11	4,65	26,78	0,01
FAT4	FAT atypical cadherin 4 [Source:HGNC Symbol;Acc:HGNC:23109]	16,39	42,14	-1,36	25,75	0,04
FBLIM1	filamin binding LIM protein 1 [Source:HGNC Symbol;Acc:HGNC:24686]	32,45	74,53	-1,20	42,08	0,04

FBLN1	fibulin 1 [Source:HGNC Symbol;Acc:HGNC:3600]	148,03	647,09	-2,13	499,06	0,01
FBLN2	fibulin 2 [Source:HGNC Symbol;Acc:HGNC:3601]	297,85	908,71	-1,61	610,86	0,02
FBN1	fibrillin 1 [Source:HGNC Symbol;Acc:HGNC:3603]	37,06	479,55	-3,69	442,49	0,00
FEZ1	fasciculation and elongation protein zeta 1 [Source:NCBI gene;Acc:100542765]	33,05	80,06	-1,28	47,02	0,03
FGF10	fibroblast growth factor 10 [Source:HGNC Symbol;Acc:HGNC:3666]	5,97	21,93	-1,88	15,96	0,04
FGF16	fibroblast growth factor 16 [Source:HGNC Symbol;Acc:HGNC:3672]	0,27	35,99	-7,05	35,72	0,00
FGF7	fibroblast growth factor 7 [Source:NCBI gene;Acc:100550759]	3,42	18,05	-2,40	14,63	0,04
FGFBP1	fibroblast growth factor binding protein 1 [Source:NCBI gene;Acc:100546142]	1,03	15,00	-3,86	13,97	0,04
FGFRL1	fibroblast growth factor receptor like 1 [Source:HGNC Symbol;Acc:HGNC:3693]	12,26	75,91	-2,63	63,65	0,00
FHL1	four and a half LIM domains 1 [Source:HGNC Symbol;Acc:HGNC:3702]	356,95	133,60	1,42	223,34	0,03
FHOD1	formin homology 2 domain containing 1 [Source:HGNC Symbol;Acc:HGNC:17905]	179,29	60,74	1,56	118,55	0,02
FHOD3	formin homology 2 domain containing 3 [Source:HGNC Symbol;Acc:HGNC:26178]	19,97	5,15	1,96	14,82	0,05
FKBP1B	FKBP prolyl isomerase 1B [Source:HGNC Symbol;Acc:HGNC:3712]	133,65	44,07	1,60	89,58	0,02
FKBP9	FKBP prolyl isomerase 9 [Source:HGNC Symbol;Acc:HGNC:3725]	199,42	431,32	-1,11	231,90	0,04
FKTN	fukutin [Source:NCBI gene;Acc:100545765]	53,34	12,51	2,09	40,83	0,01
FLOT2	flotillin 2 [Source:HGNC Symbol;Acc:HGNC:3758]	41,62	89,70	-1,11	48,08	0,04
FLRT3	fibronectin leucine rich transmembrane protein 3 [Source:NCBI gene;Acc:100544379]	13,46	43,08	-1,68	29,62	0,02
FN1	fibronectin 1 [Source:HGNC Symbol;Acc:HGNC:3778]	2689,19	12133,42	-2,17	9444,23	0,01
FNDC1	fibronectin type III domain containing 1 [Source:HGNC Symbol;Acc:HGNC:21184]	31,53	483,76	-3,94	452,23	0,00
FNDC5	fibronectin type III domain containing 5 [Source:HGNC Symbol;Acc:HGNC:20240]	797,46	132,16	2,59	665,29	0,00
FOXM1	forkhead box M1 [Source:NCBI gene;Acc:100540784]	41,24	10,58	1,96	30,66	0,02
FRMD4B	FERM domain containing 4B [Source:HGNC Symbol;Acc:HGNC:24886]	28,60	4,87	2,55	23,72	0,02
FRZB	frizzled related protein [Source:HGNC Symbol;Acc:HGNC:3959]	5,53	34,33	-2,63	28,79	0,01
FSTL1	follistatin like 1 [Source:HGNC Symbol;Acc:HGNC:3972]	135,06	372,58	-1,46	237,51	0,02
FSTL3	follistatin like 3 [Source:HGNC Symbol;Acc:HGNC:3973]	11,99	36,82	-1,62	24,83	0,03
FXR1	FMR1 autosomal homolog 1 [Source:HGNC Symbol;Acc:HGNC:4023]	346,31	153,26	1,18	193,05	0,03
FXYD6	FXYD domain containing ion transport regulator 6 [Source:NCBI gene;Acc:100544836]	807,22	396,27	1,03	410,95	0,05
FZD9	frizzled class receptor 9 [Source:HGNC Symbol;Acc:HGNC:4047]	312,23	111,40	1,49	200,83	0,02
GALNT1	polypeptide N-acetylgalactosaminyltransferase 1 [Source:NCBI gene;Acc:100544386]	200,02	585,63	-1,55	385,62	0,02
GALNT16	polypeptide N-acetylgalactosaminyltransferase 16 [Source:HGNC Symbol;Acc:HGNC:23233]	4,94	38,65	-2,97	33,71	0,01
GALNT18	polypeptide N-acetylgalactosaminyltransferase 18 [Source:HGNC Symbol;Acc:HGNC:30488]	80,36	20,82	1,95	59,55	0,01
GALNT5	polypeptide N-acetylgalactosaminyltransferase 5 [Source:HGNC Symbol;Acc:HGNC:4127]	6,35	32,50	-2,36	26,15	0,02
GAS6	growth arrest specific 6 [Source:HGNC Symbol;Acc:HGNC:4168]	7,98	34,05	-2,09	26,07	0,02
GDPD5	glycerophosphodiester phosphodiesterase domain containing 5 [Source:HGNC Symbol;Acc:HGNC:28804]	8,74	27,57	-1,66	18,84	0,04
GEM	GTP binding protein overexpressed in skeletal muscle [Source:HGNC Symbol;Acc:HGNC:4234]	23,50	444,22	-4,24	420,73	0,00
GFAP	glial fibrillary acidic protein [Source:HGNC Symbol;Acc:HGNC:4235]	50,52	15,61	1,69	34,91	0,02
GFPT2	glutamine-fructose-6-phosphate transaminase 2 [Source:HGNC Symbol;Acc:HGNC:4242]	0,38	105,42	-8,12	105,04	0,00
GJA1	gap junction protein alpha 1 [Source:HGNC Symbol;Acc:HGNC:4274]	195,89	851,74	-2,12	655,84	0,01
GJA5	gap junction protein alpha 5 [Source:NCBI gene;Acc:100543752]	60,99	19,82	1,62	41,17	0,02
GJD4	gap junction protein delta 4 [Source:HGNC Symbol;Acc:HGNC:23296]	34,46	7,64	2,17	26,82	0,02
GK5	glycerol kinase 5 [Source:HGNC Symbol;Acc:HGNC:28635]	18,18	41,08	-1,18	22,91	0,05
GLT8D2	glycosyltransferase 8 domain containing 2 [Source:NCBI gene;Acc:100543348]	4,88	51,94	-3,41	47,05	0,00
GLUL	glutamate-ammonia ligase [Source:HGNC Symbol;Acc:HGNC:4341]	4,83	23,64	-2,29	18,81	0,03

GMPR	guanosine monophosphate reductase [Source:HGNC Symbol;Acc:HGNC:4376]	42,11	9,58	2,14	32,53	0,01
GNAL	G protein subunit alpha L [Source:HGNC Symbol;Acc:HGNC:4388]	40,91	15,89	1,36	25,02	0,04
GNMT	glycine N-methyltransferase [Source:NCBI gene;Acc:100540940]	202,89	64,62	1,65	138,28	0,02
GPBP1	GC-rich promoter binding protein 1 [Source:HGNC Symbol;Acc:HGNC:29520]	38,91	16,78	1,21	22,13	0,05
GPC1	glypican 1 [Source:HGNC Symbol;Acc:HGNC:4449]	511,60	98,45	2,38	413,15	0,01
GPD1L	glycerol-3-phosphate dehydrogenase 1 like [Source:HGNC Symbol;Acc:HGNC:28956]	53,40	21,98	1,28	31,41	0,04
GPR176	G protein-coupled receptor 176 [Source:HGNC Symbol;Acc:HGNC:32370]	57,52	12,46	2,21	45,06	0,01
GPSM1	G protein signaling modulator 1 [Source:HGNC Symbol;Acc:HGNC:17858]	12,10	52,43	-2,12	40,33	0,01
GPX3	glutathione peroxidase 3 [Source:HGNC Symbol;Acc:HGNC:4555]	318,69	849,02	-1,41	530,33	0,03
GPX7	glutathione peroxidase 7 [Source:NCBI gene;Acc:100545645]	15,25	48,34	-1,66	33,09	0,02
GRB10	growth factor receptor bound protein 10 [Source:NCBI gene;Acc:100542734]	63,33	22,65	1,48	40,68	0,03
GRIA3	glutamate ionotropic receptor AMPA type subunit 3 [Source:NCBI gene;Acc:100542232]	21,05	3,43	2,62	17,62	0,03
GSE1	Gse1 coiled-coil protein [Source:HGNC Symbol;Acc:HGNC:28979]	259,65	95,40	1,44	164,25	0,02
GTF2IRD1	GTF2I repeat domain containing 1 [Source:HGNC Symbol;Acc:HGNC:4661]	98,49	20,04	2,30	78,44	0,01
HACD1	3-hydroxyacyl-CoA dehydratase 1 [Source:HGNC Symbol;Acc:HGNC:9639]	48,62	19,21	1,34	29,41	0,03
HAPLN3	hyaluronan and proteoglycan link protein 3 [Source:HGNC Symbol;Acc:HGNC:21446]	2,60	16,39	-2,65	13,78	0,04
HAS2	hyaluronan synthase 2 [Source:HGNC Symbol;Acc:HGNC:4819]	9,50	103,04	-3,44	93,54	0,00
HDAC1	histone deacetylase 1 [Source:NCBI gene;Acc:100546927]	100,17	220,03	-1,14	119,86	0,04
HECW2	HECT, C2 and WW domain containing E3 ubiquitin protein ligase 2 [Source:HGNC Symbol;Acc:HGNC:29853]	265,40	31,67	3,07	233,73	0,00
HERC2	HECT and RLD domain containing E3 ubiquitin protein ligase 2 [Source:HGNC Symbol;Acc:HGNC:4868]	279,19	131,45	1,09	147,74	0,04
HEY1	hes related family bHLH transcription factor with YRPW motif 1 [Source:HGNC Symbol;Acc:HGNC:4880]	55,67	7,31	2,93	48,37	0,00
HEY2	hes related family bHLH transcription factor with YRPW motif 2 [Source:HGNC Symbol;Acc:HGNC:4881]	8,19	35,05	-2,10	26,85	0,02
HIC1	HIC ZBTB transcriptional repressor 1 [Source:HGNC Symbol;Acc:HGNC:4909]	116,88	326,29	-1,48	209,40	0,02
HIF1A	hypoxia inducible factor 1 subunit alpha [Source:HGNC Symbol;Acc:HGNC:4910]	21,76	83,88	-1,95	62,12	0,01
HINT1	histidine triad nucleotide binding protein 1 [Source:HGNC Symbol;Acc:HGNC:4912]	91,33	31,34	1,54	59,99	0,02
HIPK2	homeodomain interacting protein kinase 2 [Source:NCBI gene;Acc:100547660]	161,87	54,43	1,57	107,44	0,02
HMGB2	high mobility group box 2 [Source:NCBI gene;Acc:100549109]	57,09	26,41	1,11	30,67	0,05
HMOX1	heme oxygenase 1 [Source:NCBI gene;Acc:100551243]	47,64	153,76	-1,69	106,12	0,02
HOGA1	4-hydroxy-2-oxoglutarate aldolase 1 [Source:NCBI gene;Acc:100548398]	39,61	11,13	1,83	28,48	0,02
HOPX	HOP homeobox [Source:NCBI gene;Acc:100546449]	503,13	169,26	1,57	333,87	0,02
HOXC6	homeobox C6 [Source:HGNC Symbol;Acc:HGNC:5128]	0,27	25,47	-6,55	25,20	0,01
HOXD8	homeobox D8 [Source:HGNC Symbol;Acc:HGNC:5139]	2,44	43,63	-4,16	41,19	0,00
HPCAL1	hippocalcin like 1 [Source:NCBI gene;Acc:100550125]	56,54	160,24	-1,50	103,69	0,02
HPGDS	hematopoietic prostaglandin D synthase [Source:HGNC Symbol;Acc:HGNC:17890]	1,30	26,91	-4,37	25,61	0,01
HSD17B7	hydroxysteroid 17-beta dehydrogenase 7 [Source:NCBI gene;Acc:100542640]	37,55	83,77	-1,16	46,22	0,04
HSF2	heat shock transcription factor 2 [Source:HGNC Symbol;Acc:HGNC:5225]	281,68	101,49	1,47	180,19	0,02
HSPB1	heat shock protein family B (small) member 1 [Source:NCBI gene;Acc:100544357]	988,08	429,88	1,20	558,20	0,03
HSPB8	heat shock protein family B (small) member 8 [Source:HGNC Symbol;Acc:HGNC:30171]	810,80	196,89	2,04	613,91	0,01
IDH3A	isocitrate dehydrogenase (NAD(+)) 3 catalytic subunit alpha [Source:NCBI gene;Acc:100549122]	271,53	128,23	1,08	143,30	0,04
IDH3B	isocitrate dehydrogenase (NAD(+)) 3 non-catalytic subunit beta [Source:HGNC Symbol;Acc:HGNC:5385]	143,58	63,12	1,19	80,46	0,03
IFFO1	intermediate filament family orphan 1 [Source:HGNC Symbol;Acc:HGNC:24970]	87,26	26,74	1,71	60,51	0,02
IGFBP4	insulin like growth factor binding protein 4 [Source:HGNC Symbol;Acc:HGNC:5473]	78,25	341,40	-2,13	263,15	0,01
IGSF3	immunoglobulin superfamily member 3 [Source:HGNC Symbol;Acc:HGNC:5950]	29,14	9,08	1,68	20,06	0,03

IL1RAP	interleukin 1 receptor accessory protein [Source:HGNC Symbol;Acc:HGNC:5995]	11,34	32,06	-1,50	20,72	0,04
IL1RAPL2	interleukin 1 receptor accessory protein like 2 [Source:HGNC Symbol;Acc:HGNC:5997]	47,64	10,46	2,19	37,18	0,01
IL1RL1	interleukin 1 receptor like 1 [Source:NCBI gene;Acc:100542982]	2,39	31,84	-3,74	29,45	0,01
INHBA	inhibin subunit beta A [Source:HGNC Symbol;Acc:HGNC:6066]	34,46	78,07	-1,18	43,61	0,04
INKA2	inka box actin regulator 2 [Source:HGNC Symbol;Acc:HGNC:28045]	48,29	14,73	1,71	33,57	0,02
IP6K3	inositol hexakisphosphate kinase 3 [Source:NCBI gene;Acc:100544318]	74,02	14,23	2,38	59,79	0,01
IQGAP2	IQ motif containing GTPase activating protein 2 [Source:HGNC Symbol;Acc:HGNC:6111]	35,49	73,36	-1,05	37,88	0,05
ISLR2	immunoglobulin superfamily containing leucine rich repeat 2 [Source:HGNC Symbol;Acc:HGNC:29286]	2,39	20,71	-3,12	18,32	0,02
ITGA11	integrin subunit alpha 11 [Source:NCBI gene;Acc:100547226]	2,28	48,06	-4,40	45,78	0,00
ITGA6	integrin subunit alpha 6 [Source:NCBI gene;Acc:100538428]	613,99	70,21	3,13	543,78	0,00
ITGB1BP2	integrin subunit beta 1 binding protein 2 [Source:HGNC Symbol;Acc:HGNC:6154]	39,83	9,19	2,12	30,64	0,01
ITGB5	integrin subunit beta 5 [Source:HGNC Symbol;Acc:HGNC:6160]	193,23	438,57	-1,18	245,34	0,03
ITPK1	inositol-tetrakisphosphate 1-kinase [Source:NCBI gene;Acc:100541150]	198,55	46,29	2,10	152,26	0,01
ITPR2	inositol 1,4,5-trisphosphate receptor type 2 [Source:HGNC Symbol;Acc:HGNC:6181]	6,29	48,95	-2,96	42,65	0,00
JAG2	jagged canonical Notch ligand 2 [Source:HGNC Symbol;Acc:HGNC:6189]	88,18	29,51	1,58	58,67	0,02
JAKMIP2	janus kinase and microtubule interacting protein 2 [Source:NCBI gene;Acc:100539315]	41,46	13,90	1,58	27,56	0,03
JAM3	junctional adhesion molecule 3 [Source:HGNC Symbol;Acc:HGNC:15532]	438,61	104,20	2,07	334,41	0,01
JCHAIN	joining chain of multimeric IgA and IgM [Source:NCBI gene;Acc:100545573]	146,29	61,24	1,26	85,06	0,03
JPH1	junctophilin 1 [Source:HGNC Symbol;Acc:HGNC:14201]	34,19	7,92	2,11	26,27	0,02
JPH2	junctophilin 2 [Source:HGNC Symbol;Acc:HGNC:14202]	288,84	22,87	3,66	265,98	0,00
KCNE4	potassium voltage-gated channel subfamily E regulatory subunit 4 [Source:NCBI gene;Acc:100550095]	0,43	31,56	-6,18	31,13	0,01
KCNH6	potassium voltage-gated channel subfamily H member 6 [Source:HGNC Symbol;Acc:HGNC:18862]	73,47	16,56	2,15	56,92	0,01
KCNJ2	potassium inwardly rectifying channel subfamily J member 2 [Source:NCBI gene;Acc:100544982]	23,77	7,31	1,70	16,46	0,04
KCNJ5	potassium inwardly rectifying channel subfamily J member 5 [Source:HGNC Symbol;Acc:HGNC:6266]	43,03	8,19	2,39	34,84	0,01
KCNMA1	potassium calcium-activated channel subfamily M alpha 1 [Source:HGNC Symbol;Acc:HGNC:6284]	31,91	9,91	1,69	22,00	0,03
KCNT1	potassium sodium-activated channel subfamily T member 1 [Source:HGNC Symbol;Acc:HGNC:18865]	55,62	5,92	3,23	49,70	0,00
KCTD3	potassium channel tetramerization domain containing 3 [Source:HGNC Symbol;Acc:HGNC:21305]	199,58	63,73	1,65	135,85	0,02
KDELR3	KDEL endoplasmic reticulum protein retention receptor 3 [Source:HGNC Symbol;Acc:HGNC:6306]	55,78	115,94	-1,06	60,16	0,05
KIAA0232	KIAA0232 [Source:HGNC Symbol;Acc:HGNC:28992]	58,98	21,04	1,49	37,94	0,03
KIAA0408	KIAA0408 ortholog [Source:NCBI gene;Acc:100546395]	23,93	4,32	2,47	19,61	0,02
KIAA1217	KIAA1217 [Source:HGNC Symbol;Acc:HGNC:25428]	82,21	28,85	1,51	53,36	0,02
KIAA1755	KIAA1755 [Source:HGNC Symbol;Acc:HGNC:29372]	7,87	45,90	-2,54	38,03	0,01
KIF13A	kinesin family member 13A [Source:HGNC Symbol;Acc:HGNC:14566]	36,84	15,39	1,26	21,45	0,05
KIF15	kinesin family member 15 [Source:HGNC Symbol;Acc:HGNC:17273]	35,16	13,12	1,42	22,04	0,04
KIF20A	kinesin family member 20A [Source:HGNC Symbol;Acc:HGNC:9787]	81,88	29,29	1,48	52,59	0,02
KIF23	kinesin family member 23 [Source:HGNC Symbol;Acc:HGNC:6392]	54,64	20,87	1,39	33,77	0,03
KIF26A	kinesin family member 26A [Source:HGNC Symbol;Acc:HGNC:20226]	157,15	480,21	-1,61	323,06	0,02
KIF4A	kinesin family member 4A [Source:HGNC Symbol;Acc:HGNC:13339]	49,05	22,04	1,15	27,02	0,04
KIFC3	kinesin family member C3 [Source:HGNC Symbol;Acc:HGNC:6326]	304,31	84,99	1,84	219,32	0,01
KLF5	Kruppel like factor 5 [Source:HGNC Symbol;Acc:HGNC:6349]	149,12	41,58	1,84	107,53	0,01
KLHDC8A	kelch domain containing 8A [Source:HGNC Symbol;Acc:HGNC:25573]	46,83	19,32	1,28	27,51	0,04
KLHL40	kelch like family member 40 [Source:NCBI gene;Acc:100551211]	139,19	63,95	1,12	75,24	0,04
KREMEN1	kringle containing transmembrane protein 1 [Source:HGNC Symbol;Acc:HGNC:17550]	41,46	7,92	2,39	33,54	0,01

KRT19	keratin, type I cytoskeletal 19-like [Source:NCBI gene;Acc:100541022]	256,34	26,74	3,26	229,60	0,00
KSR1	kinase suppressor of ras 1 [Source:HGNC Symbol;Acc:HGNC:6465]	36,79	9,41	1,97	27,38	0,02
LAMA2	laminin subunit alpha 2 [Source:HGNC Symbol;Acc:HGNC:6482]	113,30	27,35	2,05	85,95	0,01
LAMA5	laminin subunit alpha 5 [Source:HGNC Symbol;Acc:HGNC:6485]	877,11	318,87	1,46	558,25	0,02
LAMB2	laminin subunit beta 2 [Source:HGNC Symbol;Acc:HGNC:6487]	1100,41	437,58	1,33	662,83	0,03
LAMC2	laminin subunit gamma 2 [Source:HGNC Symbol;Acc:HGNC:6493]	21,65	6,04	1,84	15,62	0,05
LAMP5	lysosomal associated membrane protein family member 5 [Source:HGNC Symbol;Acc:HGNC:16097]	57,03	8,19	2,80	48,84	0,00
LBH	LBH regulator of WNT signaling pathway [Source:NCBI gene;Acc:100540885]	1227,39	383,82	1,68	843,57	0,02
LDB2	LIM domain binding 2 [Source:HGNC Symbol;Acc:HGNC:6533]	6,62	23,03	-1,80	16,41	0,04
LDHA	lactate dehydrogenase A [Source:HGNC Symbol;Acc:HGNC:6535]	854,32	2360,53	-1,47	1506,21	0,02
LGALS1	galectin 1 [Source:NCBI gene;Acc:100548120]	444,20	1090,65	-1,30	646,45	0,03
LGALS8	galectin 8 [Source:NCBI gene;Acc:100550840]	63,76	28,07	1,18	35,69	0,04
LIMD1	LIM domains containing 1 [Source:HGNC Symbol;Acc:HGNC:6612]	135,39	65,89	1,04	69,50	0,05
LIMD2	LIM domain containing 2 [Source:NCBI gene;Acc:100542561]	0,16	17,39	-6,74	17,22	0,03
LIX1L	limb and CNS expressed 1 like [Source:HGNC Symbol;Acc:HGNC:28715]	12,91	35,16	-1,44	22,24	0,04
LMO7	LIM domain 7 [Source:HGNC Symbol;Acc:HGNC:6646]	1060,25	168,54	2,65	891,71	0,00
LMOD2	leiomodin 2 [Source:HGNC Symbol;Acc:HGNC:6648]	1096,56	188,42	2,54	908,14	0,00
LMOD3	leiomodin 3 [Source:HGNC Symbol;Acc:HGNC:6649]	35,49	5,09	2,80	30,39	0,01
LRIG3	leucine rich repeats and immunoglobulin like domains 3 [Source:HGNC Symbol;Acc:HGNC:30991]	71,57	28,68	1,32	42,89	0,03
LRP3	LDL receptor related protein 3 [Source:HGNC Symbol;Acc:HGNC:6695]	25,34	7,92	1,68	17,42	0,04
LRRC17	leucine rich repeat containing 17 [Source:NCBI gene;Acc:100543703]	6,29	23,25	-1,89	16,96	0,04
LRRN4	leucine rich repeat neuronal 4 [Source:HGNC Symbol;Acc:HGNC:16208]	10,58	33,28	-1,65	22,70	0,03
LSP1	lymphocyte specific protein 1 [Source:HGNC Symbol;Acc:HGNC:6707]	813,84	385,37	1,08	428,48	0,04
LTBP2	latent transforming growth factor beta binding protein 2 [Source:HGNC Symbol;Acc:HGNC:6715]	103,48	32,00	1,69	71,48	0,02
LUM	lumican [Source:NCBI gene;Acc:100543036]	41,89	1954,95	-5,54	1913,06	0,00
LYSMD2	LysM domain containing 2 [Source:NCBI gene;Acc:100549277]	82,97	39,70	1,06	43,27	0,05
MAB21L1	mab-21 like 1 [Source:HGNC Symbol;Acc:HGNC:6757]	1,19	50,94	-5,42	49,75	0,00
MACF1	microtubule actin crosslinking factor 1 [Source:HGNC Symbol;Acc:HGNC:13664]	607,32	235,43	1,37	371,89	0,03
MAFA	MAF bZIP transcription factor A [Source:NCBI gene;Acc:100541507]	102,34	20,32	2,33	82,02	0,01
MAFB	MAF bZIP transcription factor B [Source:NCBI gene;Acc:100546880]	55,08	291,07	-2,40	236,00	0,01
MAGI1	membrane associated guanylate kinase, WW and PDZ domain containing 1 [Source:HGNC Symbol;Acc:HGNC:946]	38,96	16,33	1,25	22,63	0,04
MAMDC2	MAM domain containing 2 [Source:HGNC Symbol;Acc:HGNC:23673]	483,27	84,88	2,51	398,39	0,00
MAN2A2	mannosidase alpha class 2A member 2 [Source:NCBI gene;Acc:100539000]	190,57	87,48	1,12	103,09	0,04
MAOA	monoamine oxidase A [Source:HGNC Symbol;Acc:HGNC:6833]	2,77	20,21	-2,87	17,44	0,03
MAP1A	microtubule associated protein 1A [Source:HGNC Symbol;Acc:HGNC:6835]	688,44	199,22	1,79	489,22	0,02
MAP1B	microtubule associated protein 1B [Source:HGNC Symbol;Acc:HGNC:6836]	19,37	89,70	-2,21	70,33	0,01
MAP1LC3C	microtubule associated protein 1 light chain 3 gamma [Source:HGNC Symbol;Acc:HGNC:13353]	47,10	15,34	1,62	31,76	0,02
MAPRE1	microtubule associated protein RP/EB family member 1 [Source:NCBI gene;Acc:100545907]	721,54	348,88	1,05	372,66	0,04
MAPRE2	microtubule associated protein RP/EB family member 2 [Source:NCBI gene;Acc:100546702]	71,03	175,13	-1,30	104,10	0,03
MCAM	melanoma cell adhesion molecule [Source:HGNC Symbol;Acc:HGNC:6934]	462,11	39,26	3,56	422,85	0,00
MCFD2	multiple coagulation factor deficiency 2 [Source:NCBI gene;Acc:100546237]	67,83	139,81	-1,04	71,98	0,05
MCM2	minichromosome maintenance complex component 2 [Source:HGNC Symbol;Acc:HGNC:6944]	83,57	36,54	1,19	47,02	0,04
MCM3	minichromosome maintenance complex component 3 [Source:HGNC Symbol;Acc:HGNC:6945]	62,67	21,32	1,56	41,36	0,02

MCM5	minichromosome maintenance complex component 5 [Source:HGNC Symbol;Acc:HGNC:6948]	68,97	29,12	1,24	39,84	0,04
MCU	mitochondrial calcium uniporter [Source:HGNC Symbol;Acc:HGNC:23526]	167,02	46,40	1,85	120,62	0,01
MDFI	MyoD family inhibitor [Source:HGNC Symbol;Acc:HGNC:6967]	2,93	16,22	-2,47	13,29	0,05
MECOM	MDS1 and EVI1 complex locus [Source:NCBI gene;Acc:100541414]	0,71	83,22	-6,88	82,51	0,00
MEF2A	myocyte enhancer factor 2A [Source:HGNC Symbol;Acc:HGNC:6993]	95,18	38,32	1,31	56,86	0,03
MEF2C	myocyte enhancer factor 2C [Source:HGNC Symbol;Acc:HGNC:6996]	41,08	8,58	2,26	32,50	0,01
MEIS2	Meis homeobox 2 [Source:HGNC Symbol;Acc:HGNC:7001]	1,36	27,91	-4,36	26,55	0,01
MELK	maternal embryonic leucine zipper kinase [Source:NCBI gene;Acc:100549393]	111,19	34,33	1,70	76,86	0,02
MEOX2	mesenchyme homeobox 2 [Source:NCBI gene;Acc:100545270]	0,92	17,88	-4,28	16,96	0,03
MET	MET proto-oncogene, receptor tyrosine kinase [Source:NCBI gene;Acc:100544894]	25,34	3,99	2,67	21,35	0,02
METRNL	meteorin like, glial cell differentiation regulator [Source:HGNC Symbol;Acc:HGNC:27584]	103,81	218,32	-1,07	114,51	0,04
METTL24	methyltransferase like 24 [Source:HGNC Symbol;Acc:HGNC:21566]	116,50	38,87	1,58	77,63	0,02
MFSD2A	major facilitator superfamily domain containing 2A [Source:HGNC Symbol;Acc:HGNC:25897]	72,88	33,00	1,14	39,88	0,04
MGAT4B	alpha-1,3-mannosyl-glycoprotein 4-beta-N-acetylglucosaminyltransferase B [Source:HGNC Symbol;Acc:HGNC:7048]	114,33	49,44	1,21	64,89	0,03
MGP	matrix Gla protein [Source:NCBI gene;Acc:100542353]	169,79	24,47	2,79	145,32	0,00
MICAL1	microtubule associated monooxygenase, calponin and LIM domain containing 1 [Source:HGNC Symbol;Acc:HGNC:20619]	125,95	51,27	1,30	74,67	0,03
MICALL1	MICAL like 1 [Source:HGNC Symbol;Acc:HGNC:29804]	1556,49	317,04	2,30	1239,45	0,01
MID1	midline 1 [Source:HGNC Symbol;Acc:HGNC:7095]	115,91	30,56	1,92	85,34	0,01
MIF	macrophage migration inhibitory factor [Source:HGNC Symbol;Acc:HGNC:7097]	164,53	399,04	-1,28	234,51	0,03
MMP11	matrix metallopeptidase 11 [Source:HGNC Symbol;Acc:HGNC:7157]	73,36	156,14	-1,09	82,78	0,04
MMP13	matrix metallopeptidase 13 [Source:HGNC Symbol;Acc:HGNC:7159]	1,30	64,50	-5,63	63,20	0,00
MMP16	matrix metallopeptidase 16 [Source:HGNC Symbol;Acc:HGNC:7162]	7,87	25,97	-1,72	18,10	0,04
MMP2	matrix metallopeptidase 2 [Source:HGNC Symbol;Acc:HGNC:7166]	1241,49	4834,34	-1,96	3592,85	0,01
MMP23B	matrix metallopeptidase 23B [Source:HGNC Symbol;Acc:HGNC:7171]	5,05	109,85	-4,44	104,80	0,00
MMP28	matrix metallopeptidase 28 [Source:HGNC Symbol;Acc:HGNC:14366]	40,59	4,32	3,23	36,27	0,01
MMP9	matrix metallopeptidase 9 [Source:NCBI gene;Acc:100540313]	47,32	229,28	-2,28	181,96	0,01
MOSPD2	motile sperm domain containing 2 [Source:HGNC Symbol;Acc:HGNC:28381]	199,26	49,78	2,00	149,48	0,01
MRAS	muscle RAS oncogene homolog [Source:HGNC Symbol;Acc:HGNC:7227]	1053,42	282,82	1,90	770,59	0,01
MTFR1L	mitochondrial fission regulator 1 like [Source:HGNC Symbol;Acc:HGNC:28836]	231,33	92,74	1,32	138,58	0,03
MTMR3	myotubularin related protein 3 [Source:HGNC Symbol;Acc:HGNC:7451]	58,44	26,30	1,15	32,14	0,04
MTSS2	MTSS I-BAR domain containing 2 [Source:HGNC Symbol;Acc:HGNC:25094]	334,86	97,89	1,77	236,97	0,02
MUSK	muscle associated receptor tyrosine kinase [Source:HGNC Symbol;Acc:HGNC:7525]	99,90	11,74	3,09	88,16	0,00
MYADML2	myeloid associated differentiation marker like 2 [Source:HGNC Symbol;Acc:HGNC:34548]	28,81	7,70	1,90	21,12	0,03
MYBPH	myosin binding protein H [Source:HGNC Symbol;Acc:HGNC:7552]	643,62	224,30	1,52	419,32	0,02
MYCL	MYCL proto-oncogene, bHLH transcription factor [Source:HGNC Symbol;Acc:HGNC:7555]	63,16	21,48	1,56	41,68	0,02
MYCN	MYCN proto-oncogene, bHLH transcription factor [Source:HGNC Symbol;Acc:HGNC:7559]	15,63	2,55	2,62	13,08	0,05
MYL3	myosin light chain 3 [Source:NCBI gene;Acc:100538682]	932,63	258,07	1,85	674,55	0,01
MYLK	myosin light chain kinase [Source:NCBI gene;Acc:100547989]	78,41	16,89	2,22	61,52	0,01
MYLK2	myosin light chain kinase 2 [Source:HGNC Symbol;Acc:HGNC:16243]	48,02	9,63	2,32	38,39	0,01
MYMK	myomaker, myoblast fusion factor [Source:HGNC Symbol;Acc:HGNC:33778]	562,77	70,04	3,01	492,73	0,00
MYO16	myosin XVI [Source:HGNC Symbol;Acc:HGNC:29822]	148,30	23,48	2,66	124,83	0,00
MYO18B	myosin XVIIIB [Source:HGNC Symbol;Acc:HGNC:18150]	107,12	27,96	1,94	79,15	0,01
MYO1D	myosin ID [Source:HGNC Symbol;Acc:HGNC:7598]	94,91	198,00	-1,06	103,09	0,04

MYO9A	myosin IXA [Source:HGNC Symbol;Acc:HGNC:7608]	81,23	25,97	1,65	55,26	0,02
MYOC	myocilin [Source:HGNC Symbol;Acc:HGNC:7610]	6,29	125,30	-4,32	119,00	0,00
MYOD1	myogenic differentiation 1 [Source:HGNC Symbol;Acc:HGNC:7611]	865,23	103,37	3,07	761,86	0,00
MYOG	myogenin [Source:HGNC Symbol;Acc:HGNC:7612]	2280,86	408,12	2,48	1872,73	0,01
MYOM1	myomesin 1 [Source:HGNC Symbol;Acc:HGNC:7613]	652,74	135,10	2,27	517,64	0,01
MYOM2	myomesin 2 [Source:HGNC Symbol;Acc:HGNC:7614]	161,11	20,65	2,96	140,46	0,00
MYOM3	myomesin 3 [Source:HGNC Symbol;Acc:HGNC:26679]	575,19	94,02	2,61	481,18	0,00
MYOZ1	myozenin 1 [Source:NCBI gene;Acc:100543368]	96,59	25,41	1,93	71,17	0,01
MYOZ2	myozenin 2 [Source:NCBI gene;Acc:100539396]	395,20	100,16	1,98	295,04	0,01
MYPN	myopalladin [Source:HGNC Symbol;Acc:HGNC:23246]	24,26	7,59	1,68	16,67	0,04
N4BP3	NEDD4 binding protein 3 [Source:NCBI gene;Acc:100549228]	34,62	14,40	1,27	20,22	0,05
NAV3	neuron navigator 3 [Source:HGNC Symbol;Acc:HGNC:15998]	41,24	17,55	1,23	23,69	0,04
NBEAL2	neurobeachin like 2 [Source:HGNC Symbol;Acc:HGNC:31928]	54,86	14,84	1,89	40,02	0,02
NBL1	NBL1, DAN family BMP antagonist [Source:HGNC Symbol;Acc:HGNC:7650]	252,70	97,95	1,37	154,76	0,03
NCALD	neurocalcin delta [Source:NCBI gene;Acc:100548483]	51,60	19,82	1,38	31,78	0,03
NCAM1	neural cell adhesion molecule 1 [Source:HGNC Symbol;Acc:HGNC:7656]	812,32	142,85	2,51	669,47	0,00
NDC80	NDC80 kinetochore complex component [Source:HGNC Symbol;Acc:HGNC:16909]	40,26	10,13	1,99	30,13	0,02
NDRG3	NDRG family member 3 [Source:HGNC Symbol;Acc:HGNC:14462]	148,19	70,15	1,08	78,04	0,04
NDUFA5	NADH:ubiquinone oxidoreductase subunit A5 [Source:HGNC Symbol;Acc:HGNC:7688]	154,27	60,02	1,36	94,25	0,03
NECTIN3	nectin cell adhesion molecule 3 [Source:HGNC Symbol;Acc:HGNC:17664]	65,44	19,21	1,77	46,23	0,02
NET1	neuroepithelial cell transforming 1 [Source:HGNC Symbol;Acc:HGNC:14592]	158,72	60,96	1,38	97,76	0,03
NEURL1	neuralized E3 ubiquitin protein ligase 1 [Source:NCBI gene;Acc:100539867]	54,32	4,93	3,46	49,39	0,00
NEURL1B	neuralized E3 ubiquitin protein ligase 1B [Source:HGNC Symbol;Acc:HGNC:35422]	76,02	17,99	2,08	58,03	0,01
NEXN	nexilin F-actin binding protein [Source:HGNC Symbol;Acc:HGNC:29557]	1572,45	249,27	2,66	1323,18	0,00
NFATC1	nuclear factor of activated T cells 1 [Source:HGNC Symbol;Acc:HGNC:7775]	41,57	93,19	-1,16	51,62	0,04
NFIA	nuclear factor I A [Source:HGNC Symbol;Acc:HGNC:7784]	10,74	30,73	-1,52	19,99	0,04
NHSL1	NHS like 1 [Source:HGNC Symbol;Acc:HGNC:21021]	67,07	18,66	1,85	48,41	0,02
NHSL2	NHS like 2 [Source:HGNC Symbol;Acc:HGNC:33737]	53,83	3,88	3,80	49,95	0,00
NIBAN1	niban apoptosis regulator 1 [Source:HGNC Symbol;Acc:HGNC:16784]	3,74	29,07	-2,96	25,32	0,01
NID2	nidogen 2 [Source:HGNC Symbol;Acc:HGNC:13389]	966,76	84,16	3,52	882,60	0,00
NIN	ninein [Source:HGNC Symbol;Acc:HGNC:14906]	22,47	5,32	2,08	17,15	0,03
NMI	N-myc and STAT interactor [Source:HGNC Symbol;Acc:HGNC:7854]	39,12	16,72	1,23	22,40	0,05
NMU	neuromedin U [Source:HGNC Symbol;Acc:HGNC:7859]	32,12	3,54	3,18	28,58	0,01
NOD1	nucleotide binding oligomerization domain containing 1 [Source:HGNC Symbol;Acc:HGNC:16390]	9,22	32,78	-1,83	23,55	0,03
NPL	N-acetylneuraminate pyruvate lyase [Source:NCBI gene;Acc:100538535]	4,23	17,99	-2,09	13,76	0,05
NPPB	natriuretic peptides A [Source:NCBI gene;Acc:104914182]	81,45	10,46	2,96	70,98	0,00
NPR2	natriuretic peptide receptor 2 [Source:HGNC Symbol;Acc:HGNC:7944]	421,46	136,59	1,63	284,87	0,02
NR4A3	nuclear receptor subfamily 4 group A member 3 [Source:HGNC Symbol;Acc:HGNC:7982]	45,58	14,78	1,62	30,80	0,02
NRAP	nebulin related anchoring protein [Source:HGNC Symbol;Acc:HGNC:7988]	153,24	69,43	1,14	83,81	0,04
NRSN1	neurensin 1 [Source:NCBI gene;Acc:100546700]	5,26	38,81	-2,88	33,55	0,01
NSG1	neuronal vesicle trafficking associated 1 [Source:NCBI gene;Acc:100547061]	58,71	20,21	1,54	38,50	0,02
NSMCE4A	NSE4 homolog A, SMC5-SMC6 complex component [Source:HGNC Symbol;Acc:HGNC:25935]	76,40	32,72	1,22	43,68	0,04
NT5C1A	5'-nucleotidase, cytosolic IA [Source:HGNC Symbol;Acc:HGNC:17819]	39,50	3,99	3,31	35,52	0,01

NT5DC2	5'-nucleotidase domain containing 2 [Source:NCBI gene;Acc:100539982]	125,40	319,81	-1,35	194,41	0,03
NT5E	5'-nucleotidase ecto [Source:HGNC Symbol;Acc:HGNC:8021]	0,11	16,67	-7,26	16,56	0,03
NTN4	netrin 4 [Source:HGNC Symbol;Acc:HGNC:13658]	174,02	53,54	1,70	120,48	0,02
NTRK3	neurotrophic receptor tyrosine kinase 3 [Source:NCBI gene;Acc:100546817]	0,16	13,29	-6,35	13,13	0,05
NUF2	NUF2 component of NDC80 kinetochore complex [Source:HGNC Symbol;Acc:HGNC:14621]	33,59	9,80	1,78	23,79	0,03
NUMB	NUMB endocytic adaptor protein [Source:HGNC Symbol;Acc:HGNC:8060]	197,41	97,23	1,02	100,18	0,05
NUP155	nucleoporin 155 [Source:HGNC Symbol;Acc:HGNC:8063]	91,05	42,25	1,11	48,81	0,04
NXN	nucleoredoxin [Source:HGNC Symbol;Acc:HGNC:18008]	30,93	67,94	-1,14	37,01	0,04
OAF	out at first homolog [Source:HGNC Symbol;Acc:HGNC:28752]	29,25	102,49	-1,81	73,24	0,02
OBSCN	obscurin, cytoskeletal calmodulin and titin-interacting RhoGEF [Source:HGNC Symbol;Acc:HGNC:15719]	69,08	10,35	2,74	58,72	0,00
OBSL1	obscurin like cytoskeletal adaptor 1 [Source:HGNC Symbol;Acc:HGNC:29092]	219,71	38,70	2,51	181,01	0,00
ODC1	ornithine decarboxylase 1 [Source:HGNC Symbol;Acc:HGNC:8109]	166,97	545,21	-1,71	378,25	0,02
OGFOD3	2-oxoglutarate and iron dependent oxygenase domain containing 3 [Source:HGNC Symbol;Acc:HGNC:26174]	41,40	17,77	1,22	23,63	0,04
OGN	osteoglycin [Source:HGNC Symbol;Acc:HGNC:8126]	0,43	546,16	-10,30	545,72	0,00
OIT3	oncoprotein induced transcript 3 [Source:HGNC Symbol;Acc:HGNC:29953]	48,35	13,73	1,82	34,62	0,02
OLFML2A	olfactomedin like 2A [Source:HGNC Symbol;Acc:HGNC:27270]	188,29	43,74	2,11	144,55	0,01
OLFML2B	olfactomedin like 2B [Source:HGNC Symbol;Acc:HGNC:24558]	210,49	541,73	-1,36	331,24	0,03
OPCML	opioid binding protein/cell adhesion molecule like [Source:NCBI gene;Acc:100547810]	14,11	0,50	4,82	13,61	0,04
OPTC	opticin [Source:NCBI gene;Acc:100549406]	34,67	1,66	4,38	33,01	0,01
P3H1	prolyl 3-hydroxylase 1 [Source:HGNC Symbol;Acc:HGNC:19316]	164,69	341,46	-1,05	176,77	0,04
P3H4	prolyl 3-hydroxylase family member 4 (inactive) [Source:HGNC Symbol;Acc:HGNC:16946]	70,27	187,04	-1,41	116,76	0,03
P4HA3	prolyl 4-hydroxylase subunit alpha 3 [Source:HGNC Symbol;Acc:HGNC:30135]	34,46	357,46	-3,37	323,00	0,00
P4HB	prolyl 4-hydroxylase subunit beta [Source:NCBI gene;Acc:100545902]	1389,96	2823,24	-1,02	1433,29	0,05
PACS2	phosphofurin acidic cluster sorting protein 2 [Source:HGNC Symbol;Acc:HGNC:23794]	96,43	30,34	1,67	66,08	0,02
PACSIN3	protein kinase C and casein kinase substrate in neurons 3 [Source:NCBI gene;Acc:100548749]	537,81	166,55	1,69	371,26	0,02
PALM2AKAP2	PALM2 and AKAP2 fusion [Source:HGNC Symbol;Acc:HGNC:33529]	386,19	83,94	2,20	302,25	0,01
PAPSS2	3'-phosphoadenosine 5'-phosphosulfate synthase 2 [Source:HGNC Symbol;Acc:HGNC:8604]	18,78	49,11	-1,39	30,34	0,03
PAQR7	progestin and adipoQ receptor family member 7 [Source:HGNC Symbol;Acc:HGNC:23146]	30,93	11,68	1,40	19,25	0,05
PARVB	parvin beta [Source:HGNC Symbol;Acc:HGNC:14653]	435,57	173,69	1,33	261,88	0,03
PAX7	paired box 7 [Source:HGNC Symbol;Acc:HGNC:8621]	84,54	5,26	4,01	79,28	0,00
PCMT1	protein-L-isoaspartate (D-aspartate) O-methyltransferase [Source:NCBI gene;Acc:100541549]	143,09	62,40	1,20	80,69	0,03
PCOLCE2	procollagen C-endopeptidase enhancer 2 [Source:HGNC Symbol;Acc:HGNC:8739]	33,26	169,15	-2,35	135,89	0,01
PDE3B	phosphodiesterase 3B [Source:HGNC Symbol;Acc:HGNC:8779]	32,45	86,87	-1,42	54,42	0,03
PDE4DIP	phosphodiesterase 4D interacting protein [Source:HGNC Symbol;Acc:HGNC:15580]	930,08	381,21	1,29	548,86	0,03
PDE5A	phosphodiesterase 5A [Source:HGNC Symbol;Acc:HGNC:8784]	25,94	8,03	1,69	17,91	0,04
PDE9A	phosphodiesterase 9A [Source:HGNC Symbol;Acc:HGNC:8795]	41,73	3,77	3,47	37,96	0,00
PDGFA	platelet derived growth factor subunit A [Source:HGNC Symbol;Acc:HGNC:8799]	23,12	6,31	1,87	16,80	0,04
PDGFB	platelet derived growth factor subunit B [Source:HGNC Symbol;Acc:HGNC:8800]	26,21	1,11	4,56	25,10	0,01
PDGFD	platelet derived growth factor D [Source:HGNC Symbol;Acc:HGNC:30620]	397,70	106,92	1,90	290,78	0,01
PDGFRB	platelet derived growth factor receptor beta [Source:HGNC Symbol;Acc:HGNC:8804]	4,40	32,45	-2,88	28,05	0,01
PDK1	pyruvate dehydrogenase kinase 1 [Source:HGNC Symbol;Acc:HGNC:8809]	84,22	30,67	1,46	53,54	0,03
PDK4	pyruvate dehydrogenase kinase 4 [Source:HGNC Symbol;Acc:HGNC:8812]	4,56	41,58	-3,19	37,02	0,01
PDLIM1	PDZ and LIM domain 1 [Source:HGNC Symbol;Acc:HGNC:2067]	740,32	243,01	1,61	497,30	0,02

PDLIM3	PDZ and LIM domain 3 [Source:NCBI gene;Acc:100548391]	587,51	187,81	1,65	399,70	0,02
PENK	proenkephalin [Source:HGNC Symbol;Acc:HGNC:8831]	523,91	53,65	3,29	470,26	0,00
PER3	period circadian regulator 3 [Source:HGNC Symbol;Acc:HGNC:8847]	20,02	4,87	2,04	15,15	0,04
PFKM	phosphofructokinase, muscle [Source:HGNC Symbol;Acc:HGNC:8877]	694,79	215,72	1,69	479,07	0,02
PGF	placental growth factor [Source:HGNC Symbol;Acc:HGNC:8893]	13,51	227,79	-4,08	214,27	0,00
PGLS	6-phosphogluconolactonase [Source:HGNC Symbol;Acc:HGNC:8903]	55,57	20,60	1,43	34,97	0,03
PHKB	phosphorylase kinase regulatory subunit beta [Source:HGNC Symbol;Acc:HGNC:8927]	116,45	46,18	1,33	70,27	0,03
PHLDB2	pleckstrin homology like domain family B member 2 [Source:HGNC Symbol;Acc:HGNC:29573]	147,11	68,05	1,11	79,06	0,04
PHLPP1	PH domain and leucine rich repeat protein phosphatase 1 [Source:HGNC Symbol;Acc:HGNC:20610]	65,77	26,19	1,33	39,58	0,03
PIK3C2B	phosphatidylinositol-4-phosphate 3-kinase catalytic subunit type 2 beta [Source:HGNC Symbol;Acc:HGNC:8972]	138,26	54,32	1,35	83,95	0,03
PIK3CB	phosphatidylinositol-4,5-bisphosphate 3-kinase catalytic subunit beta [Source:HGNC Symbol;Acc:HGNC:8976]	83,13	34,66	1,26	48,47	0,03
PKD1	polycystin 1, transient receptor potential channel interacting [Source:HGNC Symbol;Acc:HGNC:9008]	9,12	27,96	-1,62	18,84	0,04
PKDCC	protein kinase domain containing, cytoplasmic [Source:HGNC Symbol;Acc:HGNC:25123]	4,56	19,43	-2,09	14,88	0,04
PLA2G4A	phospholipase A2 group IVA [Source:HGNC Symbol;Acc:HGNC:9035]	20,13	54,15	-1,43	34,02	0,03
PLBD1	phospholipase B domain containing 1 [Source:HGNC Symbol;Acc:HGNC:26215]	51,93	16,56	1,65	35,37	0,02
PLCD1	phospholipase C delta 1 [Source:HGNC Symbol;Acc:HGNC:9060]	4,94	39,92	-3,02	34,98	0,01
PLEKHH2	pleckstrin homology, MyTH4 and FERM domain containing H2 [Source:HGNC Symbol;Acc:HGNC:30506]	2,82	22,15	-2,97	19,33	0,02
PLEKHN1	pleckstrin homology domain containing N1 [Source:HGNC Symbol;Acc:HGNC:25284]	18,02	2,55	2,82	15,47	0,03
PLK1	polo like kinase 1 [Source:HGNC Symbol;Acc:HGNC:9077]	34,51	13,18	1,39	21,33	0,04
PLOD2	procollagen-lysine,2-oxoglutarate 5-dioxygenase 2 [Source:HGNC Symbol;Acc:HGNC:9082]	438,34	1293,58	-1,56	855,24	0,02
PLPP7	phospholipid phosphatase 7 (inactive) [Source:NCBI gene;Acc:100538597]	32,34	4,93	2,71	27,41	0,01
PLS3	plastin 3 [Source:HGNC Symbol;Acc:HGNC:9091]	1465,28	529,77	1,47	935,51	0,02
PLXDC1	plexin domain containing 1 [Source:HGNC Symbol;Acc:HGNC:20945]	0,76	29,95	-5,30	29,19	0,01
PLXDC2	plexin domain containing 2 [Source:HGNC Symbol;Acc:HGNC:21013]	9,82	30,34	-1,63	20,52	0,03
PLXNA1	plexin A1 [Source:HGNC Symbol;Acc:HGNC:9099]	558,97	175,68	1,67	383,28	0,02
PLXNA2	plexin A2 [Source:HGNC Symbol;Acc:HGNC:9100]	15,03	0,89	4,08	14,15	0,04
PM20D2	peptidase M20 domain containing 2 [Source:HGNC Symbol;Acc:HGNC:21408]	59,80	23,31	1,36	36,49	0,03
PODN	podocan [Source:HGNC Symbol;Acc:HGNC:23174]	15,30	193,35	-3,66	178,04	0,00
POGLUT3	protein O-glucosyltransferase 3 [Source:NCBI gene;Acc:100539901]	59,53	197,78	-1,73	138,25	0,02
POGZ	pogo transposable element derived with ZNF domain [Source:HGNC Symbol;Acc:HGNC:18801]	106,90	52,27	1,03	54,63	0,05
POLDIP2	DNA polymerase delta interacting protein 2 [Source:HGNC Symbol;Acc:HGNC:23781]	136,42	59,96	1,19	76,45	0,03
POMK	protein O-mannose kinase [Source:HGNC Symbol;Acc:HGNC:26267]	16,17	42,80	-1,40	26,63	0,03
POPDC2	popeye domain containing 2 [Source:NCBI gene;Acc:100545001]	275,55	35,77	2,95	239,78	0,00
POSTN	periostin [Source:HGNC Symbol;Acc:HGNC:16953]	214,34	467,75	-1,13	253,41	0,04
PPL	periplakin [Source:HGNC Symbol;Acc:HGNC:9273]	20,13	58,91	-1,55	38,78	0,02
PPM1K	protein phosphatase, Mg2+/Mn2+ dependent 1K [Source:HGNC Symbol;Acc:HGNC:25415]	39,18	7,31	2,42	31,87	0,01
PPP1R12B	protein phosphatase 1 regulatory subunit 12B [Source:NCBI gene;Acc:104914452]	45,09	12,62	1,84	32,47	0,02
PPP1R14C	protein phosphatase 1 regulatory inhibitor subunit 14C [Source:HGNC Symbol;Acc:HGNC:14952]	43,25	10,41	2,05	32,84	0,01
PPP2R2B	protein phosphatase 2 regulatory subunit Bbeta [Source:HGNC Symbol;Acc:HGNC:9305]	8,84	39,20	-2,15	30,36	0,01
PRAG1	PEAK1 related, kinase-activating pseudokinase 1 [Source:HGNC Symbol;Acc:HGNC:25438]	0,81	45,85	-5,82	45,03	0,00
PRELP	proline and arginine rich end leucine rich repeat protein [Source:HGNC Symbol;Acc:HGNC:9357]	161,16	337,64	-1,07	176,48	0,04
PRKAB1	protein kinase AMP-activated non-catalytic subunit beta 1 [Source:HGNC Symbol;Acc:HGNC:9378]	181,78	80,56	1,17	101,22	0,04
PRKCQ	protein kinase C theta [Source:HGNC Symbol;Acc:HGNC:9410]	55,40	20,49	1,44	34,92	0,03

PRRX1	paired related homeobox 1 [Source:HGNC Symbol;Acc:HGNC:9142]	111,78	293,18	-1,39	181,39	0,03
PRRX2	paired related homeobox 2 [Source:NCBI gene;Acc:100547648]	7,05	101,82	-3,85	94,77	0,00
PRSS23	serine protease 23 [Source:NCBI gene;Acc:100550122]	90,67	34,66	1,39	56,01	0,03
PRSS35	serine protease 35 [Source:NCBI gene;Acc:100544541]	55,84	217,49	-1,96	161,65	0,01
PTGES	prostaglandin E synthase [Source:HGNC Symbol;Acc:HGNC:9599]	7,05	162,78	-4,53	155,73	0,00
PTGS2	prostaglandin-endoperoxide synthase 2 [Source:HGNC Symbol;Acc:HGNC:9605]	30,77	279,11	-3,18	248,35	0,00
PTN	pleiotrophin [Source:HGNC Symbol;Acc:HGNC:9630]	0,33	38,54	-6,89	38,21	0,00
PTPN4	protein tyrosine phosphatase non-receptor type 4 [Source:HGNC Symbol;Acc:HGNC:9656]	57,30	25,64	1,16	31,67	0,04
PTPRG	protein tyrosine phosphatase receptor type G [Source:HGNC Symbol;Acc:HGNC:9671]	247,82	122,25	1,02	125,57	0,05
PTPRQ	protein tyrosine phosphatase receptor type Q [Source:HGNC Symbol;Acc:HGNC:9679]	80,53	5,04	4,00	75,49	0,00
PTPRT	protein tyrosine phosphatase receptor type T [Source:HGNC Symbol;Acc:HGNC:9682]	23,22	3,77	2,62	19,46	0,02
RAB32	RAB32, member RAS oncogene family [Source:HGNC Symbol;Acc:HGNC:9772]	7,33	26,80	-1,87	19,47	0,03
RAB3IL1	RAB3A interacting protein like 1 [Source:HGNC Symbol;Acc:HGNC:9780]	26,70	62,62	-1,23	35,92	0,04
RACGAP1	Rac GTPase activating protein 1 [Source:HGNC Symbol;Acc:HGNC:9804]	33,26	7,70	2,11	25,57	0,02
RALGPS2	Ral GEF with PH domain and SH3 binding motif 2 [Source:HGNC Symbol;Acc:HGNC:30279]	307,89	57,69	2,42	250,20	0,01
RAP1GAP2	RAP1 GTPase activating protein 2 [Source:NCBI gene;Acc:100547752]	17,20	2,49	2,79	14,71	0,04
RAPGEF1	Rap guanine nucleotide exchange factor 1 [Source:HGNC Symbol;Acc:HGNC:4568]	203,49	51,05	1,99	152,44	0,01
RAPH1	Ras association (RalGDS/AF-6) and pleckstrin homology domains 1 [Source:HGNC Symbol;Acc:HGNC:14436]	118,19	49,94	1,24	68,24	0,03
RAPSN	receptor associated protein of the synapse [Source:HGNC Symbol;Acc:HGNC:9863]	230,84	24,58	3,23	206,25	0,00
RASA1	RAS p21 protein activator 1 [Source:HGNC Symbol;Acc:HGNC:9871]	72,44	34,00	1,09	38,45	0,04
RASA3	RAS p21 protein activator 3 [Source:NCBI gene;Acc:100547617]	150,58	59,36	1,34	91,23	0,03
RASAL2	RAS protein activator like 2 [Source:HGNC Symbol;Acc:HGNC:9874]	24,42	5,37	2,18	19,05	0,03
RASD1	ras related dexamethasone induced 1 [Source:HGNC Symbol;Acc:HGNC:15828]	2,33	22,42	-3,26	20,09	0,02
RASGEF1B	RasGEF domain family member 1B [Source:HGNC Symbol;Acc:HGNC:24881]	46,29	12,57	1,88	33,72	0,02
RASSF3	Ras association domain family member 3 [Source:HGNC Symbol;Acc:HGNC:14271]	893,12	381,21	1,23	511,91	0,03
RBM20	RNA binding motif protein 20 [Source:HGNC Symbol;Acc:HGNC:27424]	27,95	4,15	2,75	23,79	0,01
RBM24	RNA binding motif protein 24 [Source:HGNC Symbol;Acc:HGNC:21539]	163,82	36,60	2,16	127,22	0,01
RBM38	RNA binding motif protein 38 [Source:HGNC Symbol;Acc:HGNC:15818]	74,18	13,73	2,43	60,45	0,01
RCC1	regulator of chromosome condensation 1 [Source:NCBI gene;Acc:100545702]	123,83	57,20	1,11	66,63	0,04
RDM1	RAD52 motif containing 1 [Source:HGNC Symbol;Acc:HGNC:19950]	38,80	15,50	1,32	23,30	0,04
REEP1	receptor accessory protein 1 [Source:HGNC Symbol;Acc:HGNC:25786]	87,80	24,92	1,82	62,88	0,02
RET	ret proto-oncogene [Source:HGNC Symbol;Acc:HGNC:9967]	11,18	39,26	-1,81	28,08	0,02
RFLNB	refilin B [Source:HGNC Symbol;Acc:HGNC:28705]	408,71	97,84	2,06	310,88	0,01
RGCC	regulator of cell cycle [Source:HGNC Symbol;Acc:HGNC:20369]	1,25	34,66	-4,80	33,41	0,01
RGL1	ral guanine nucleotide dissociation stimulator like 1 [Source:HGNC Symbol;Acc:HGNC:30281]	17,64	85,82	-2,28	68,19	0,01
RGMA	repulsive guidance molecule BMP co-receptor a [Source:HGNC Symbol;Acc:HGNC:30308]	180,15	66,05	1,45	114,10	0,02
RGN	regucalcin [Source:HGNC Symbol;Acc:HGNC:9989]	21,76	58,64	-1,43	36,88	0,03
RHBDF1	rhomboid 5 homolog 1 [Source:HGNC Symbol;Acc:HGNC:20561]	189,38	76,35	1,31	113,03	0,03
RHOB	ras homolog family member B [Source:NCBI gene;Acc:100542519]	1352,95	562,05	1,27	790,90	0,03
RNF20	ring finger protein 20 [Source:HGNC Symbol;Acc:HGNC:10062]	278,64	105,03	1,41	173,61	0,03
RNF208	ring finger protein 208 [Source:HGNC Symbol;Acc:HGNC:25420]	18,61	4,04	2,20	14,57	0,04
ROR1	receptor tyrosine kinase like orphan receptor 1 [Source:NCBI gene;Acc:100551116]	8,57	76,91	-3,17	68,33	0,00
RPA2	replication protein A2 [Source:NCBI gene;Acc:100542097]	105,16	49,28	1,09	55,88	0,04

RPL10A	ribosomal protein L10a [Source:NCBI gene;Acc:100544053]	379,63	811,09	-1,10	431,47	0,04
RPL11	ribosomal protein L11 [Source:HGNC Symbol;Acc:HGNC:10301]	407,25	927,04	-1,19	519,79	0,03
RPL12	ribosomal protein L12 [Source:HGNC Symbol;Acc:HGNC:10302]	261,39	558,39	-1,10	297,01	0,04
RPL22L1	ribosomal protein L22 like 1 [Source:HGNC Symbol;Acc:HGNC:27610]	31,04	118,99	-1,94	87,95	0,01
RPL27A	ribosomal protein L27a [Source:HGNC Symbol;Acc:HGNC:10329]	340,72	712,32	-1,06	371,60	0,04
RPL30	ribosomal protein L30 [Source:HGNC Symbol;Acc:HGNC:10333]	223,57	485,69	-1,12	262,13	0,04
RPL31	ribosomal protein L31 [Source:HGNC Symbol;Acc:HGNC:10334]	215,91	448,82	-1,06	232,90	0,04
RPS15A	ribosomal protein S15a [Source:NCBI gene;Acc:100543844]	222,86	558,78	-1,33	335,92	0,03
RPS16	ribosomal protein S16 [Source:HGNC Symbol;Acc:HGNC:10396]	234,36	513,16	-1,13	278,79	0,04
RPS20	ribosomal protein S20 [Source:HGNC Symbol;Acc:HGNC:10405]	396,50	872,44	-1,14	475,94	0,04
RPS21	ribosomal protein S21 [Source:NCBI gene;Acc:100543583]	82,75	173,91	-1,07	91,16	0,04
RPS8	ribosomal protein S8 [Source:HGNC Symbol;Acc:HGNC:10441]	734,89	1531,66	-1,06	796,77	0,04
RRBP1	ribosome binding protein 1 [Source:HGNC Symbol;Acc:HGNC:10448]	276,04	609,89	-1,14	333,85	0,04
RSPO3	R-spondin 3 [Source:HGNC Symbol;Acc:HGNC:20866]	3,74	19,71	-2,40	15,97	0,04
RSPO4	R-spondin 4 [Source:HGNC Symbol;Acc:HGNC:16175]	1,36	23,03	-4,09	21,68	0,02
RUNX1T1	RUNX1 partner transcriptional co-repressor 1 [Source:HGNC Symbol;Acc:HGNC:1535]	6,73	28,07	-2,06	21,34	0,02
RUSC2	RUN and SH3 domain containing 2 [Source:HGNC Symbol;Acc:HGNC:23625]	140,92	30,12	2,23	110,80	0,01
RYR3	ryanodine receptor 3 [Source:HGNC Symbol;Acc:HGNC:10485]	58,12	12,29	2,24	45,82	0,01
S100A11	S100 calcium binding protein A11 [Source:HGNC Symbol;Acc:HGNC:10488]	225,41	511,22	-1,18	285,81	0,03
S100A4	S100 calcium binding protein A4 [Source:HGNC Symbol;Acc:HGNC:10494]	47,86	100,49	-1,07	52,63	0,05
SACS	sacsin molecular chaperone [Source:HGNC Symbol;Acc:HGNC:10519]	144,77	42,63	1,76	102,14	0,02
SALL1	spalt like transcription factor 1 [Source:HGNC Symbol;Acc:HGNC:10524]	43,79	7,42	2,56	36,37	0,01
SAMD11	sterile alpha motif domain containing 11 [Source:HGNC Symbol;Acc:HGNC:28706]	132,24	28,96	2,19	103,28	0,01
SAMD4A	sterile alpha motif domain containing 4A [Source:HGNC Symbol;Acc:HGNC:23023]	310,66	141,02	1,14	169,63	0,04
SARDH	sarcosine dehydrogenase [Source:HGNC Symbol;Acc:HGNC:10536]	449,84	44,74	3,33	405,11	0,00
SCARA3	scavenger receptor class A member 3 [Source:HGNC Symbol;Acc:HGNC:19000]	0,03	101,21	-11,87	101,19	0,00
SCARA5	scavenger receptor class A member 5 [Source:HGNC Symbol;Acc:HGNC:28701]	3,09	35,77	-3,53	32,68	0,01
SCARB1	scavenger receptor class B member 1 [Source:NCBI gene;Acc:100550769]	58,28	142,68	-1,29	84,41	0,03
SCD	stearoyl-CoA desaturase [Source:NCBI gene;Acc:100547172]	183,68	41,14	2,16	142,54	0,01
SCD5	stearoyl-CoA desaturase 5 [Source:HGNC Symbol;Acc:HGNC:21088]	61,59	13,45	2,19	48,13	0,01
SCG3	secretogranin III [Source:HGNC Symbol;Acc:HGNC:13707]	32,40	11,24	1,53	21,16	0,04
SCG5	secretogranin V [Source:HGNC Symbol;Acc:HGNC:10816]	4,18	24,97	-2,58	20,79	0,02
SCIN	scinderin [Source:HGNC Symbol;Acc:HGNC:21695]	0,11	24,97	-7,85	24,86	0,01
SCN4A	sodium voltage-gated channel alpha subunit 4 [Source:HGNC Symbol;Acc:HGNC:10591]	26,32	2,55	3,37	23,77	0,01
SCN4B	sodium voltage-gated channel beta subunit 4 [Source:HGNC Symbol;Acc:HGNC:10592]	7,60	29,95	-1,98	22,36	0,03
SCNN1A	sodium channel epithelial 1 alpha subunit [Source:NCBI gene;Acc:100546783]	29,52	10,35	1,51	19,17	0,04
SCRIB	scribble planar cell polarity protein [Source:HGNC Symbol;Acc:HGNC:30377]	146,62	37,65	1,96	108,97	0,01
SDC1	syndecan 1 [Source:HGNC Symbol;Acc:HGNC:10658]	62,29	146,39	-1,23	84,10	0,03
SDC2	syndecan 2 [Source:HGNC Symbol;Acc:HGNC:10659]	92,41	244,62	-1,40	152,21	0,03
SDHAF4	succinate dehydrogenase complex assembly factor 4 [Source:HGNC Symbol;Acc:HGNC:20957]	42,00	18,99	1,15	23,01	0,05
SDK2	sidekick cell adhesion molecule 2 [Source:HGNC Symbol;Acc:HGNC:19308]	683,56	201,21	1,76	482,35	0,02
SEC24D	SEC24 homolog D, COPII coat complex component [Source:NCBI gene;Acc:100540588]	87,85	179,67	-1,03	91,82	0,05

SEMA3B	semaphorin 3B [Source:HGNC Symbol;Acc:HGNC:10724]	8,09	32,72	-2,02	24,64	0,02
SERHL2	serine hydrolase like 2 [Source:NCBI gene;Acc:100549399]	15,03	40,03	-1,41	25,00	0,04
SERINC1	serine incorporator 1 [Source:NCBI gene;Acc:100548079]	1391,32	637,02	1,13	754,30	0,04
SERINC2	serine incorporator 2 [Source:HGNC Symbol;Acc:HGNC:23231]	7,98	29,40	-1,88	21,42	0,03
SFRP2	secreted frizzled related protein 2 [Source:HGNC Symbol;Acc:HGNC:10777]	393,63	121,81	1,69	271,82	0,02
SFRP4	secreted frizzled related protein 4 [Source:HGNC Symbol;Acc:HGNC:10778]	13,62	58,64	-2,11	45,02	0,01
SGCG	sarcoglycan gamma [Source:HGNC Symbol;Acc:HGNC:10809]	102,56	26,96	1,93	75,59	0,01
SH2B2	SH2B adaptor protein 2 [Source:HGNC Symbol;Acc:HGNC:17381]	23,71	6,98	1,77	16,74	0,04
SH2D3C	SH2 domain containing 3C [Source:HGNC Symbol;Acc:HGNC:16884]	195,57	23,14	3,08	172,42	0,00
SH3BGRL	SH3 domain binding glutamate rich protein like [Source:NCBI gene;Acc:100541924]	116,07	44,90	1,37	71,17	0,03
SH3PXD2B	SH3 and PX domains 2B [Source:HGNC Symbol;Acc:HGNC:29242]	22,63	101,38	-2,16	78,75	0,01
SHC1	SHC adaptor protein 1 [Source:HGNC Symbol;Acc:HGNC:10840]	113,46	266,05	-1,23	152,58	0,03
SHISA2	shisa family member 2 [Source:HGNC Symbol;Acc:HGNC:20366]	571,07	224,46	1,35	346,60	0,03
SHROOM3	shroom family member 3 [Source:HGNC Symbol;Acc:HGNC:30422]	138,43	13,79	3,33	124,64	0,00
SIM2	SIM bHLH transcription factor 2 [Source:HGNC Symbol;Acc:HGNC:10883]	72,28	12,57	2,52	59,71	0,01
SIPA1L2	signal induced proliferation associated 1 like 2 [Source:HGNC Symbol;Acc:HGNC:23800]	52,42	11,30	2,21	41,12	0,01
SIX4	SIX homeobox 4 [Source:NCBI gene;Acc:100545734]	146,95	22,20	2,73	124,74	0,00
SLC16A2	solute carrier family 16 member 2 [Source:HGNC Symbol;Acc:HGNC:10923]	23,60	50,16	-1,09	26,56	0,05
SLC16A3	solute carrier family 16 member 3 [Source:NCBI gene;Acc:100542493]	164,69	337,36	-1,03	172,67	0,05
SLC1A6	solute carrier family 1 member 6 [Source:HGNC Symbol;Acc:HGNC:10944]	0,05	16,67	-8,26	16,61	0,03
SLC20A2	solute carrier family 20 member 2 [Source:NCBI gene;Acc:100539038]	101,36	44,41	1,19	56,96	0,04
SLC25A4	solute carrier family 25 member 4 [Source:HGNC Symbol;Acc:HGNC:10990]	156,33	51,88	1,59	104,45	0,02
SLC26A5	solute carrier family 26 member 5 [Source:HGNC Symbol;Acc:HGNC:9359]	22,25	53,04	-1,25	30,80	0,04
SLC37A2	solute carrier family 37 member 2 [Source:HGNC Symbol;Acc:HGNC:20644]	14,87	43,69	-1,55	28,82	0,03
SLC43A2	solute carrier family 43 member 2 [Source:HGNC Symbol;Acc:HGNC:23087]	5,91	32,17	-2,44	26,25	0,01
SLC44A3	solute carrier family 44 member 3 [Source:HGNC Symbol;Acc:HGNC:28689]	2,06	21,59	-3,39	19,53	0,02
SLC8A3	solute carrier family 8 member A3 [Source:HGNC Symbol;Acc:HGNC:11070]	19,97	2,93	2,77	17,03	0,03
SLC9A7	solute carrier family 9 member A7 [Source:HGNC Symbol;Acc:HGNC:17123]	46,99	14,34	1,71	32,65	0,02
SLIT1	slit guidance ligand 1 [Source:HGNC Symbol;Acc:HGNC:11085]	504,98	28,24	4,16	476,74	0,00
SMARCD2	SWI/SNF related, matrix associated, actin dependent regulator of chromatin, subfamily d, member 2 [Source:HGNC Symbol;Acc:HGNC:11107]	55,40	115,22	-1,06	59,82	0,05
SMTN	smoothelin [Source:HGNC Symbol;Acc:HGNC:11126]	448,43	112,68	1,99	335,76	0,01
SMYD1	SET and MYND domain containing 1 [Source:NCBI gene;Acc:100548953]	458,80	90,20	2,35	368,60	0,01
SMYD2	SET and MYND domain containing 2 [Source:NCBI gene;Acc:100538978]	66,53	27,08	1,30	39,45	0,03
SNAI1	snail family transcriptional repressor 1 [Source:NCBI gene;Acc:100542762]	74,94	30,73	1,29	44,21	0,03
SNX21	sorting nexin family member 21 [Source:HGNC Symbol;Acc:HGNC:16154]	136,74	44,96	1,60	91,78	0,02
SNX30	sorting nexin family member 30 [Source:HGNC Symbol;Acc:HGNC:23685]	6,29	24,09	-1,94	17,79	0,04
SNX5	sorting nexin 5 [Source:HGNC Symbol;Acc:HGNC:14969]	10,36	64,39	-2,64	54,03	0,01
SNX7	sorting nexin 7 [Source:HGNC Symbol;Acc:HGNC:14971]	68,75	149,22	-1,12	80,47	0,04
SOD3	superoxide dismutase 3 [Source:HGNC Symbol;Acc:HGNC:11181]	9,82	32,50	-1,73	22,68	0,03
SORBS1	sorbin and SH3 domain containing 1 [Source:HGNC Symbol;Acc:HGNC:14565]	109,29	39,48	1,47	69,81	0,02
SOX8	SRY-box transcription factor 8 [Source:HGNC Symbol;Acc:HGNC:11203]	81,99	16,00	2,36	65,99	0,01
SOX9	SRY-box transcription factor 9 [Source:HGNC Symbol;Acc:HGNC:11204]	6,19	26,13	-2,08	19,95	0,03
SPARC	secreted protein acidic and cysteine rich [Source:HGNC Symbol;Acc:HGNC:11219]	3094,16	6930,38	-1,16	3836,22	0,04

SPART	spartin [Source:HGNC Symbol;Acc:HGNC:18514]	299,70	83,27	1,85	216,42	0,01
SPECC1	sperm antigen with calponin homology and coiled-coil domains 1 [Source:HGNC Symbol;Acc:HGNC:30615]	364,32	84,38	2,11	279,94	0,01
SPON1	spondin 1 [Source:HGNC Symbol;Acc:HGNC:11252]	24,09	59,36	-1,30	35,26	0,03
SPON2	spondin 2 [Source:HGNC Symbol;Acc:HGNC:11253]	0,54	377,28	-9,44	376,74	0,00
SPP1	secreted phosphoprotein 1 [Source:NCBI gene;Acc:100541448]	45,15	137,31	-1,60	92,17	0,02
SPRED1	sprouty related EVH1 domain containing 1 [Source:HGNC Symbol;Acc:HGNC:20249]	33,43	75,80	-1,18	42,37	0,04
SPRY2	sprouty RTK signaling antagonist 2 [Source:NCBI gene;Acc:100545563]	81,23	32,72	1,31	48,51	0,03
SPTAN1	spectrin alpha, non-erythrocytic 1 [Source:NCBI gene;Acc:100545390]	655,83	314,99	1,06	340,84	0,04
SPTBN1	spectrin beta, non-erythrocytic 1 [Source:NCBI gene;Acc:100539592]	1191,25	454,69	1,39	736,56	0,03
SRF	serum response factor [Source:HGNC Symbol;Acc:HGNC:11291]	144,67	63,73	1,18	80,94	0,04
SRL	sarcalumenin [Source:HGNC Symbol;Acc:HGNC:11295]	393,36	90,80	2,11	302,55	0,01
SRM	spermidine synthase [Source:HGNC Symbol;Acc:HGNC:11296]	13,84	40,81	-1,56	26,97	0,03
SRPX	sushi repeat containing protein X-linked [Source:HGNC Symbol;Acc:HGNC:11309]	20,95	59,47	-1,51	38,52	0,03
SSPN	sarcospan [Source:NCBI gene;Acc:100540204]	117,32	251,04	-1,10	133,72	0,04
STAB1	stabilin 1 [Source:HGNC Symbol;Acc:HGNC:18628]	0,87	24,20	-4,80	23,33	0,01
STARD8	StAR related lipid transfer domain containing 8 [Source:HGNC Symbol;Acc:HGNC:19161]	98,54	35,60	1,47	62,94	0,02
STC2	stanniocalcin 2 [Source:HGNC Symbol;Acc:HGNC:11374]	107,44	23,14	2,21	84,30	0,01
STEAP3	STEAP3 metalloreductase [Source:HGNC Symbol;Acc:HGNC:24592]	67,12	12,18	2,46	54,94	0,01
STK17A	serine/threonine kinase 17a [Source:HGNC Symbol;Acc:HGNC:11395]	353,69	109,85	1,69	243,84	0,02
STOM	stomatin [Source:HGNC Symbol;Acc:HGNC:3383]	51,17	16,72	1,61	34,45	0,02
STXBP5	syntaxin binding protein 5 [Source:HGNC Symbol;Acc:HGNC:19665]	11,67	35,55	-1,61	23,88	0,03
STYXL2	dual specificity phosphatase 27, atypical [Source:NCBI gene;Acc:100543131]	38,04	7,36	2,37	30,67	0,01
SUCO	SUN domain containing ossification factor [Source:HGNC Symbol;Acc:HGNC:1240]	58,55	27,96	1,07	30,59	0,05
SULF1	sulfatase 1 [Source:HGNC Symbol;Acc:HGNC:20391]	5,37	149,94	-4,80	144,57	0,00
SVIL	supervillin [Source:HGNC Symbol;Acc:HGNC:11480]	579,10	266,16	1,12	312,94	0,04
SYBU	syntabulin [Source:HGNC Symbol;Acc:HGNC:26011]	2,93	19,77	-2,75	16,84	0,03
SYNC	syncoilin, intermediate filament protein [Source:HGNC Symbol;Acc:HGNC:28897]	172,29	45,85	1,91	126,44	0,01
SYNE2	spectrin repeat containing nuclear envelope protein 2 [Source:HGNC Symbol;Acc:HGNC:17084]	167,35	59,91	1,48	107,44	0,02
SYNJ2	synaptojanin 2 [Source:HGNC Symbol;Acc:HGNC:11504]	181,84	54,32	1,74	127,52	0,02
SYNM	synemin [Source:HGNC Symbol;Acc:HGNC:24466]	29,19	7,97	1,87	21,22	0,03
SYNPO	synaptopodin [Source:HGNC Symbol;Acc:HGNC:30672]	217,16	66,05	1,72	151,11	0,02
SYNPO2	synaptopodin 2 [Source:HGNC Symbol;Acc:HGNC:17732]	216,62	29,18	2,89	187,44	0,00
SYNPO2L	synaptopodin 2 like [Source:HGNC Symbol;Acc:HGNC:23532]	55,51	10,63	2,38	44,88	0,01
SYT12	synaptotagmin 12 [Source:HGNC Symbol;Acc:HGNC:18381]	0,60	15,28	-4,68	14,68	0,04
SYTL4	synaptotagmin like 4 [Source:HGNC Symbol;Acc:HGNC:15588]	13,13	33,50	-1,35	20,37	0,04
TACC1	transforming acidic coiled-coil containing protein 1 [Source:HGNC Symbol;Acc:HGNC:11522]	215,59	70,48	1,61	145,10	0,02
TACC2	transforming acidic coiled-coil containing protein 2 [Source:HGNC Symbol;Acc:HGNC:11523]	1174,26	267,71	2,13	906,55	0,01
TAOK3	TAO kinase 3 [Source:HGNC Symbol;Acc:HGNC:18133]	187,97	79,51	1,24	108,46	0,03
TBX15	T-box transcription factor 15 [Source:HGNC Symbol;Acc:HGNC:11594]	44,88	12,85	1,80	32,03	0,02
TBX18	T-box transcription factor 18 [Source:HGNC Symbol;Acc:HGNC:11595]	1,57	19,38	-3,62	17,81	0,02
TEAD3	TEA domain transcription factor 3 [Source:HGNC Symbol;Acc:HGNC:11716]	63,49	24,75	1,36	38,74	0,03
TFEB	transcription factor EB [Source:NCBI gene;Acc:100545910]	209,40	76,19	1,46	133,22	0,02
TGFBI	transforming growth factor beta induced [Source:NCBI gene;Acc:100539931]	565,43	4669,73	-3,05	4104,31	0,00

TGFBR2	transforming growth factor beta receptor 2 [Source:HGNC Symbol;Acc:HGNC:11773]	17,53	52,32	-1,58	34,80	0,02
THBS2	thrombospondin 2 [Source:HGNC Symbol;Acc:HGNC:11786]	3,74	144,18	-5,27	140,44	0,00
THBS4	thrombospondin 4 [Source:HGNC Symbol;Acc:HGNC:11788]	29,79	987,55	-5,05	957,76	0,00
THY1	Thy-1 cell surface antigen [Source:NCBI gene;Acc:100538807]	273,76	768,68	-1,49	494,92	0,02
THYN1	thymocyte nuclear protein 1 [Source:NCBI gene;Acc:100540352]	145,86	71,59	1,03	74,27	0,05
TIMP3	TIMP metallopeptidase inhibitor 3 [Source:NCBI gene;Acc:100539796]	1143,01	337,03	1,76	805,98	0,02
TJP1	tight junction protein 1 [Source:HGNC Symbol;Acc:HGNC:11827]	120,14	51,83	1,21	68,31	0,03
TKT	transketolase [Source:NCBI gene;Acc:100545441]	30,98	105,26	-1,76	74,27	0,02
TLCD3A	TLC domain containing 3A [Source:HGNC Symbol;Acc:HGNC:29646]	261,28	85,54	1,61	175,73	0,02
TLN2	talin 2 [Source:HGNC Symbol;Acc:HGNC:15447]	51,22	19,05	1,43	32,18	0,03
TM4SF18	transmembrane 4 L six family member 18 [Source:HGNC Symbol;Acc:HGNC:25181]	29,19	9,63	1,60	19,56	0,04
TMEFF2	transmembrane protein with EGF like and two follistatin like domains 2 [Source:HGNC Symbol;Acc:HGNC:11867]	19,48	4,98	1,97	14,50	0,05
TMEM116	transmembrane protein 116 [Source:NCBI gene;Acc:100547281]	17,26	66,50	-1,95	49,24	0,01
TMEM119	transmembrane protein 119 [Source:HGNC Symbol;Acc:HGNC:27884]	2,06	93,52	-5,50	91,46	0,00
TMEM132C	transmembrane protein 132C [Source:HGNC Symbol;Acc:HGNC:25436]	1,79	47,23	-4,72	45,44	0,00
TMEM19	transmembrane protein 19 [Source:HGNC Symbol;Acc:HGNC:25605]	47,86	18,05	1,41	29,81	0,03
TMEM200A	transmembrane protein 200A [Source:NCBI gene;Acc:100543662]	102,72	47,84	1,10	54,88	0,04
TMEM204	transmembrane protein 204 [Source:HGNC Symbol;Acc:HGNC:14158]	3,91	49,17	-3,65	45,26	0,00
TMEM268	transmembrane protein 268 [Source:NCBI gene;Acc:100540089]	1,41	39,92	-4,82	38,51	0,00
TMEM38A	transmembrane protein 38A [Source:NCBI gene;Acc:100539425]	294,11	81,28	1,86	212,83	0,01
TMEM47	transmembrane protein 47 [Source:HGNC Symbol;Acc:HGNC:18515]	49,38	8,80	2,49	40,58	0,01
TMEM64	transmembrane protein 64 [Source:HGNC Symbol;Acc:HGNC:25441]	107,98	35,21	1,62	72,77	0,02
TMEM9	transmembrane protein 9 [Source:HGNC Symbol;Acc:HGNC:18823]	22,74	4,26	2,41	18,47	0,03
TMTC2	transmembrane O-mannosyltransferase targeting cadherins 2 [Source:HGNC Symbol;Acc:HGNC:25440]	2,44	17,66	-2,85	15,22	0,04
TNFAIP8L3	TNF alpha induced protein 8 like 3 [Source:HGNC Symbol;Acc:HGNC:20620]	42,49	10,30	2,04	32,19	0,02
TNFRSF19	TNF receptor superfamily member 19 [Source:HGNC Symbol;Acc:HGNC:11915]	23,28	3,65	2,67	19,62	0,02
TNFRSF6B	TNF receptor superfamily member 6b [Source:HGNC Symbol;Acc:HGNC:11921]	22,25	66,17	-1,57	43,92	0,02
TNKS1BP1	tankyrase 1 binding protein 1 [Source:HGNC Symbol;Acc:HGNC:19081]	55,08	199,05	-1,85	143,97	0,01
TNNC1	troponin C1, slow skeletal and cardiac type [Source:HGNC Symbol;Acc:HGNC:11943]	858,23	197,39	2,12	660,84	0,01
TNNC2	troponin C2, fast skeletal type [Source:NCBI gene;Acc:100544678]	558,10	87,32	2,68	470,78	0,00
TNNI1	troponin I1, slow skeletal type [Source:HGNC Symbol;Acc:HGNC:11945]	1720,59	514,32	1,74	1206,27	0,02
TNNI2	troponin I2, fast skeletal type [Source:HGNC Symbol;Acc:HGNC:11946]	1221,69	346,50	1,82	875,19	0,01
TNNT2	troponin T2, cardiac type [Source:HGNC Symbol;Acc:HGNC:11949]	5088,61	1104,27	2,20	3984,34	0,01
TNNT3	troponin T3, fast skeletal type [Source:HGNC Symbol;Acc:HGNC:11950]	3029,53	748,80	2,02	2280,72	0,01
TOMM70	translocase of outer mitochondrial membrane 70 [Source:HGNC Symbol;Acc:HGNC:11985]	132,67	62,40	1,09	70,27	0,04
TOP2A	DNA topoisomerase II alpha [Source:HGNC Symbol;Acc:HGNC:11989]	136,31	55,76	1,29	80,55	0,03
TOR2A	torsin family 2 member A [Source:HGNC Symbol;Acc:HGNC:11996]	8,63	25,64	-1,57	17,01	0,05
TPM1	tropomyosin 1 [Source:HGNC Symbol;Acc:HGNC:12010]	6335,96	2497,51	1,34	3838,45	0,03
TPST2	tyrosylprotein sulfotransferase 2 [Source:NCBI gene;Acc:100542914]	35,98	15,34	1,23	20,64	0,05
TRAK1	trafficking kinesin protein 1 [Source:HGNC Symbol;Acc:HGNC:29947]	92,30	38,54	1,26	53,77	0,03
TRIM45	tripartite motif containing 45 [Source:HGNC Symbol;Acc:HGNC:19018]	83,78	25,47	1,72	58,31	0,02
TRIM54	tripartite motif containing 54 [Source:HGNC Symbol;Acc:HGNC:16008]	187,75	48,84	1,94	138,92	0,01
TRIM55	tripartite motif containing 55 [Source:HGNC Symbol;Acc:HGNC:14215]	443,93	91,03	2,29	352,90	0,01

TRIM63	tripartite motif containing 63 [Source:HGNC Symbol;Acc:HGNC:16007]	114,28	33,94	1,75	80,34	0,02
TRIM8	tripartite motif containing 8 [Source:HGNC Symbol;Acc:HGNC:15579]	373,17	158,80	1,23	214,37	0,03
TRPV2	transient receptor potential cation channel subfamily V member 2 [Source:NCBI gene;Acc:100540299]	284,39	82,39	1,79	202,01	0,02
TSC22D3	TSC22 domain family member 3 [Source:HGNC Symbol;Acc:HGNC:3051]	88,78	36,65	1,28	52,12	0,03
TSHZ2	teashirt zinc finger homeobox 2 [Source:HGNC Symbol;Acc:HGNC:13010]	11,23	83,16	-2,89	71,93	0,00
TSPAN12	tetraspanin 12 [Source:HGNC Symbol;Acc:HGNC:21641]	252,00	61,63	2,03	190,37	0,01
TSPAN6	tetraspanin 6 [Source:HGNC Symbol;Acc:HGNC:11858]	77,22	205,69	-1,41	128,48	0,03
TTC29	tetratricopeptide repeat domain 29 [Source:HGNC Symbol;Acc:HGNC:29936]	0,16	67,94	-8,71	67,77	0,00
TTC9	tetratricopeptide repeat domain 9 [Source:HGNC Symbol;Acc:HGNC:20267]	93,28	28,02	1,74	65,26	0,02
TUBB6	tubulin beta 6 class V [Source:HGNC Symbol;Acc:HGNC:20776]	769,46	308,46	1,32	461,00	0,03
TUFT1	tuftelin 1 [Source:HGNC Symbol;Acc:HGNC:12422]	141,52	59,02	1,26	82,50	0,03
TWF2	twinfilin actin binding protein 2 [Source:HGNC Symbol;Acc:HGNC:9621]	737,33	296,89	1,31	440,45	0,03
TWIST2	twist family bHLH transcription factor 2 [Source:NCBI gene;Acc:100547115]	0,92	26,13	-4,82	25,21	0,01
UAP1	UDP-N-acetylglucosamine pyrophosphorylase 1 [Source:HGNC Symbol;Acc:HGNC:12457]	44,88	194,34	-2,11	149,47	0,01
UAP1L1	UDP-N-acetylglucosamine pyrophosphorylase 1 like 1 [Source:HGNC Symbol;Acc:HGNC:28082]	7,76	50,88	-2,71	43,12	0,01
UBXN4	UBX domain protein 4 [Source:HGNC Symbol;Acc:HGNC:14860]	567,05	265,16	1,10	301,89	0,04
UCHL3	ubiquitin C-terminal hydrolase L3 [Source:NCBI gene;Acc:100548021]	97,62	43,91	1,15	53,71	0,04
UCK1	uridine-cytidine kinase 1 [Source:HGNC Symbol;Acc:HGNC:14859]	144,45	53,10	1,44	91,35	0,02
UGDH	UDP-glucose 6-dehydrogenase [Source:NCBI gene;Acc:100549207]	95,50	326,45	-1,77	230,95	0,02
UNC45B	unc-45 myosin chaperone B [Source:HGNC Symbol;Acc:HGNC:14304]	302,46	86,82	1,80	215,65	0,02
UNC5A	unc-5 netrin receptor A [Source:HGNC Symbol;Acc:HGNC:12567]	45,85	14,12	1,70	31,73	0,02
USP13	ubiquitin specific peptidase 13 [Source:HGNC Symbol;Acc:HGNC:12611]	39,50	9,86	2,00	29,65	0,02
USP25	ubiquitin specific peptidase 25 [Source:HGNC Symbol;Acc:HGNC:12624]	167,24	80,78	1,05	86,46	0,05
USP28	ubiquitin specific peptidase 28 [Source:HGNC Symbol;Acc:HGNC:12625]	120,30	45,35	1,41	74,96	0,03
USP6NL	USP6 N-terminal like [Source:NCBI gene;Acc:100538767]	62,57	25,69	1,28	36,87	0,03
VAC14	VAC14 component of PIKFYVE complex [Source:HGNC Symbol;Acc:HGNC:25507]	125,35	60,02	1,06	65,33	0,05
VASH2	vasohibin 2 [Source:HGNC Symbol;Acc:HGNC:25723]	977,83	174,47	2,49	803,36	0,00
VAV2	vav guanine nucleotide exchange factor 2 [Source:HGNC Symbol;Acc:HGNC:12658]	164,69	52,10	1,66	112,59	0,02
VDR	vitamin D receptor [Source:HGNC Symbol;Acc:HGNC:12679]	2,93	26,47	-3,18	23,54	0,01
VEGFA	vascular endothelial growth factor A [Source:HGNC Symbol;Acc:HGNC:12680]	29,57	98,06	-1,73	68,48	0,02
VEGFC	vascular endothelial growth factor C [Source:HGNC Symbol;Acc:HGNC:12682]	39,72	13,79	1,53	25,93	0,03
VEPH1	ventricular zone expressed PH domain containing 1 [Source:HGNC Symbol;Acc:HGNC:25735]	57,36	13,23	2,12	44,12	0,01
VSTM2L	V-set and transmembrane domain containing 2 like [Source:HGNC Symbol;Acc:HGNC:16096]	34,19	4,65	2,88	29,54	0,01
WDSUB1	WD repeat, sterile alpha motif and U-box domain containing 1 [Source:HGNC Symbol;Acc:HGNC:26697]	13,78	41,86	-1,60	28,08	0,03
WIPF3	WAS/WASL interacting protein family member 3 [Source:HGNC Symbol;Acc:HGNC:22004]	100,77	16,33	2,63	84,43	0,00
WNT7A	Wnt family member 7A [Source:NCBI gene;Acc:100542958]	17,80	1,66	3,42	16,14	0,03
WWC1	WW and C2 domain containing 1 [Source:NCBI gene;Acc:100540762]	34,51	11,63	1,57	22,88	0,03
XIRP1	xin actin binding repeat containing 1 [Source:NCBI gene;Acc:100541459]	1448,83	227,73	2,67	1221,10	0,00
XPR1	xenotropic and polytropic retrovirus receptor 1 [Source:HGNC Symbol;Acc:HGNC:12827]	174,08	65,11	1,42	108,96	0,03
YDJC	YdjC chitooligosaccharide deacetylase homolog [Source:HGNC Symbol;Acc:HGNC:27158]	46,67	10,85	2,10	35,81	0,01
YIPF5	Yip1 domain family member 5 [Source:HGNC Symbol;Acc:HGNC:24877]	104,35	260,56	-1,32	156,22	0,03
ZBTB18	zinc finger and BTB domain containing 18 [Source:HGNC Symbol;Acc:HGNC:13030]	76,78	27,91	1,46	48,88	0,03
ZEB1	zinc finger E-box binding homeobox 1 [Source:HGNC Symbol;Acc:HGNC:11642]	470,90	224,08	1,07	246,82	0,04

ZFAND2A	zinc finger AN1-type containing 2A [Source:NCBI gene;Acc:100547491]	19,48	43,52	-1,16	24,04	0,05
ZFAND5	zinc finger AN1-type containing 5 [Source:HGNC Symbol;Acc:HGNC:13008]	53,34	19,10	1,48	34,24	0,03
ZFYVE9	zinc finger FYVE-type containing 9 [Source:NCBI gene;Acc:100545376]	1,79	34,11	-4,25	32,32	0,01
ZHX2	zinc fingers and homeoboxes 2 [Source:HGNC Symbol;Acc:HGNC:18513]	46,12	13,73	1,75	32,39	0,02
ZNF106	zinc finger protein 106 [Source:HGNC Symbol;Acc:HGNC:12886]	260,84	42,63	2,61	218,21	0,00
ZNF385B	zinc finger protein 385B [Source:HGNC Symbol;Acc:HGNC:26332]	85,30	21,32	2,00	63,99	0,01
ZNF423	zinc finger protein 423 [Source:HGNC Symbol;Acc:HGNC:16762]	20,67	57,69	-1,48	37,02	0,03
ZNF462	zinc finger protein 462 [Source:HGNC Symbol;Acc:HGNC:21684]	103,21	45,85	1,17	57,36	0,04
ZNF608	zinc finger protein 608 [Source:HGNC Symbol;Acc:HGNC:29238]	35,76	5,81	2,62	29,95	0,01
